# Associations of proteomic age clocks with lifestyle risk factors, incident chronic diseases and mortality in two European cohorts

**DOI:** 10.1038/s43587-026-01163-6

**Published:** 2026-06-29

**Authors:** Oliver Robinson, Han Xiao, Jan Homann, Vivian Viallon, Pietro Ferrari, Philipp Frank, José M. Huerta, Ana Jiménez Zabala, Rudolf Kaaks, Verena A. Katzke, Mika Kivimaki, Claudia Langenberg, Chung-Ho E. Lau, Lefkos Middleton, N. Charlotte Onland-Moret, Salvatore Panico, Anna Prizment, Fulvio Ricceri, María-José Sánchez, Karl Smith-Byrne, W. M. Monique Verschuren, Roel Vermeulen, Paolo Vineis, Shuo Wang, Nick Wareham, Christina M. Lill, Elio Riboli, Marc J. Gunter

**Affiliations:** 1 Department of Epidemiology and Biostatistics, School of Public Health, Imperial College London, London, UK; 2 Ageing Epidemiology Research (AGE) Unit, School of Public Health, Imperial College London, London, UK; 3 UK Dementia Research Institute at Imperial College London, London, UK; 4 Institute of Epidemiology and Social Medicine, University of Münster, Münster, Germany; 5 Nutrition and Metabolism Branch, International Agency for Research on Cancer, Lyon, France; 6 Brain Sciences, University College London, London, UK; 7 Department of Epidemiology, Murcia Regional Health Council-IMIB, Murcia, Spain; 8 Centro de Investigación Biomédica en Red de Epidemiología y Salud Pública (CIBERESP), Madrid, Spain; 9 Ministry of Health of the Basque Government, Sub Directorate for Public Health and Addictions of Gipuzkoa, San Sebastian, Spain; 10 Biogipuzkoa Health Research Institute, Epidemiology of Chronic and Communicable Diseases Group, San Sebastian, Spain; 11 Division of Cancer Epidemiology, German Cancer Research Center (DKFZ), Heidelberg, Germany; 12 Clinicum, Faculty of Medicine, University of Helsinki, Helsinki, Finland; 13 Precision Healthcare University Research Institute, Queen Mary University of London, London, UK; 14 MRC Epidemiology Unit, University of Cambridge, Cambridge, UK; 15 Computational Medicine, Berlin Institute of Health at Charité – Universitätsmedizin Berlin, Berlin, Germany; 16 Julius Center for Health Sciences and Primary Care, University Medical Center Utrecht, Utrecht University, Utrecht, the Netherlands; 17 Federico II University, Naples, Italy; 18 Department of Laboratory Medicine and Pathology, University of Minnesota, Minneapolis, MN USA; 19 Centre for Biostatistics, Epidemiology, and Public Health, Department of Clinical and Biological Sciences, University of Turin, Turin, Italy; 20 Escuela Andaluza de Salud Pública, Granada, Spain; 21 Instituto de Investigación Biosanitaria ibs.GRANADA, Granada, Spain; 22 Cancer Epidemiology Unit, University of Oxford, Oxford, UK; 23 National Institute for Public Health and the Environment, Bilthoven, the Netherlands; 24 Institute for Risk Assessment Sciences at Utrecht University, Utrecht, the Netherlands

**Keywords:** Predictive markers, Proteomics

## Abstract

Assessment of biological aging using proteomic clocks may enhance risk prediction and elucidate the molecular links between aging and chronic diseases. Here, among 17,473 participants of the European Prospective Investigation into Cancer and Nutrition, we examined associations of plasma SomaScan-based proteomic clocks, including organ-specific clocks, with risk factors, 24 incident chronic diseases and all-cause mortality, over up to 28 years of follow-up. Replication was conducted in the Whitehall II study. We show that the global age gap, an age acceleration score combining proteomic clocks, was associated with smoking, alcohol consumption, physical inactivity and higher risk of mortality, cardiovascular diseases, dementia and cancers of the liver, upper aero-digestive tract, lung and kidney. Lung, kidney and stomach cancers were more strongly associated with related organ-specific age gaps. Predictive performance of proteomic clocks for mortality was comparable to that of classical lifestyle risk factors. In summary, proteomic clocks appear promising biomarkers of generalized age-related disease risk.

## Abstract (web summary)

Robinson et al. provide an extensive evaluation of proteomic aging clocks in a large European sample, finding that these clocks were sensitive to lifestyle and could predict the risk of multiple diseases even around 20 years before onset.

## Main

Although age has long been recognized as the most important risk factor for most chronic diseases, it has only recently become a focus of risk factor epidemiology and interventional research, with the recognition that aging rates may be modifiable^1^. Geroscience proposes that biological aging, the cellular and molecular changes associated with aging, underlines the risk of multiple age-related diseases, and through targeting these processes, health in later life can be improved^2^. This is particularly relevant given the aging population and the challenge of closing the health span and lifespan gap^3^. To understand the role of biological aging in disease risk and the causes of variation in aging rates, scalable quantitative markers of biological age are required for use in human studies.

Since the introduction of the DNA methylation-based clock by Horvath^4^, multiple studies have developed biological age estimators (‘clocks’). They are built from multivariable models trained on chronological age using high-throughput molecular profiling or ‘omics’ data^5^. The clocks provide an average molecular profile at a given chronological age based on the reference training population. The difference between the clock age and chronological age (the ‘age gap’ or ‘age acceleration’) may then be interpreted as the biological age component of the clock prediction^5^. Plasma proteomics offers several advantages for assessing biological age, providing greater interpretability than epigenetic measures and benefiting from standardized analytic platforms. Circulating proteins also provide systematic assessment of tissues throughout the body^6^. In the UK Biobank, clocks have been developed using the Olink Proximity Extension Assay-based assay of around 3,000 proteins^7–9^ that predict age across external ethnically distinct populations^7^ and multiple incident diseases and mortality^7–9^. In parallel, multiple clocks have been developed using aptamer-based Somascan assays, with assay coverage ranging from around 1,300 (ref. ^10^) to 5,000 proteins^11–13^. These clocks have been trained in mainly healthy populations of differing age ranges and geographical location. Furthermore, Oh et al.^13^ developed organ-specific proteomics clocks, trained on proteins putatively annotated to their organ sources.

The Somascan-based clocks appear promising for the assessment of biological age and have been shown to predict mortality risk^12–15^ and/or age-related phenotypes^14–16^. However, only the organ-specific clocks developed by Oh et al.^13^ have been independently tested for risk of disease onset across multiple disease classes in a longitudinal setting^15^. In line with the geroscience hypothesis, proteomic clocks are expected to have stronger associations with diseases that most clearly increase in incidence with age. Furthermore, clocks trained in different populations may show differences in associations with risk factors and disease risk due to the characteristics of those populations. The European Prospective Investigation into Cancer and Nutrition (EPIC) Somalogic study employs a powerful case–cohort design to assess the risk of mortality and incidence of cardiometabolic diseases, neurodegenerative diseases and cancers in over 17,000 participants. Within EPIC, we sought to comprehensively assess associations of five previously published proteomic clocks and organ-specific clocks with risk of mortality and 24 disease endpoints over up to 28 years of follow-up. We compared the strengths of associations across different clocks and all endpoints and further explored whether combining age predictions from established clocks into a ‘Global’ ensemble clock may improve risk associations. The findings on mortality were then replicated in the British Whitehall II study. Finally, we tested associations of clocks with lifestyle risk factors, including a healthy lifestyle index (HLI).

## Results

### Study population

**Fig. 1:**
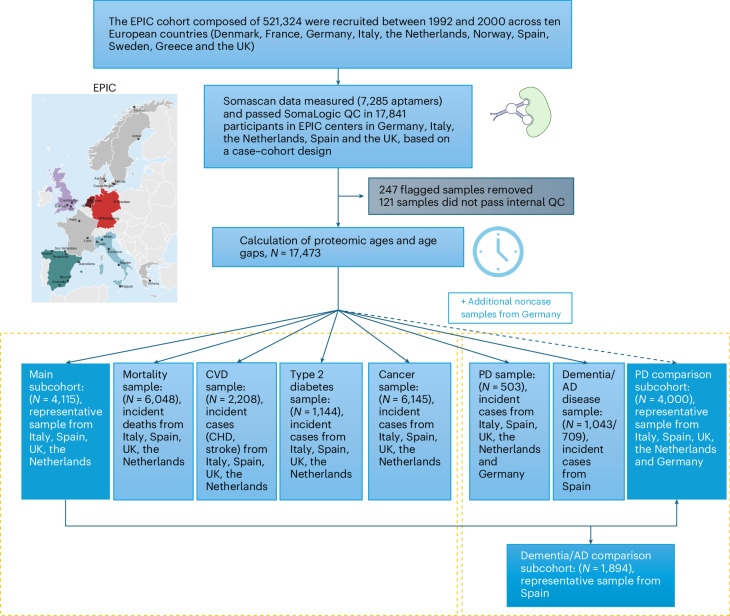
Study design and sampling flow chart in the EPIC study. CVD, cardiovascular disease; CHD, coronary heart disease. Dotted yellow boxes represent the two arms of the EPIC Somascan study: the neurodegenerative arm and the main arm that assesses risk of mortality, cancers, cardiovascular diseases and type 2 diabetes. The comparison subcohort in each arm differs, with the subcohort samples in the neurodegenerative arm including both samples from the main arm and additional noncase samples from Germany (dotted blue arrow). Figure created in BioRender; Xiao, H. https://biorender.com/mohxknd (2026).

This study analyzed proteomic data from baseline plasma samples on 17,473 participants drawn from Italy, Spain, the Netherlands, the UK and Germany using a case–cohort design for multiple disease outcomes (Fig. 1). The random subcohort sample set (*N* = 4,115), broadly representative of the wider EPIC cohort, served as the control set for different case samples sets and included naturally occurring incident diseases and deaths (Supplementary Table 1). The mean age in the subcohort was 51.4 (standard deviation (s.d.) 8.6) years, and 62% were female. The mean body mass index (BMI) was 26.9 (4.3) kg m^−2^, 25% were smokers and 14% had a university-level education. During a mean follow-up time of 17.4 (2.9) years, 393 (9.6%) deaths occurred in the subcohort. Separate case samples included 6,048 incident cases for mortality, 6,146 for cancers (17 cancer types), 2,208 for cardiovascular disease (coronary heart disease and stroke), 1,114 for type 2 diabetes and 1,546 for neurodegenerative diseases (all-cause dementia, Alzheimer’s disease (AD) and Parkinson’s disease (PD)). The neurodegenerative disease arm of this study involved a slightly different composition of participating centers per disease and comparison subcohorts (Fig. 1). Population characteristics of all the cases sample sets, and the comparison subcohorts used for the neurodegenerative disease analyses, are presented in Supplementary Tables 2–9.

We replicated the main analyses on mortality in an independent cohort, the British Whitehall II study, in which plasma samples for proteomic analyses were collected in 1997–1999. The analytic sample comprised 6,240 participants (mean age 56.1 (6.0) years, 28.8% women) who were followed up for mortality over a mean of 23.0 (4.8) years through linked electronic health records, during which 1,643 participants died.

### Proteomic age calculation

**Fig. 2:**
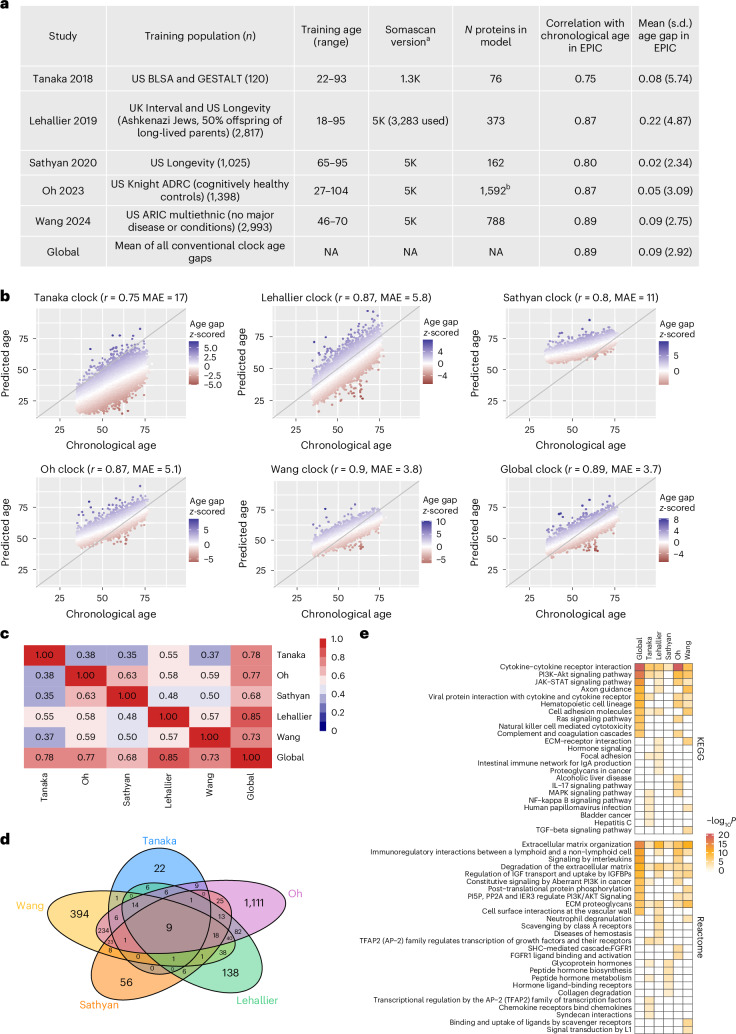
Overview of proteomic clocks. **a**, Table of clock characteristics. NA, not applicable. ^a^Approximate number of aptamers in version. ^b^Across 500 bootstrapped conventional age models. **b**, Scatterplots of predicted versus chronological age for all clocks used in the EPIC study, showing Pearson’s correlations and mean absolute error (MAE). **c**, Correlation heatmap of proteomic age gaps. **d**, Venn diagram showing overlap of proteins included in each clock. **e**, Enrichment of KEGG and Reactome pathways among proteins contained in the conventional clocks and the Global age clock. Pathway enrichment was tested using a two-sided Fisher’s exact test and adjusted for multiple comparisons using FDR correction. The top ten pathways passing FDR correction for each clock are shown. Enrichment of GO terms is shown in Supplementary Fig. 1. ECM, extracellular matrix; MAPK, mitogen-activated protein kinase pathway; NF, nuclear factor; TGF, transforming growth factor; IGF, insulin-like growth factor; IGFBPs, insulin-like growth factor binding proteins, SHC, SHC adaptor protein 1. Source data

The characteristics of five ‘conventional’ clocks included in the study, Tanaka^10^, Lehallier^16^, Sathyan^11^, Oh^13^ and Wang^12^, are shown in Fig. 2a and further described in the Methods. We included all published Somascan-based clocks, considered conventional as they were developed using all available proteins, as opposed to preselecting proteins as for organ-specific clocks, and trained directly on chronological age. The clock-predicted ages were all strongly correlated with chronological age in the full EPIC sample, ranging from *r* = 0.75 for the Tanaka clock to *r* = 0.89 for the Wang clock (Fig. 2a,b). The Tanaka and Lehallier clocks both provided an underprediction of age in EPIC. These clocks were trained on Somascan data for which the intensity values of the protein data in EPIC could not be appropriately scaled, due to use of an earlier Somascan version (Tanaka) or within population mean scaling (Lehallier). Furthermore, all clocks to varying degrees exhibited age dependency in their predictive accuracy (Fig. 2b), an issue observed generally in age prediction models^17^. These technical biases were corrected for by fitting a local regression line to derive an age gap representing the difference between proteomic and chronological age. The age gap s.d. ranged from 5.7 years for the Tanaka clock to 2.3 years for the Sathyan clock (Fig. 2a). Age gaps were moderately correlated across the conventional clocks, ranging from 0.35 (95% confidence interval (CI): 0.34–0.36) for Tanaka and Sathyan age gaps to 0.63 (95% CI 0.62 to 0.64) for Sathyan and Oh age gaps (Fig. 2c). Only nine proteins (ISLR2, EGFR, PTN, ADAMTS5, GDF15, MMP12, CDON, LPO and RSPO4; Fig. 2d) were shared as predictors across published clocks. To provide a single proteomic age estimation, we averaged both the proteomic ages and age gaps across the clocks to develop a ‘Global proteomic clock’. The correlation of Global proteomic age with chronological age was 0.89. Correlations of the Global age gap with the individual age gaps ranged from 0.68 (95% CI 0.67 to 0.69) for the Sathyan clock to 0.85 (95% CI 0.85 to 0.86) for the Lehallier clock (Fig. 2c).

Overrepresentation analysis of proteins included in each clock against Reactome, Kyoto Encyclopedia of Genes and Genomes (KEGG) and Gene Ontology (GO) databases, identified the ‘cytokine–cytokine receptor interaction’ KEGG pathway and ‘extracellular matrix’ related Reactome pathways as significantly enriched across all clocks (Fig. 2e). Enriched GO terms across all clocks included ‘collagen-containing extracellular matrix’, ‘glycosaminoglycan binding’ and ‘hormone activity’ (Supplementary Fig. 1). Enrichment was generally stronger for the Global clock, which included proteins from each clock, covering processes related to extracellular matrix, immune and inflammatory processes, axon guidance and insulin growth factor regulation (Fig. 2e).

### Associations of conventional proteomic age gaps with mortality and incident diseases

**Fig. 3:**
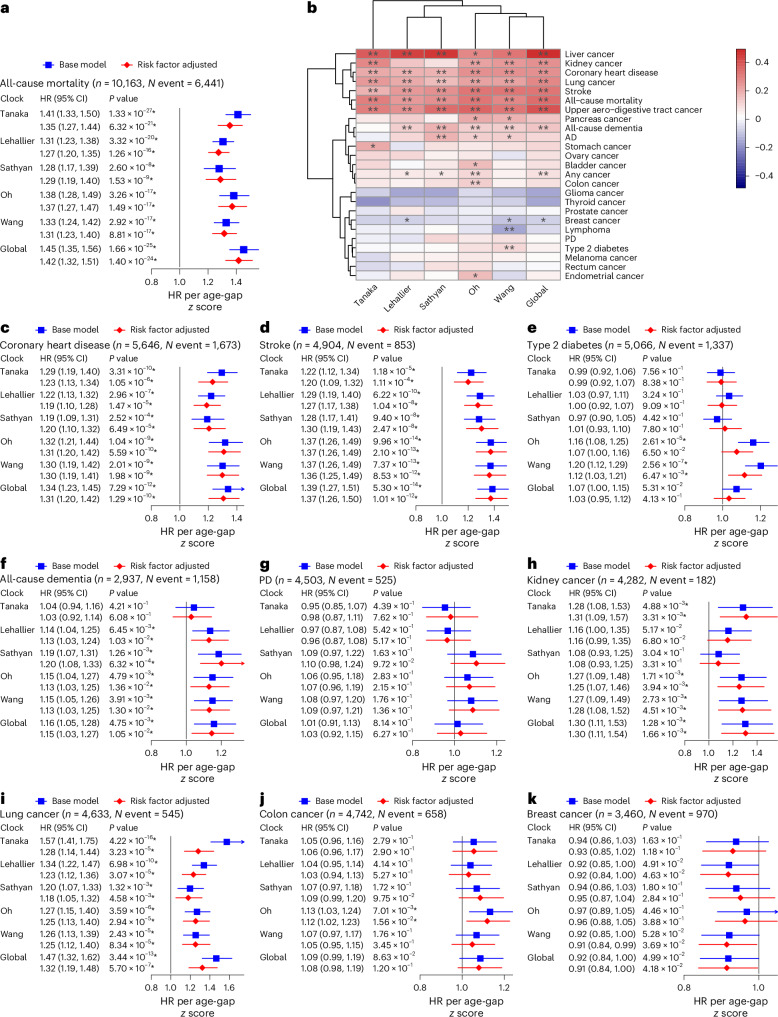
Associations of conventional proteomic age gaps with mortality and incident diseases. **a**, HRs per conventional proteomic age-gap *z* score with all-cause mortality. **b**, Clustered heatmap showing risk factor-adjusted associations (log hazards per age-gap *z* score) for all-cause mortality and 25 incident diseases. *P < 0.05; **FDR-adjusted *P* < 0.05. **c**–**k**, HRs per conventional proteomic age-gap *z* score with coronary heart disease (**c**), stroke (**d**), type 2 diabetes (**e**), all-cause dementia (**f**), PD (**g**), and cancers of the kidney (**h**), lung (**i**), colon (**j**) and breast (**k**). All Cox proportional hazards regression models were stratified by study center, sex and 5-year age group. The risk factor-adjusted model was additionally adjusted for education level, smoking status, alcohol consumption, BMI, healthy diet score and physical activity. In **a** and **c**–**k**, the center of each point represents the estimated HR per 1-s.d. increase in proteomic age-gap *z* score, and the error bars represent 95% CIs. *N* event refers to number of incident outcome events. *FDR-adjusted *P* < 0.05 (**a** and **c**–**k**). Source data

All conventional age gaps were significantly associated with all-cause mortality after false discovery rate (FDR) correction, and estimates were similar in both the base model (stratified by study center, sex and 5-year age group) and the risk factor-adjusted model (additionally adjusted for smoking status, alcohol consumption, BMI, healthy diet score and physical activity). Estimates across the five conventional clocks were similar, ranging from a hazard ratio (HR) of 1.27 (95% CI 1.20 to 1.35) for the Lehallier age-gap *z* score (that is, one s.d. in proteomic age gap, equivalent to around 5-year age gap for this clock) to an HR of 1.37 (95% CI 1.27 to 1.47) for the Oh age-gap *z* score (approximately 3-year age gap) in the risk factor-adjusted models. Notably, a stronger association with all-cause mortality was observed for the Global proteomic clock, with an HR of 1.42 (95% CI 1.32 to 1.51) per age-gap *z* score (approximately 3-year age gap) in the risk factor-adjusted model (Fig. 3a). In terms of increased mortality risk per year of age gap, estimates ranged from 5% for the Tanaka clock to a 13% greater risk for the Global proteomic clock (Supplementary Fig. 2)

Generally, a similar pattern of associations with disease was observed across the clocks (Fig. 3b). All clocks were positively associated with risk of coronary heart disease (Fig. 3c) and stroke (Fig. 3d). Slightly larger effect sizes were observed for the Global proteomic age gap with HRs of 1.31 (95% CI 1.20 to 1.42) for coronary heart disease and 1.37 (95% CI 1.26 to 1.50) for stroke in the risk factor-adjusted model. Associations with type 2 diabetes were inconsistent across clocks and greater differences between the base and risk factor adjusted models were observed.

Five clocks were associated with all-cause dementia (Fig. 3f), with a risk factor-adjusted HR of 1.15 (95% CI 1.03 to 1.27) for all-cause dementia per Global age-gap *z* score. For AD, all but the Tanaka clock showed positive directions of effect, with only the association with the Sathyan clock (HR 1.20; 95% CI 1.07 to 1.34) reaching FDR-corrected significance. Associations with PD (Fig. 3g) were generally inconsistent and nonsignificant across the clocks.

Both the Oh and Global proteomic clocks were significantly associated with the incidence of any cancer (Fig. 3b). All clocks were associated with lung cancer (Fig. 3i) and upper aero-digestive tract (UDAT) cancer (Fig. 3b), and four clocks were associated with liver and kidney cancer (Fig. 3h) in risk factor-adjusted models. Again, for these outcomes, often slightly larger effect sizes were observed for the Global proteomic age gap, with HRs of 1.32 (95% CI 1.19 to 1.48) for lung cancer, 1.50 (95% CI 1.29 to 1.74) for UDAT cancer, 1.63 (95% CI 1.25 to 2.12) for liver cancer and 1.30 (95% CI 1.11 to 1.54) for kidney cancer per age-gap *z* score in risk factor-adjusted models. For colon, pancreas, stomach and bladder cancers, generally positive directions of association were observed across the clocks, with only the association between the Oh clock and colon cancer (Fig. 3j) significant after FDR correction. For glioma, thyroid, prostate, breast and lymphoma cancers, generally inverse directions of effect were observed across the clocks.

### Associations of conventional proteomic age gaps with mortality in the Whitehall II study

**Fig. 4:**
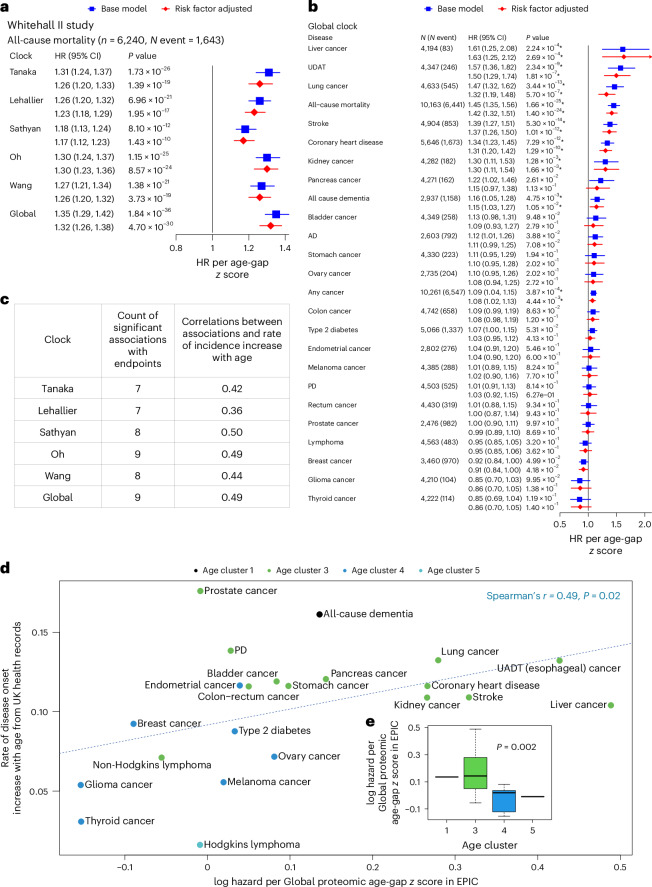
Comparison of strengths of associations with Global proteomic age across disease endpoints. **a**, HRs per conventional proteomic age-gap *z* score with all-cause mortality, in the Whitehall II study. Base model stratified by sex and 5-year age group. The risk factor-adjusted model was additionally adjusted for education level, smoking status, alcohol consumption, BMI, fruit and vegetable consumption and meeting WHO physical activity recommendation. **b**, HRs per Global proteomic age-gap *z* score with all-cause mortality and 25 incident diseases in the EPIC study. All models were stratified by study center, sex and 5-year age group. The risk factor-adjusted model was additionally adjusted for education level, smoking status, alcohol consumption, BMI, healthy diet score and physical activity. *FDR-adjusted *P* < 0.05. The center of each point represents the estimated HR per 1-s.d. increase in proteomic age-gap *z* score, and the error bars represent 95% CIs (**a** and **b**). **c**, Comparison of clocks based on the number of significant positive associations (after FDR correction in the risk factor-adjusted model in the EPIC study) with endpoints, and Spearman’s correlations between log hazard per proteomic age-gap *z* score and the age-related increase in disease incidence rates from UK National Health records across 22 diseases. **d**, Scatterplot of log hazards per Global proteomic age-gap *z* score (risk factor-adjusted model in the EPIC study) against rate of disease incidence increase with age from UK National health records for 22 diseases. Correlation was assessed using a two-sided Spearman’s correlation. Disease points are labeled and colored by age cluster as reported by Kuan et al.^18^ Each point corresponds to one independently estimated disease-specific log HR. **e**, Boxplots showing log hazards per Global proteomic age-gap *z* score (risk factor-adjusted model) within each age cluster. *P* value calculated using a two-sided *t*-test comparing log hazards between cluster 3 diseases (*n* = 13) versus cluster 4 diseases (*n* = 7). Boxplots indicate the median, interquartile range and whiskers (median ± 1.5× interquartile range). Rates of incidence increase and cluster information were extracted from the work of Kuan et al.^18^. Source data

To replicate the observed associations with mortality, we calculated the conventional clocks and the Global age clock in the Whitehall II study. Clock-predicted ages were all strongly correlated with chronological age (*r* = 0.61 for the Sathyan clock to *r* = 0.84 for the Wang clock; Supplementary Fig. 3). Associations of all six clocks with all-cause mortality were replicated in both the base model and the risk factor-adjusted model. In the risk factor-adjusted model, HRs for the clocks ranged from 1.17 (95% CI 1.12 to 1.23) for the Sathyan age-gap *z* score to 1.32 (95% CI 1.26 to 1.38) for the Global age clock *z* score (Fig. 4a).

### Comparison of strengths of associations with Global proteomic age across disease endpoints

**Extended Data Fig. 1:**
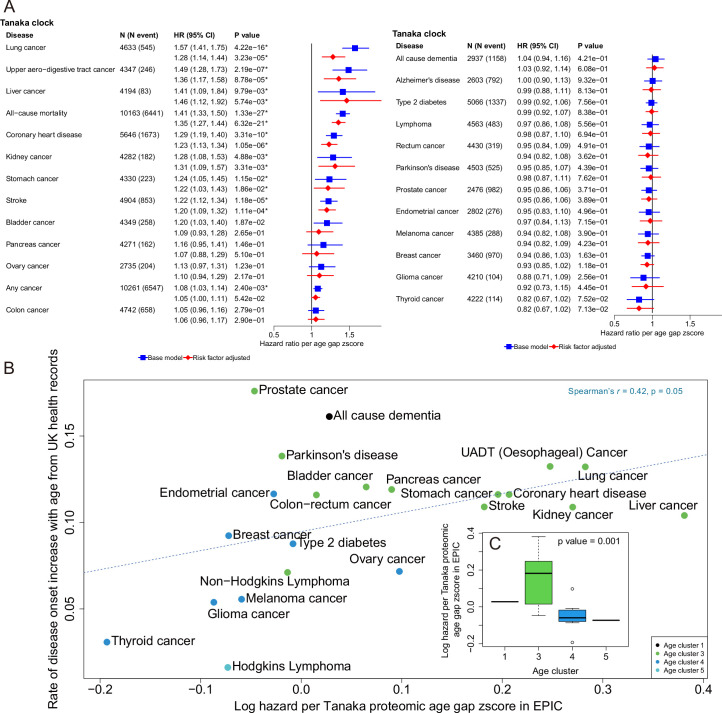
Comparison of strengths of associations with Tanaka proteomic age across disease endpoints. **a**: Hazard ratios per Tanaka proteomic age gap z-score with all -cause mortality and 25 incident diseases. All models stratified by study centre, sex, and five-year age group. Risk factor adjusted model additionally adjusted for education level, smoking status, alcohol consumption, BMI, healthy diet score and physical activity. Centre of each point represents the estimated hazard ratio per 1-SD increase in proteomic age gap z-score, and the error bars represent 95% confidence intervals. *FDR-adjusted P < 0.05 **b**: Scatterplot of log hazards per Tanaka proteomic age gap z-score (risk factor adjusted model in the EPIC study) against rate of disease incidence increase with age from UK National health records for 22 diseases. Correlation shows two-sided Spearman’s correlation. Disease points are labelled and colored by age cluster as reported by Kuan et al.^18^ **c**: Boxplots showing log hazards per Tanaka proteomic age gap z-score (risk factor adjusted model) within each age cluster. P value calculated from two-sided t-test comparing log hazards for cluster 3 diseases (n = 13) versus cluster 4 diseases (n = 7). Boxplots indicate the median, interquartile range, and whiskers (median ± 1.5 × IQR). Rate of incidence increases and cluster information extracted from Kuan et al.^18^. Source data

**Extended Data Fig. 2:**
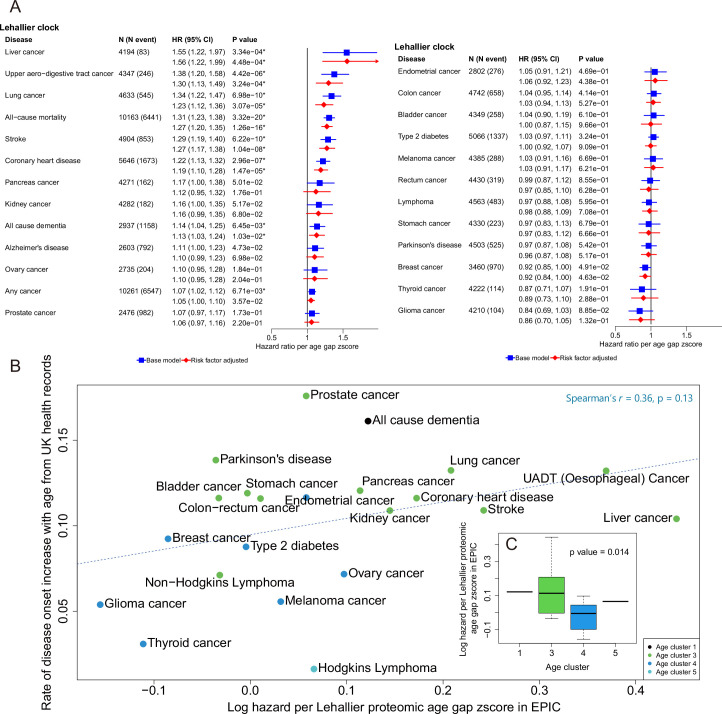
Comparison of strengths of associations with Lehallier proteomic age across disease endpoints. **a**: Hazard ratios per Lehallier proteomic age gap z-score with all -cause mortality and 25 incident diseases. All models stratified by study centre, sex, and five-year age group. Risk factor adjusted model additionally adjusted for education level, smoking status, alcohol consumption, BMI, healthy diet score and physical activity. Centre of each point represents the estimated hazard ratio per 1-SD increase in proteomic age gap z-score, and the error bars represent 95% confidence intervals. *FDR-adjusted P <0.05. **b**: Scatterplot of log hazards per Lehallier proteomic age gap z-score (risk factor adjusted model in the EPIC study) against rate of disease incidence increase with age from UK National health records for 22 diseases. Correlation shows two-sided Spearman’s correlation. Disease points are labelled and colored by age cluster as reported by Kuan et al.^18^ **c**: Boxplots showing log hazards per Lehallier proteomic age gap z-score (risk factor adjusted model) within each age cluster. P value calculated from two-sided t-test comparing log hazards for cluster 3 diseases (n = 13) versus cluster 4 diseases (n = 7). Boxplots indicate the median, interquartile range, and whiskers (median ± 1.5 × IQR). Rate of incidence increases and cluster information extracted from Kuan et al.^18^. Source data

**Extended Data Fig. 3:**
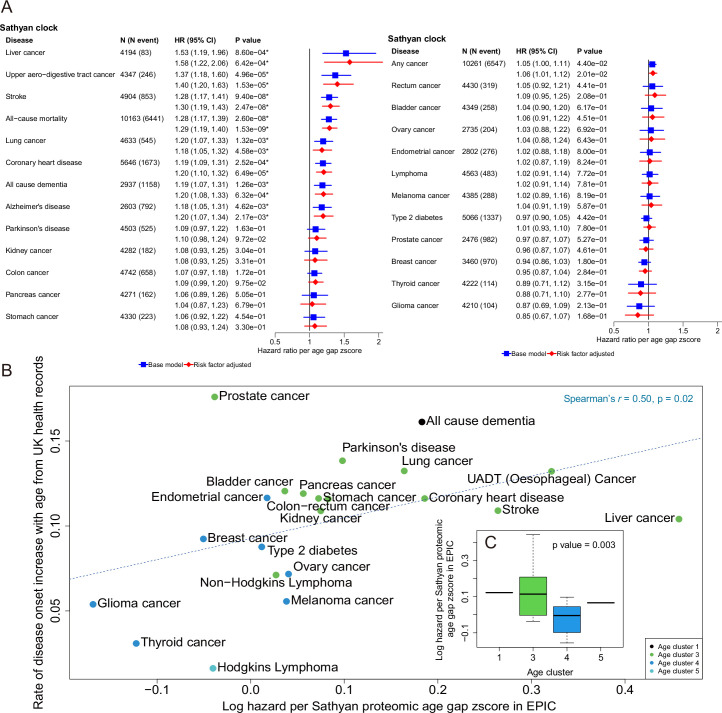
Comparison of strengths of associations with Sathyan proteomic age across disease endpoints. **a**: Hazard ratios per Sathyan proteomic age gap z-score with all -cause mortality and 25 incident diseases. All models stratified by study centre, sex, and five-year age group. Risk factor adjusted model additionally adjusted for education level, smoking status, alcohol consumption, BMI, healthy diet score and physical activity. Centre of each point represents the estimated hazard ratio per 1-SD increase in proteomic age gap z-score, and the error bars represent 95% confidence intervals. *FDR-adjusted P < 0.05. **b**: Scatterplot of log hazards per Sathyan proteomic age gap z-score (risk factor adjusted model in the EPIC study) against rate of disease incidence increase with age from UK National health records for 22 diseases. Correlation shows two-sided Spearman’s correlation. Disease points are labelled and colored by age cluster as reported by Kuan et al.^18^ **c**: Boxplots showing log hazards per Sathyan proteomic age gap z-score (risk factor adjusted model) within each age cluster. P value calculated from two-sided t-test comparing log hazards for cluster 3 diseases (n = 13) versus cluster 4 diseases (n = 7). Boxplots indicate the median, interquartile range, and whiskers (median ± 1.5 × IQR). Rate of incidence increases and cluster information extracted from Kuan et al.^18^. Source data

**Extended Data Fig. 4:**
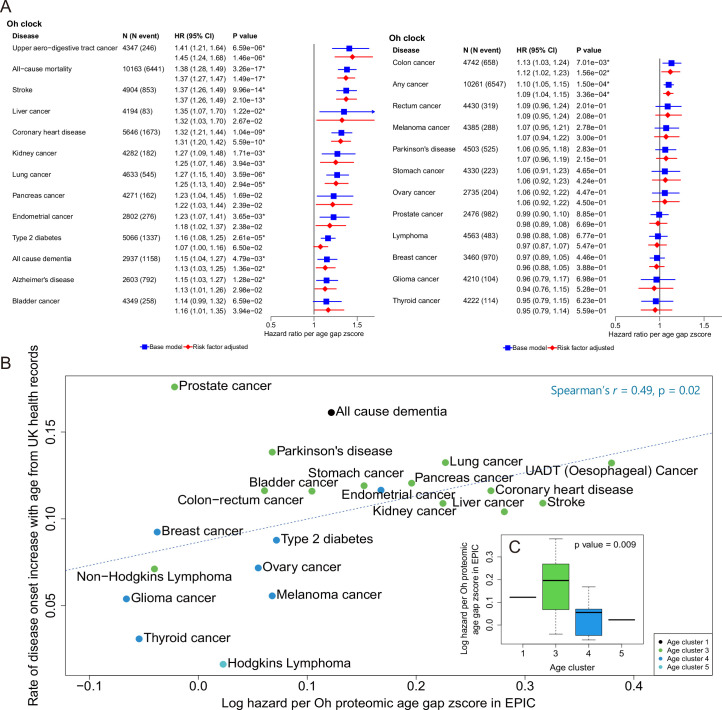
Comparison of strengths of associations with Oh proteomic age across disease endpoints. **a**: Hazard ratios per Oh proteomic age gap z-score with all -cause mortality and 25 incident diseases. All models stratified by study centre, sex, and five-year age group. Risk factor adjusted model additionally adjusted for education level, smoking status, alcohol consumption, BMI, healthy diet score and physical activity. Centre of each point represents the estimated hazard ratio per 1-SD increase in proteomic age gap z-score, and the error bars represent 95% confidence intervals. *FDR-adjusted P < 0.05. **b**: Scatterplot of log hazards per Oh proteomic age gap z-score (risk factor adjusted model in the EPIC study) against rate of disease incidence increase with age from UK National health records for 22 diseases. Correlation shows two-sided Spearman’s correlation. Disease points are labelled and colored by age cluster as reported by Kuan et al.^18^ **c**: Boxplots showing log hazards per Oh proteomic age gap z-score (risk factor adjusted model) within each age cluster. P value calculated from two-sided t-test comparing log hazards for cluster 3 diseases (n = 13) versus cluster 4 diseases (n = 7). Boxplots indicate the median, interquartile range, and whiskers (median ± 1.5 × IQR). Rate of incidence increases and cluster information extracted from Kuan et al.^18^. Source data

**Extended Data Fig. 5:**
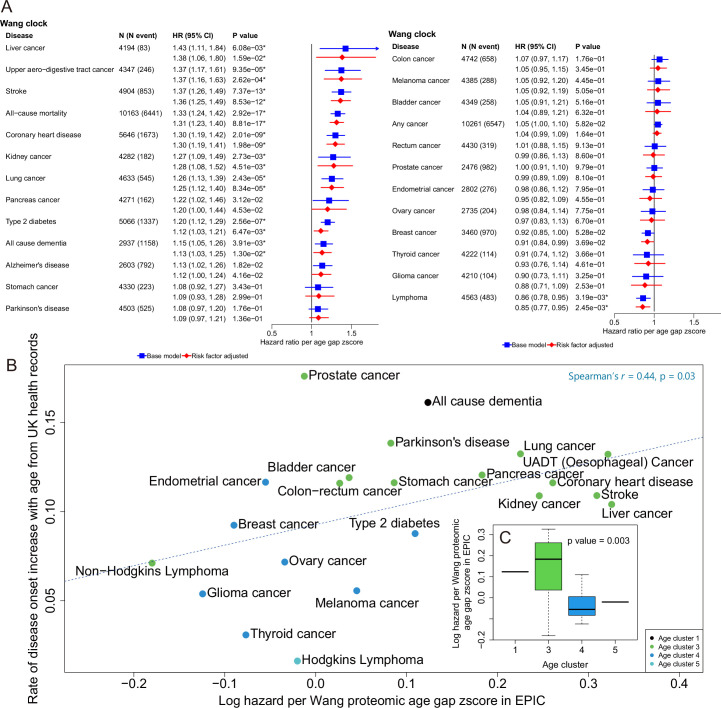
Comparison of strengths of associations with Wang proteomic age across disease endpoints. **a**: Hazard ratios per Wang proteomic age gap z-score with all -cause mortality and 25 incident diseases. All models stratified by study centre, sex, and five-year age group. Risk factor adjusted model additionally adjusted for education level, smoking status, alcohol consumption, BMI, healthy diet score and physical activity. Centre of each point represents the estimated hazard ratio per 1-SD increase in proteomic age gap z-score, and the error bars represent 95% confidence intervals. *FDR-adjusted P < 0.05. **b**: Scatterplot of log hazards per Wang proteomic age gap z-score (risk factor adjusted model in the EPIC study) against rate of disease incidence increase with age from UK National health records for 22 diseases. Correlation shows two- sided Spearman’s correlation. Disease points are labelled and colored by age cluster as reported by Kuan et al.^18^. **c**: Boxplots showing log hazards per Wang proteomic age gap z-score (risk factor adjusted model) within each age cluster. P value calculated from two-sided t-test comparing log hazards for cluster 3 diseases (n = 13) versus cluster 4 diseases (n = 7). Boxplots indicate the median, interquartile range, and whiskers (median ± 1.5 × IQR). Rate of incidence increases and cluster information extracted from Kuan et al.^18^. Source data

Figure 4b shows associations between the Global proteomic age gap and all disease endpoints. The largest effect size was observed for liver cancer, followed by UDAT cancer and then all-cause mortality. Nine out of 25 (36%) mortality and disease endpoints were positively associated with the Global age gap. To explore the heterogeneity in associations, we compared the strength of age gap associations observed in the EPIC study with associations with (chronological) age estimated from the UK electronic health records by Kuan et al.^18^. Among 22 disease endpoints that could be matched to diseases reported by Kuan et al.^18^ we observed a moderate positive correlation (Spearman’s *ρ* = 0.49, *P* = 0.02) between the log hazards per Global proteomic gap *z* score and the UK rates of disease incidence increase with age (Fig. 4d). Positive directions of association with the Global proteomic age gap *z* score were observed for almost all diseases in age clusters 1 and 3 (as defined by Kuan at al.^18^ as those with an exponential rate of disease onset increase with age; Fig. 4d,e), while generally weaker or even negative directions of effect were observed for diseases in age clusters 4 (diseases with a more gradual relationship with age) and 5 (not age-related). Similar patterns of associations were observed for the published conventional proteomic age gaps (Fig. 4c and Extended Data Figs. 1–5).

**Extended Data Fig. 6:**
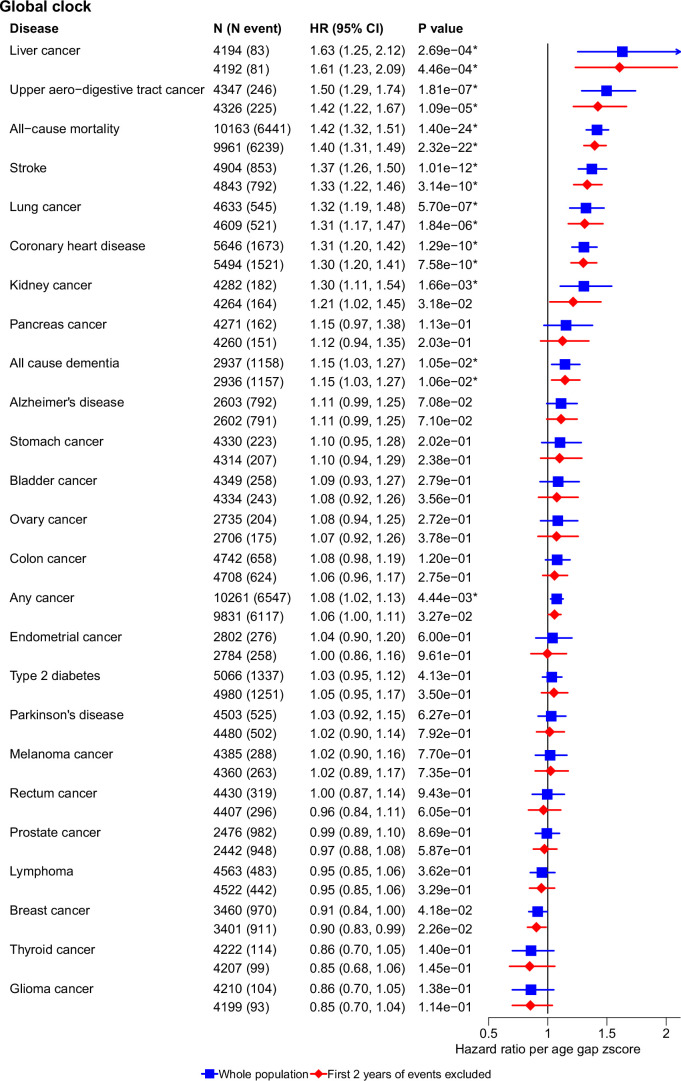
Associations with Global proteomic age across disease endpoints for all events (blue bars) and with events restricted only to events that occurred after two years from baseline (red bars). Hazard ratios per Global proteomic age gap z-score with all -cause mortality and 25 incident diseases. All Cox proportional hazards regression models were stratified by study centre, sex, and five-year age group and adjusted for education level, smoking status, alcohol consumption, BMI, healthy diet score and physical activity. The centre of each point represents the estimated hazard ratio per 1-SD increase in proteomic age gap z-score, and the error bars represent 95% confidence intervals. *FDR-adjusted P < 0.05. Source data

**Extended Data Fig. 7:**
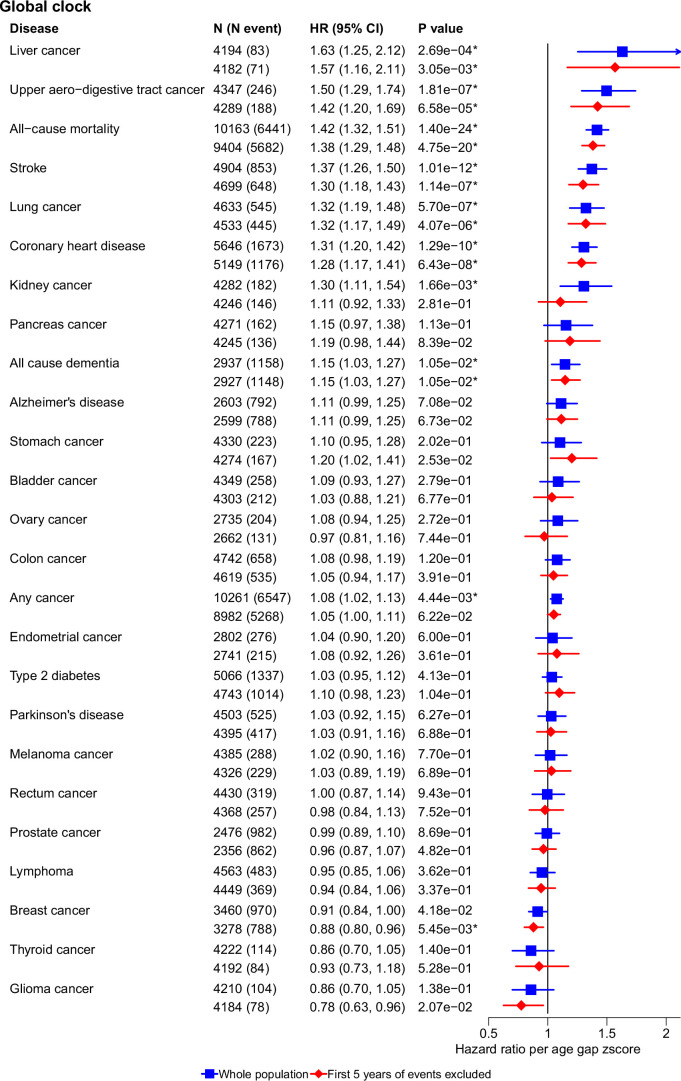
Associations with Global proteomic age across disease endpoints for all events (blue bars) and with events restricted only to events that occurred after five years from baseline (red bars). Hazard ratios per Global proteomic age gap z-score with all -cause mortality and 25 incident diseases. All Cox proportional hazards regression models were stratified by study centre, sex, and five-year age group and adjusted for education level, smoking status, alcohol consumption, BMI, healthy diet score and physical activity. The centre of each point represents the estimated hazard ratio per 1-SD increase in proteomic age gap z-score, and the error bars represent 95% confidence intervals. *FDR-adjusted P < 0.05. Source data

**Extended Data Fig. 8:**
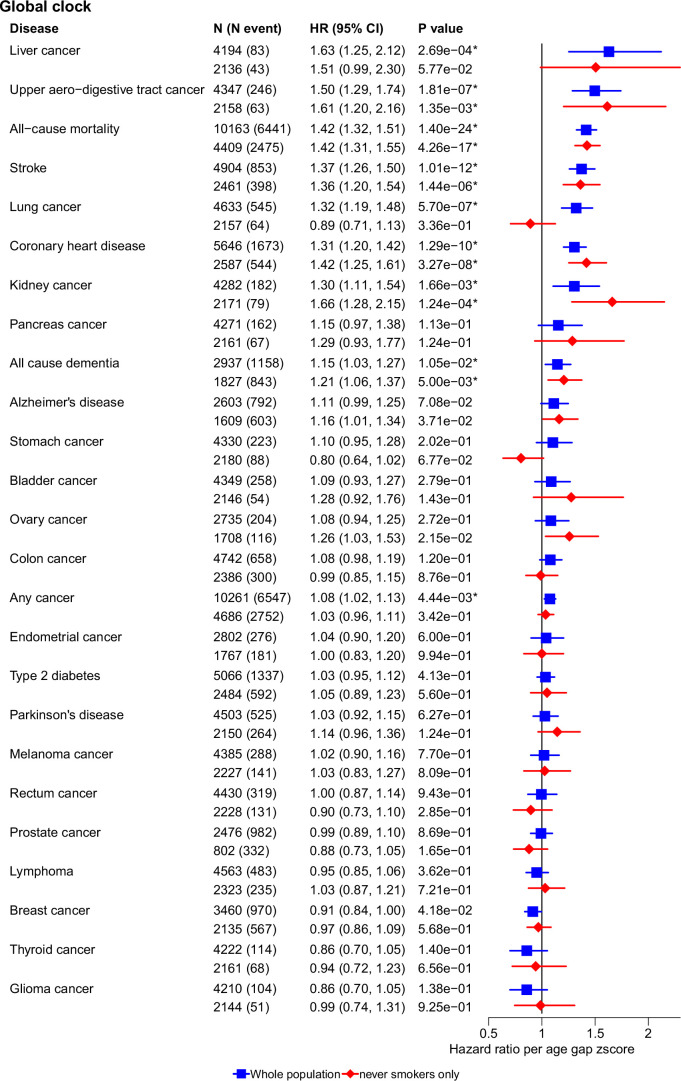
Associations with Global proteomic age across disease endpoints for whole population (blue bars) and for never smokers only (red bars). Hazard ratios per Global proteomic age gap z-score with all -cause mortality and 25 incident diseases. All Cox proportional hazards models were stratified by study centre, sex, and five-year age group and adjusted for education level, smoking status (whole population only), alcohol consumption, BMI, healthy diet score and physical activity. The centre of each point represents the estimated hazard ratio per 1-SD increase in proteomic age gap z-score, and the error bars represent 95% confidence intervals. *FDR-adjusted P < 0.05. Source data

In sensitivity analyses, we examined disease associations with the Global proteomic age gap restricting to events that occurred only after 2 years (Extended Data Fig. 6) and after 5 years (Extended Data Fig. 7) since recruitment. Estimates were similar except for some attenuation in the association with kidney cancer. We also restricted analyses to never-smokers only (Extended Data Fig. 8), again finding similar estimates, except for lung cancer, for which there were far fewer cases.

### Associations of risk factors with proteomic age

**Fig. 5:**
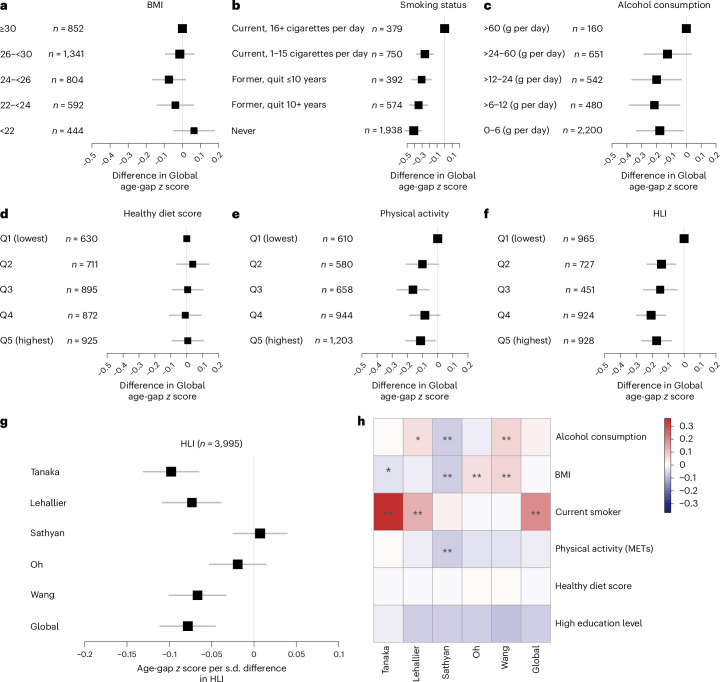
Associations of risk factors with conventional proteomic age gaps in the main subcohort. **a**–**f**, Associations of BMI (**a**), smoking status (**b**), alcohol consumption (**c**), healthy diet score (**d**), physical activity (**e**) and HLI (**f**) with the Global age-gap *z* score, adjusted for age, sex and study center. The reference category is always the least healthy category. Q, quintiles. **g**, Associations between continuous HLI *z* scores and conventional age-gap *z* scores, adjusted for age, sex and study center. All error bars in **a**–**g** show 95% CIs. **h**, Heatmap showing associations of risk and protective factors with conventional age-gap *z* scores. Estimates were derived from a mutually adjusted model including all factors shown, as well as age, sex and study center. Continuous factors (alcohol intake, BMI, physical activity and healthy diet score) are scaled to facilitate comparison. The reference group for current smokers was nonsmokers/former smokers. High education level refers to equivalent or university degree or higher, and the reference category is education up to technical or secondary school. *P < 0.05; **FDR-adjusted *P* < 0.05. *N* = 3,995. Source data

In analyses within the EPIC subcohort, we found that, compared with those smoking 16 cigarettes or more per day, never-smokers had, on average, 0.41 s.d. lower (95% CI −0.52 to −0.31; Fig. 5b) Global proteomic age gap. Drinking less than 6 g ethanol per day was associated with on average 0.18 s.d. lower (95% CI −0.33 to −0.02; Fig. 5c) Global age gap compared with drinkers of more than 60 g ethanol per day. The most physically active (top quintile) had on average 0.11 s.d. lower (95% CI −0.22 to −0.02; Fig. 5e) Global proteomic age gap compared with the most inactive (first quintile). Those in the highest quintile of the HLI, a 20-point scale based on diet quality, physical activity levels, smoking history, alcohol consumption and BMI, had on average 0.17 s.d. lower (95% CI −0.26 to −0.08) Global proteomic age gap compared with the lowest quintile (Fig. 5f). No associations were observed with BMI (Fig. 5a) and diet (Fig. 5d).

The HLI was inversely associated with age-gap *z* score for all conventional proteomic clocks, except the Oh and Sathyan clocks (Fig. 5g). These differences are probably driven by inconsistencies across the clocks in their association with individual noncommunicable disease risk factors (Fig. 5h). Alcohol consumption (as a continuous variable) was associated with increased age gap for the Lehallier and Wang clocks only. BMI *z* score was associated with increased age gap for the Oh and Wang clocks and with lower age gap for the Sathyan clock, while current smoking was associated with increased age gap for the Lehallier, Tanaka and Global clocks only. Among protective factors, including physical activity, healthy diet score and high education level, associations were generally in the expected direction; however, only the association between physical activity (as a continuous variable) and lower age-gap *z* scores for the Sathyan clocks remained significant after FDR correction.

### Associations of organ-specific proteomic age gaps with mortality and incident diseases

**Fig. 6:**
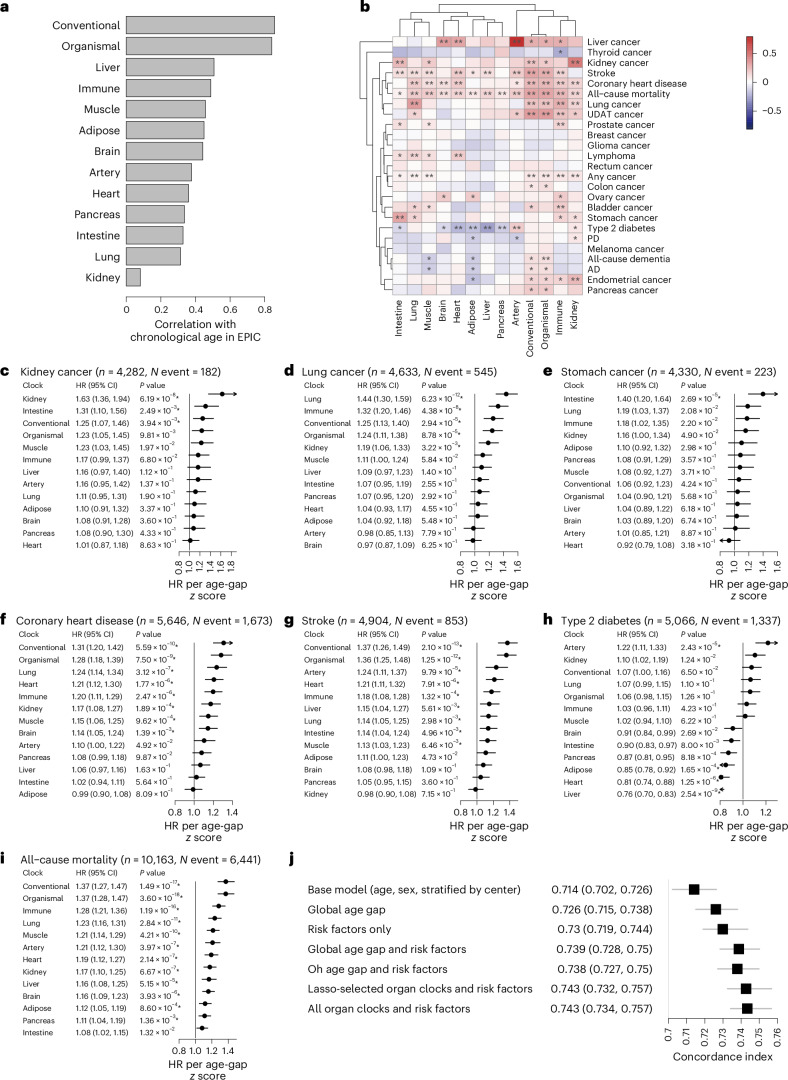
Associations of organ-specific proteomic age gaps with mortality and incident diseases. All models were generated using weights from the study of Oh et al. Here, we refer to the Oh clock, as previously presented, as ‘conventional’. **a**, Pearson’s correlations with chronological age in all EPIC samples. **b**, Clustered heatmap showing risk factor-adjusted associations (log hazards per age-gap *z* score) for all-cause mortality and 25 incident diseases. *P < 0.05; **FDR-adjusted *P* < 0.05. **c**–**i**, HRs from risk factor adjusted models per organ-specific proteomic age-gap *z* score with kidney cancer (**c**), lung cancer (**d**), stomach cancer (**e**), CHD (**f**), stroke (**g**), type 2 diabetes (**h**) and all-cause mortality (**i**). Models were stratified by study center, sex and 5-year age group and adjusted for education level, smoking status, alcohol consumption, BMI, healthy diet score and physical activity. In **c**–**i**, the center of each point represents the estimated HR per 1-s.d. increase in proteomic age-gap *z* score, and the error bars represent 95% CIs. *FDR-adjusted *P* < 0.05 (**c**–**i**). **j**, Comparison of discriminatory power showing concordance index for various models for prediction of mortality (*n* = 10,163). The base model included chronological age and sex and was stratified by study center; other models additionally included standard risk factors, the Global age gap, the Oh age gap, lasso-selected organ clocks or all organ clocks. A log-likelihood ratio test was used to compare different mortality prediction models. All error bars show 95% CIs. Source data

**Extended Data Fig. 9:**
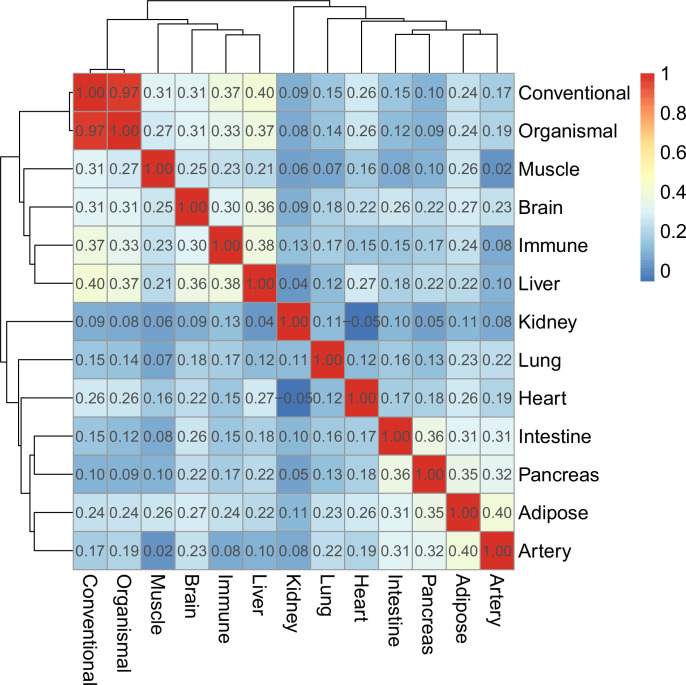
Correlation heatmap of organ specific proteomic age gaps. Pearson’s correlations are shown. Source data

In EPIC, organ-specific clocks were calculated using the weights generated by the study of Oh et al.^13^ trained in the same population as the Oh clock (Fig. 2). In the following section, we have included the Oh clock as a comparison, referred to as a ‘Conventional’ clock. All clock-predicted ages were significantly associated (*P* < 2.2 × 10^−16^) with chronological age (Fig. 6a). Conventional and organismal age gaps were very strongly correlated with each other (*r* = 0.97, 95% CI 0.97 to 0.97), while other correlations between the organ-specific age gaps were weak to moderate, ranging from −0.05 (kidney–heart, 95% CI −0.07 to −0.04) to 0.40 (artery–adipose, 95% CI 0.39 to 0.41) (Extended Data Fig. 9).

After FDR correction, the immune clock was positively associated with the most endpoints with eight significant associations, followed by the organismal age and conventional age gaps with seven associations, lung and kidney age gaps with six each, and the heart age gaps with five (Fig. 6b). In some cases, organ-specific clocks showed stronger associations with diseases related to their organ such as for the kidney age gap and kidney cancer (HR 1.63, 95% CI 1.36 to 1.94; Fig. 6c), lung age gap and lung cancer (HR 1.44, 95% CI 1.30 to 1.59; Fig. 6d) and intestine age gap and stomach cancer (HR 1.40, 95% CI 1.20 to 1.64; Fig. 6e). The heart age gap was most strongly associated with coronary heart disease (HR 1.21, 95% CI 1.12 to 1.30) after the conventional, organismal and lung clocks (Fig. 6f), while the artery clock was most strongly associated organ-specific clock with stroke (HR 1.24, 95% CI 1.11 to 1.37; Fig. 6g). The brain age clock was not associated with any of the neurodegenerative diseases. While there was a nominally significant association between the kidney clock and type 2 diabetes, type 2 diabetes was negatively associated with multiple organ clocks including the pancreas age gap. We observed that the kidney clock clustered most closely with the immune, conventional and organismal clocks (Fig. 6b).

**Extended Data Fig. 10:**
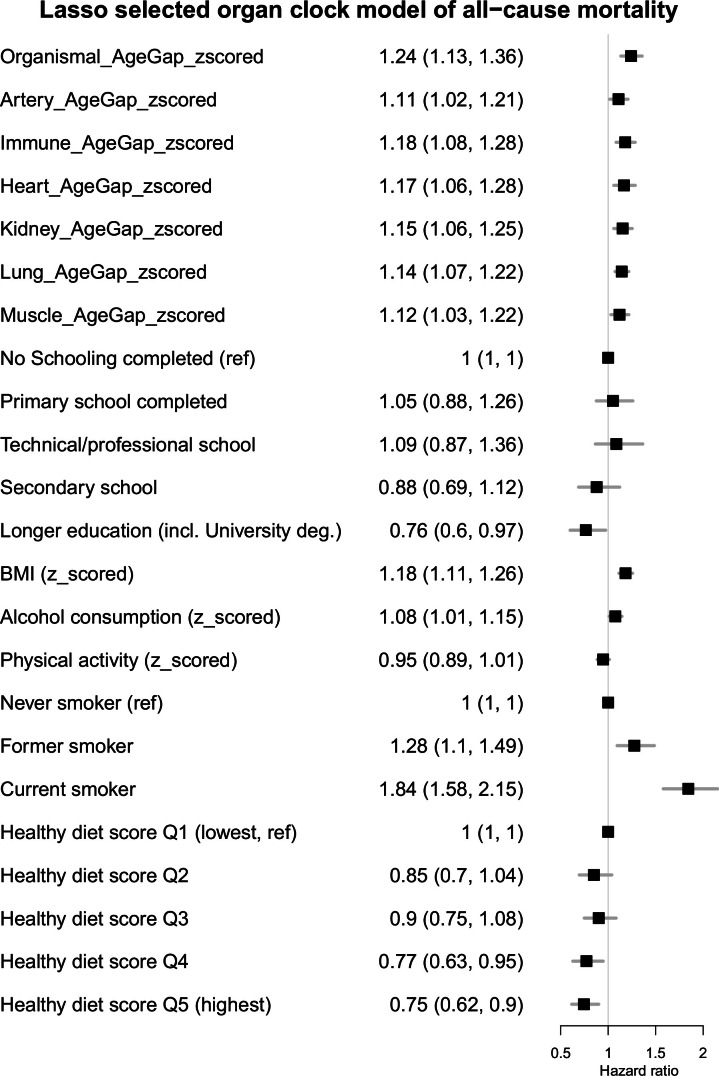
Hazard ratios from lasso selected combined Cox model of all-cause mortality. Model stratified by study centre, sex, and five-year age group and includes all covariates shown. The centre of each point represents the estimated hazard ratio covariate (per SD for continuous variable or compared to reference category for categorical), and the error bars represent 95% confidence intervals. Source data

All but the intestine age gaps were associated with mortality (Fig. 6i). Lasso penalization was applied to select a risk factor-adjusted model that included only organ age gaps with significant independent associations with mortality. The final model included organismal, artery, immune, heart, kidney, lung and muscle age gaps (Extended Data Fig. 10). All associations were attenuated in this combined model. For instance, the organismal and kidney age gaps had HRs of 1.37 (95% CI 1.28 to 1.47) and 1.17 (95% CI 1.10 to 1.25), respectively, in the single-organ age models (Fig. 6i) and HRs of 1.24 (95% CI 1.13–1.36) and 1.15 (95% CI 1.06–1.25), respectively, in the lasso-selected model (Supplementary Fig. 13)

### Comparison of mortality prediction models

The concordance index of a base model predictive of all-cause mortality containing chronological age and sex (stratified by study center) was 0.71 (95% CI 0.70 to 0.73). Upon addition of education level, smoking status, alcohol consumption, physical activity, BMI and healthy diet index, the concordance index improved to 0.73 (95% CI 0.72 to 0.74), similar to the model including only the Global age gap (0.73, 95% CI 0.72 to 0.74). The concordance index improved to 0.74 (95% CI 0.73 to 0.75), when risk factors and the Global age gap were combined (Fig. 6j). The Global age gap and risk factor model demonstrated a significant improvement in model fit compared with the risk-factor-only model (log-likelihood ratio test *P* < 2.2 × 10^−16^). Similar concordance indices were observed with the conventional age gaps (Supplementary Fig. 4), the lasso-selected organ age model and a model containing all organismal and organ-specific age gaps (Fig. 6j).

## Discussion

In this large pan-European cohort with up to 28 years of follow-up, we conducted an extensive assessment of proteomic age gaps as markers for accelerated aging in relation to mortality and 24 incident diseases. We have shown that Somascan-based proteomic age gaps are associated with all-cause mortality risk across all proteomic clocks tested, indicating their utility as biological age metrics across independent populations. We found that the Global age gap, an unweighted average of age gaps derived from five previously developed clocks, was marginally more strongly associated with mortality and many incident diseases than the individual clock estimates. The Global age gap was associated with age-related diseases including cardiovascular diseases, dementia and cancers of the liver, UDAT, lung and kidney. We observed a significant inverse association between the HLI and the Global age gap, suggesting that improving health behaviors may improve aging trajectories. We found that proteomic age provided similar performance in predicting mortality to classical lifestyle risk factors, and that predictive power was further enhanced when risk factors and proteomic age gaps were combined, indicating that proteomic age provides additional information on mortality risk. We further observed that cancers of the kidney, stomach and lungs were most strongly predicted by the proteomic age gaps in related organs.

Our analysis showed that each additional year of Global age gap was associated with a 13% increase in mortality risk. Based on a conversion formula^19^ derived from UK lifetables, we estimate that this is equivalent to approximately 6 months of life lost for every year of additional age gap. This is in line with the range of effects sizes for all-cause mortality reported in previous studies^7,11–14^, demonstrating the consistency of effects observed across proteomic clocks and populations. Although only a limited number of proteins were common across all conventional clocks, the moderate correlations and similar disease associations across the clocks suggest that they are capturing a high-level summary of multiple intrinsic ageing processes^20^. Overrepresentation analysis, which is partially driven by the proteomic coverage of the Somascan platforms, also indicated shared pathways related to the extracellular matrix, inflammation and hormonal activity across the tested clocks. The Global age clock was enriched across multiple pathways well recognized to relate to different facets of biological aging, including additional pathways related to axon guidance and insulin growth factor regulation.

As measured by the Global proteomic age, we observed that heavy smokers were, on average, 14 months biologically older than never-smokers, heavy drinkers were around 7 months biologically older than non- or very light drinkers, and the least physically active were around 3 months biologically older than the most physically active. These associations suggest that a healthier lifestyle may slow biological aging, thereby reducing age-related disease risk. However, associations with BMI, alcohol use and smoking were inconsistent across clocks. Inconsistent or relatively small associations with risk factors have been reported for other first-generation clocks^21–23^ and may relate to differences in training population and how risk factors are distributed with age. For instance, BMI was positively associated with the Wang clock that was trained in middle-aged participants but negatively associated with the Sathyan clock that was trained in older participants. Changes in adiposity are a well-established part of the aging phenotype, with increases generally observed as people enter middle age, followed by decreases in late life^24^. Therefore, the Wang and Sathyan clocks probably capture the proteomic profile of greater and lower adiposity, respectively, in addition to biological age processes.

Other differences may also relate to how the clocks were trained. The Wang clock provided the best absolute prediction of chronological age among published clocks in the EPIC sample, which has a similar age distribution to the training population used by Wang. The Sathyan clock was trained in only older participants, some with comorbidities, and had the strongest associations with dementia and AD. The age-related changes captured by the Sathyan clock may be more relevant to these diseases, which have the oldest average age of disease onset. The Oh clock showed the strongest mortality prediction among published clocks, which may be due to the distinct machine learning algorithm employed, that combined models developed from bootstrapped resampling within the same population to improve generalizability. The Global clock takes a similar ensemble approach through averaging age predictions produced in different populations. We speculate that this may ameliorate the influence of training population characteristics, and potentially also statistical error. This is indicated by slightly larger associations with mortality in both the EPIC and Whitehall II studies, and more coherent associations with risk factors. As standardized proteomic data become more widely available around the world, future studies should train clocks in diverse populations, which may be further combined to provide biological age estimates less influenced by cohort effects particular to a single training population.

We observed a significant correlation between the strength of age gap associations and the incidence of diseases by age, derived from UK national electronic health records. This adds further support for both the use of proteomic clocks to assess biological age and to the Geroscience hypothesis that biological aging itself is a root cause of many age-related diseases. Significant associations were observed with diseases such as dementia, cardiovascular disease and some cancers that show a similar pattern of incidence, characterized by an exponential increase in disease onset with age. Associations were not significant for diseases with a more gradual relationship between disease onset and age, such as type 2 diabetes, or for those not clearly age-related, such as Hodgkin’s lymphoma. Argentieri et al. similarly reported associations between an Olink-based clock and several age-related diseases in UK Biobank, including stroke, heart disease and dementia and others not investigated here^7^. While they reported a significant association with type 2 diabetes, the effect size was among the smallest of all outcomes tested^7^. In the present study, stronger associations were observed for clocks more closely related to BMI such as the Wang and Oh clocks, suggesting that age-related increases in type 2 diabetes incidence may reflect rising adiposity, rather than direct effects of biological aging itself. Among cancers, the UK Biobank study also reported significant associations for esophageal and lung cancers, but unlike this study, significant associations were also observed for non-Hodgkin lymphoma and prostate cancer. They did not observe significant associations with breast, colorectal, ovarian and liver cancers^7^. In this study, which had almost twice as many cases as UK Biobank, we observed a highly significant association with liver cancer risk. Associations remained stable after exclusion of cases occurring within 5 years of proteomic age assessment.

Organ-specific clocks represent an advancement for biological age assessment as they recognize that organ systems age at different rates within individuals^25^. We observed some specificity in organ aging and disease, for kidney, lung and stomach cancers, but also generalized effects. Similar findings were recently reported in the UK Whitehall II cohort^15^. For instance, while heart and artery aging were among the strongest predictors of cardiovascular diseases, aging across multiple organs contributed to risk of these outcomes. Immune system aging predicted the largest number of diseases, emphasizing the central role of inflammatory processes in aging and chronic disease^26^. Despite a weak association with chronological age within the EPIC study, the kidney clock was associated with six health endpoints and clustered together with the immune, conventional and organismal clocks, suggesting it is also capturing more systematic ageing processes. Indeed, the globular filtration rate, a marker of kidney function, is recognized as an effective marker of functional aging^27^. Although brain age has been associated with AD in cross-sectional analysis^13^, the brain age gap did not predict risk of neurodegenerative outcomes here or in the Whitehall study over around 20 years of follow-up^15^. It is likely that, in mid-life, systematic aging is a stronger predictor of later brain aging and neurodegeneration. All organ age gaps were predictive of all-cause mortality, although the strongest associations were observed for conventional and organismal age gaps, which are measures of systematic aging. When combined into a single model, we found that the organismal, artery, immune, heart, kidney, lung and muscle age gaps all provided independent predictions of mortality.

Limitations of this study include the single baseline assessment of proteins, which provides only a snapshot of biological age, while the risk factor analysis was cross-sectional, using lifestyle information collected once at baseline. Exposure misclassification in risk factor assessments may have also contributed to some residual confounding. The sample is overwhelmingly of white ethnicity and, while reflective of the source population at the time of baseline assessment, has limited generalizability to other ethnicities. Genetic data were unavailable for all participants, preventing adjustment for genetic principal components to account for population structure. We note that all included clocks, including the Global clock, showed age dependency in prediction performance, and while this was corrected for when deriving the age gap metric, further work is required to improve absolute age prediction for these clocks. Despite observing robust associations with disease risk, an observational study, such as ours, cannot establish causality. However, the study has considerable strengths. Its large sample size, particularly in the context of proteomic studies, and the design as a population-based sample greatly increases its generalizability across the European population. The case–cohort approach provides an efficient use of study resources to assess many cases across multiple disease classes, increasing study power. Case ascertainment used multiple approaches reducing disease misclassification. Finally, the longitudinal design limits reverse causality and allows investigation of how ageing in midlife influences disease risk in later life.

Proteomic age clocks, based on blood-based technologies that measure thousands of proteins, may offer a promising approach to biologically informed risk stratification and improved prevention of age-related diseases and multimorbidity. Beyond traditional clinical markers focused on specific pathologies, biological age measures can provide complementary information relevant to personalized prevention strategies^28^. Other potential applications include their use as surrogate endpoints in clinical trials of healthy aging and drug development. However, although we observed a small but significant improvement in mortality prediction with the inclusion of proteomic age, randomized controlled trials in primary care are needed to determine whether integrating proteomic age into existing risk models enhances prediction, guides treatment and ultimately reduces disease incidence.

In this comprehensive study, we show that an accelerated aging phenotype captured by proteomic clocks is associated with increased risk of cardiovascular diseases, dementia, several age-related cancers and mortality. While we find that all conventional clocks trained in different populations showed similar patterns of association with mortality and disease risk, we show that a Global age clock, combining proteomic age estimates from these clocks, provides a slightly larger association with mortality. The Global age gap was sensitive to lifestyle risk factors including smoking, heavy alcohol use and physical inactivity and organ-specific proteomic clocks showed stronger prediction of kidney, lung and liver cancers. Future work should focus on validation in diverse populations and integration of genetic data to enhance mechanistic insight and causal inference. Overall, proteomic clocks hold strong potential for advancing understanding of the links between aging and health.

## Methods

### Study population

EPIC is a cohort composed of 521,32 participants (70.1% female) who were recruited between 1992 and 2000 across ten European countries^29^. A subset of these participants was selected into the EPIC-Somalogic study, which aims to discover biomarkers of cancer, diabetes, cardiovascular diseases, neurodegenerative diseases and mortality using the Somascan proteomics assay.

Participants from EPIC centers in Italy, Spain, the Netherlands, and the UK with available baseline plasma samples, baseline dietary and lifestyle data, and complete follow-up data, aged 35–75 years, were eligible for the study (Fig. 1). The study followed a case–cohort design, which is an efficient design for prospective evaluation of multiple endpoints^30^. Participants were selected into a main subcohort, previously used for the EPIC-InterAct and EPIC-Heart studies^31,32^. This main subcohort is a randomly selected, representative sample of participants in the participating EPIC centers, including incident disease cases and deaths. In addition, case sets were selected for incident disease events and deaths including cancers, cardiovascular diseases, type 2 diabetes and neurodegenerative disease (all-cause dementia, AD and PD). For the study of PD, additional samples were added to both the case and subcohort sample from Heidelberg study center in Germany (Fig. 1). For the AD and dementia case–cohort study, only Spanish centers contributed cases alongside noncases.

EPIC was approved by the Ethics Committee of the International Agency for Research on Cancer (IARC), Lyon, France, and local ethics committees of the study centers. All participants provided written informed consent for the collection, storage and individual follow-up of their data.

We replicated the analyses with all-cause mortality in the Whitehall II study, which included 10,308 British civil servants aged 35–55 years from 20 London-based departments at the first clinical examination (10 September 1985 to 29 March 1988^33^). Plasma samples for proteomic analyses were collected during a subsequent examination (24 April 1997 to 8 January 1999), when participants were aged 45–69 years, forming the baseline of the present analysis. The analytic sample with valid data on proteomic age clocks and linked mortality records comprised 6,240 participants. They were followed up for a median of 25.1 years through linked electronic health records from the National Health Services.

Ethical approval for the Whitehall II study was obtained from the University College London Medical School Committee on the Ethics of Human Research (85/0938) and the London–Harrow and Scotland A Research Ethics Committees on the Ethics of Human Research. Informed written consent was obtained from all participants.

### Case ascertainment

Incident disease cases, coded using the 10th revision of the World Health Organization (WHO) International Statistical Classification of Diseases (ICD-10), were identified using multiple sources of evidence including self-report, linkage to primary-care registers, secondary-care registers, medication use (drug registers), hospital admissions and mortality data^29,31,32^. Information from any follow-up visit or external evidence with a date later than the baseline visit was used. Incident neurodegenerative disease cases were first identified by record linkage with existing health databases and subsequently validated by re-review of medical records^34^. Data on total and cause-specific mortality were collected through mortality registries or active follow-up and death-record collection^29^

### Proteomic assessment

In EPIC, blood was collected in plasma citrate tubes at the baseline clinical assessment and processed into plasma and stored in liquid nitrogen at the IARC central biorepository, in Lyon, France. Selected samples were shipped to the SomaLogic laboratory in Boulder, CO, USA on dry ice.

Plasma samples were analyzed using the SomaScan ver 4.1 array, which quantifies approximately 7,000 proteins simultaneously^35^. In brief, plasma samples were incubated with a mixture of modified aptamers to generate aptamer–protein complexes, followed by several washing steps, elution of the fluorescently labeled aptamers from the target protein, and quantification on a DNA array (Agilent Technologies). Internal SomaLogic standardization and quality control (QC) procedures were then applied on the raw data, including hybridization normalization, intraplate median normalization, plate scaling and calibration using matrix-matched calibrator controls. Adaptive normalization by maximum likelihood was applied using internal reference sets, to improve comparability with external datasets. Standard QC checks against triplicates on each plate and predefined acceptance criteria were applied, leaving 17,841 samples with 7,285 aptamers (6,381 proteins) available for analysis.

Samples flagged by SomaLogic for failing to meet standard acceptance criteria for adaptive normalization by maximum likelihood were excluded (247 samples)^36^. In addition, we applied a further internal QC step to exclude 121 multivariate outliers, detected using unbiased principal component analysis projection based on local outlier factor^37^ and Tukey’s rule, using the bigutilr R package.

In the Whitehall II study, protein analyses used EDTA plasma samples measured in 1997–1999 and stored in 0.25-ml aliquots at −80 °C. As previously described, proteins were measured using SomaScan v.4.0 and v.4.1 assays^15^. Assays were validated against an external reference population, and protein-specific conversion coefficients were used to balance technical differences between versions 4.0 and 4.1. Standard SomaLogic normalization, calibration and QC were done on all samples. Scale factor acceptance criteria were passed for all plates for Whitehall EDTA samples. QC Percent in Tails refers to the percentage of SOMAmer reagents in the QC control that fall outside the accepted accuracy range (0.8–1.2) when compared with the reference. All plates in Whitehall study passed the acceptance criteria.

### Proteomic age calculation

Proteomic ages were calculated using published weights from five studies, including Tanaka et al.^10^, Lehallier et al.^16^, Sathyan et al.^11^, Oh et al.^13^ and Wang et al.^12^. We refer to these clocks as ‘conventional clocks’ as they were trained using all available aptamers and we refer to each clock by the first author of the original study. The Tanaka clock was trained from a set of around 1,300 proteins among healthy participants free from chronic disease and cognitive or functional impairment aged 22–93 years from two US Baltimore-based studies^10^. The Lehallier clock was trained from a set of around 3,000 proteins among participants aged 18–76 years in the UK INTERVAL study, combined with participants aged 61–95 years in the US Longevity study of Ashkenazi Jews, 50% of whom were offspring of long-lived parents^16^. The Sathyan clock was trained using around 5,000 proteins among the same older participants of the Longevity study, some of whom had comorbid condition presents as may be expected for their age range^11^. The Oh clock was also trained using around 5,000 proteins in cognitively healthy controls, aged 27–104 years in the US Knight-ADRC study^13^. Finally, the Wang clock was trained using around 5,000 proteins in the US multi-ethnic ARIC study in healthy participants aged 46–70 years free from major age-associated diseases^12^. For the Wang clock we used the version trained in midlife participants as closest to the age range of EPIC participants and to provide contrast with other clocks trained in older populations.

Before calculation, protein intensity values were ‘lifted’ to those provided with the v4 Somalogic assay using the multiplication scaling factors provided by Somlogic with the lift_adat function within the SomaDataIO R package. Proteomic data were transformed and/or scaled as specified in each publication, for appropriate clock calculation.

Proteomic clocks, including organismal and organ-specific proteomic clocks, were calculated using the Python organage package (https://github.com/hamiltonoh/organage) using the model weights provided by Oh et al.^13^. No other studies have currently trained organ-specific clocks on SomaScan data. The organ-specific clocks were trained using proteins putatively annotated to their organ sources, defined as a fourfold greater gene expression in a particular organ compared with any other organ, using the GTEX database^38^, as further described by Oh et al.^13^. In addition, an organismal clock was trained based using only proteins that were nonspecific to a particular organ^13^.

To maintain consistency with previous approaches, the ‘Age gap’ was calculated as described by Oh et al.^13^ by fitting a local regression, that allows for nonlinearity between predicted and chronological age at blood sampling using the lowess function in R for all clocks (with fraction parameter set to 2/3). Individual sample age gaps were then calculated as the difference between predicted age and the lowess regression estimate. The age gaps can be interpreted as the biological age component of proteomic predicted age and are expressed as years of higher or lower proteomic age relative to one chronological age.

In addition, we calculated a ‘Global’ clock age as the mean of the predicted ages from the Tanaka, Lehallier, Sathyan, Oh and Wang clocks. This Global age metric was used to assess absolute chronological age prediction. Similarly, a Global ‘age gap’ was calculated as the mean of these five conventional clock unstandardized age gaps. The age gaps were then *z*-scored for each published clock and the Global clock to account for differing variability, which were used as the primary exposure in all association analyses allowing direct comparison between the clocks

### Covariates

BMI (kg m^−^^2^) was derived from measured height and weight in all centers, except in Oxford, where it was self-reported^29^. A validated index capturing all physical activity domains (Cambridge Index) was computed from physical activity during recreational activities and at work^39^. Diet, including alcohol intake, was assessed using validated country- or center-specific dietary questionnaires designed to capture habitual consumption over the year preceding the study recruitment^29^. A healthy diet score was derived from six dietary factors as previously described^40^. Information on smoking status and education attainment was obtained using lifestyle questionnaires^29^. An overall HLI was determined by assigning scores of 0 to 4 to five lifestyle risk factors (BMI, smoking, alcohol use, diet and physical activity) for which a higher point value indicates a healthier behavior, as previously described^40^

### Statistics and reproducibility

Based on an available budget to measure proteomics in approximately 18,000 plasma samples, participants meeting eligibility criteria (complete baseline and follow-up information) from included EPIC centers (Italy, Spain, UK and the Netherlands) were randomly selected into a representative subcohort (around half of those selected into the InterAct subcohort^32^) for proteomic assessment. In addition, of cases not already included in the InterAct subcohort, approximately half of available incident deaths and cases of major cancers, cardiovascular diseases and type 2 diabetes were randomly selected into case sets. For the neurodegenerative disease arm of the study, all available cases outside the representative subcohort were selected from included EPIC centers, additionally including the Heidelberg center in Germany, and 200 randomly selected participants from Heidelberg were included in the subcohort. Statistical analysis was unblinded and no data points were excluded following the initial proteomic QC.

Using the case–cohort design, including the subcohort and the respective mortality or disease samples, we tested associations of baseline age-gap *z* scores against 25 incident disease and mortality endpoints. We fitted Cox proportional hazards regression models, using the survival R package with age as the underlying time scale, and stratified by sex, center and 5-year age groups to ensure proportionality of hazards and account for nonlinear age associations. Participants who died during follow-up were treated as censored in the incident disease analyses. In the EPIC study, to account for overrepresentation of cases compared with the original cohort, we applied Prentice weighting to cases outside the subcohort to provide unbiased inferences^41^. Prevalent disease cases had been excluded already, except for 150 diabetics, who were removed for the analysis of incident type 2 diabetes.

We present both base models (stratified by sex, center and 5-year age groups) and risk-adjusted models that additionally included smoking status (never/former/current), alcohol consumption (g per day), BMI (continuous score), healthy diet score (categorized into five quintiles), physical activity (metabolic equivalents of tasks, METs) and education level (none, primary school completed, technical/professional school, secondary school, and longer education (including university degree)). Covariates were chosen a priori based on their well-established associations with mortality risk and data availability across all participants. The same adjustment set was used across all outcomes to maintain comparability. To maintain sample size across base and risk factor-adjusted models, missing covariates (less than 6%) were imputed using a single imputation based on 50 iterations using the mice package in R for all samples. In addition to the covariates used in the risk factor models, covariates including sex, age, center, main subcohort membership, HLI and Mediterranean diet score were used as predictors in the imputation models.

For analyses of mortality in Whitehall II, base models were stratified by sex and 5-year age group. Risk factor-adjusted models were additionally adjusted for smoking status (never/former/current), alcohol consumption (g per day), BMI (continuous score), frequency of eating fruit and vegetables, physical activity level (meeting WHO recommended level or not) and education level (elementary or lower secondary, higher secondary, degree or higher).

Clustered heatmaps were drawn using the pheatmap package in R to display associations (log HRs) across clocks/diseases. Clustering was performed using complete linkage hierarchical clustering. To account for multiple testing across the clocks and disease endpoints, we applied a 5% FDR correction, using the Benjamini–Hochberg method (1995). Calculation of the FDR thresholds was conducted separately for the analysis of conventional and organ-specific clocks.

To biologically characterize the conventional and Global age clocks, we conducted functional enrichment analysis using the ClusterProfiler R package^42^. SomaScan SeqID for proteins present among clock predictors was mapped to EnrezID for input. We tested the overrepresentation of pathways in KEGG^43^, GO (molecular function, cellular component and biological process)^44^ and Reactome^45^ databases using hypergeometric tests against the whole gene sets available within each database. *P* values were corrected for multiple pathway comparisons via FDR separately for each database.

We extracted information on the relationship between disease incidence and chronological age from the study Kuan et al.^18^, which used the Clinical Practice Research Datalink, an electronic health record dataset from over 3 million individuals from across England. Specifically, for each disease we extracted the age-dependent component (*β*, the rate of disease onset increase over age) from Gompertz–Makeham models fitted by Kuan et al. A higher rate indicates a closer relationship between disease onset increase with age and implies the disease is more likely to be age-related. Across 22 diseases that could be matched across studies, we conducted Spearman’s nonparametric correlation to test the strength and significance of the relationship between the *β* coefficients from Kuan et al. with log hazards from our risk factor-adjusted models with the proteomic age-gap *z* scores. We also classified the diseases in EPIC according to the age clusters identified by Kuan et al. The diseases assessed in EPIC included those in clusters 1 and 3, for which disease onset increases exponentially with age, those in cluster 4, for which disease onset increases more gradually with age, and one disease (Hodgkin’s lymphoma) in age cluster 5, which is not considered age related at all. Clusters 1 and 3 are distinguished by the median age of disease onset of 82 years and 69 years, respectively.

In sensitivity analyses, to understand the potential impact of early pathology on age gap and disease and mortality associations, we tested associations in the risk factor-adjusted models between the Global proteomic age gap and disease endpoints, restricting to events that occurred only after 2 years and after 5 years since recruitment. To explore the impact of smoking on associations, we similarly restricted analyses to never smokers.

We examined associations between the quintiles of HLI and the five healthy life stye index components with the Global age-gap *z* score (as the dependent variable), using separate linear regressions in main subcohort, adjusted for sex, age and study center. All these risk factors were coded into five categories to aid comparison, using the least healthy category as the comparator.

We further examined the HLI as continuous score for all conventional clocks, adjusted for sex, age and study center. To examine the independent role of individual risk factors and examine consistency across clocks, we additionally fitted a linear model including sex, age, study center, smoking status (current smoker versus never/former smoker), alcohol consumption (g per day, scaled), BMI (continuous score, scaled), healthy diet score (continuous score, scaled), physical activity (METs, scaled) and education level (longer versus less education) for each conventional age-gap *z* score. Risk factor coefficients from this mutually adjusted model were presented on a heatmap.

We used lasso-penalized Cox regression to select organ-specific clocks for inclusion in a combined model of all-cause mortality, implemented using the glmnet R package. Fivefold cross-validation was performed to tune the lambda hyperparameter for optimum prediction. Selected organ age gaps were then used to fit a Cox model of mortality, including risk factors, stratified by sex, center and 5-year age groups and Prentice weighted as previously described.

To enhance clinical interpretation, the discriminatory power of different prediction models for were compared using Harrell’s concordance index in the EPIC study. All predictive Cox models included chronological age and sex as covariates, stratified by study center, and used follow-up time in years as the underlying time scale. We compared addition of risk factors only, Global age gap only, combined age gaps and risk factor models, and using all available organ-specific and organismal age gaps. The 95% CIs were calculated for all concordance indices using 500 bootstraps. We tested model improvement upon addition of the Global age gap to the risk factor model using the log-likelihood ratio test.

All analyses were performed in R ver. 4.3.1.

### Reporting summary

Further information on research design is available in the Nature Portfolio Reporting Summary linked to this article.

## Supplementary information

Supplementary Information

Reporting Summary

Peer Review File

## Source data

Source Data Fig. 2 Statistical source data.

Source Data Fig. 3 Statistical source data.

Source Data Fig. 4 Statistical source data.

Source Data Fig. 5 Statistical source data.

Source Data Fig. 6 Statistical source data.

Source Data Extended Data Fig. 1 Statistical source data.

Source Data Extended Data Fig. 2 Statistical source data.

Source Data Extended Data Fig. 3 Statistical source data.

Source Data Extended Data Fig. 4 Statistical source data.

Source Data Extended Data Fig. 5 Statistical source data.

Source Data Extended Data Fig. 6 Statistical source data.

Source Data Extended Data Fig. 7 Statistical source data.

Source Data Extended Data Fig. 8 Statistical source data.

Source Data Extended Data Fig. 9 Statistical source data.

Source Data Extended Data Fig. 10 Statistical source data.

## Data Availability

EPIC proteomic and other data can be accessed by external researchers through the IARC Scientific IT platform after submission of a research proposal and approval by the relevant EPIC working group and the EPIC steering committee. Full details of the access policy and procedures for data access are available at https://epic.iarc.fr/access/.

## Code availability

Statistical code for the analyses described here is available via GitHub at https://github.com/oliverrobinson2025/Global-proteomic-age.
