# Supplementary material for: Associations of proteomic age clocks with lifestyle risk factors, incident chronic diseases and mortality in two European cohorts
Source: Nat Aging. 2026 Jun 29;6(7):1437–51. doi: 10.1038/s43587-026-01163-6 (PMC13375651; doi:10.1038/s43587-026-01163-6)
Supplement: Supplementary file 1 — Supplementary Information [file 43587_2026_1163_MOESM1_ESM.pdf]

nature aging

Supplementary information https://doi.org/10.1038/s43587-026-01163-6

Associations of proteomic age clocks with lifestyle risk factors, incident chronic diseases and mortality in two European cohorts

Supplementary File

Contents

**Figure/table**	**Page Number**
Figure S1: Enrichment of Gene Ontology terms among proteins contained in the conventional clocks and the Global age clock.	2
Figure S2: Hazard ratios per classical proteomic age gap year with all -cause mortality.	3
Figure S3: Chronological age prediction in the Whitehall II study.	4
Figure S4: Comparison of discriminatory power showing concordance index for various models for prediction of mortality.	5
Table S1: Characteristics of the EPIC Main Subcohort	6
Table S2: Characteristics of the mortality case sample.	9
Table S3: Characteristics of the cardiovascular disease case sample.	11
Table S4: Characteristics of the type 2 diabetes case sample.	13
Table S5: Characteristics of the cancer case sample.	15
Table S6: Characteristics of the subcohort selected as comparator for the Dementia/ Alzheimer's disease case-cohort analysis.	19
Table S7: Characteristics of the dementia case sample.	21
Table S8: Characteristics of the subcohort selected as comparator for the Parkinsons's disease case-cohort analysis.	24
Table S9: Characteristics of the Parkinson's disease case sample.	27

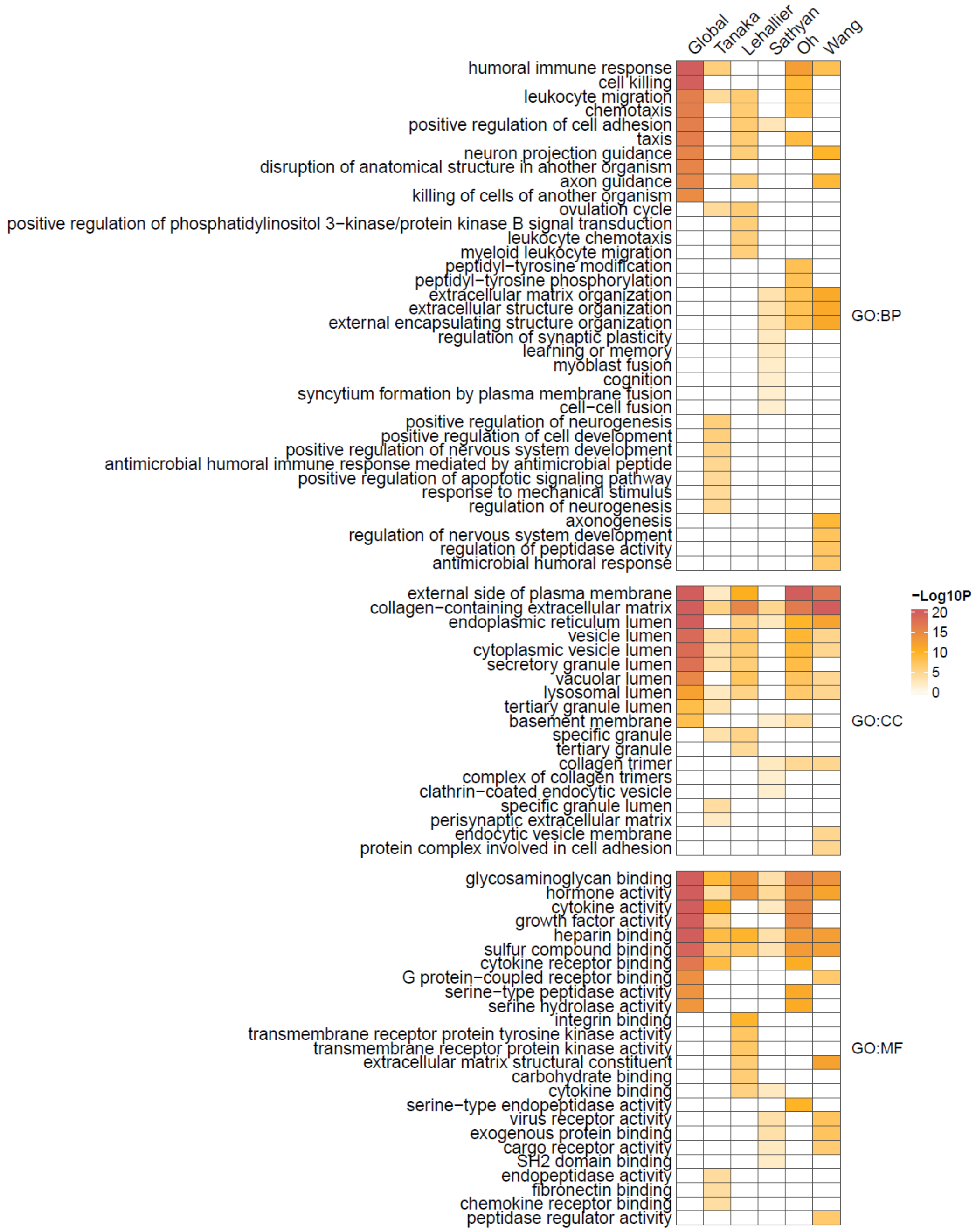

Figure S1: Enrichment of Gene Ontology terms among proteins contained in the conventional clocks and the Global age clock. Pathway enrichment was tested using a two-sided Fisher's exact test and adjusted for multiple comparisons using false discovery rate (FDR) correction.The top 10 pathways passing FDR correction for each clock are shown. MF: Molecular Function. CC: Cellular Component. BP: Biological Process.

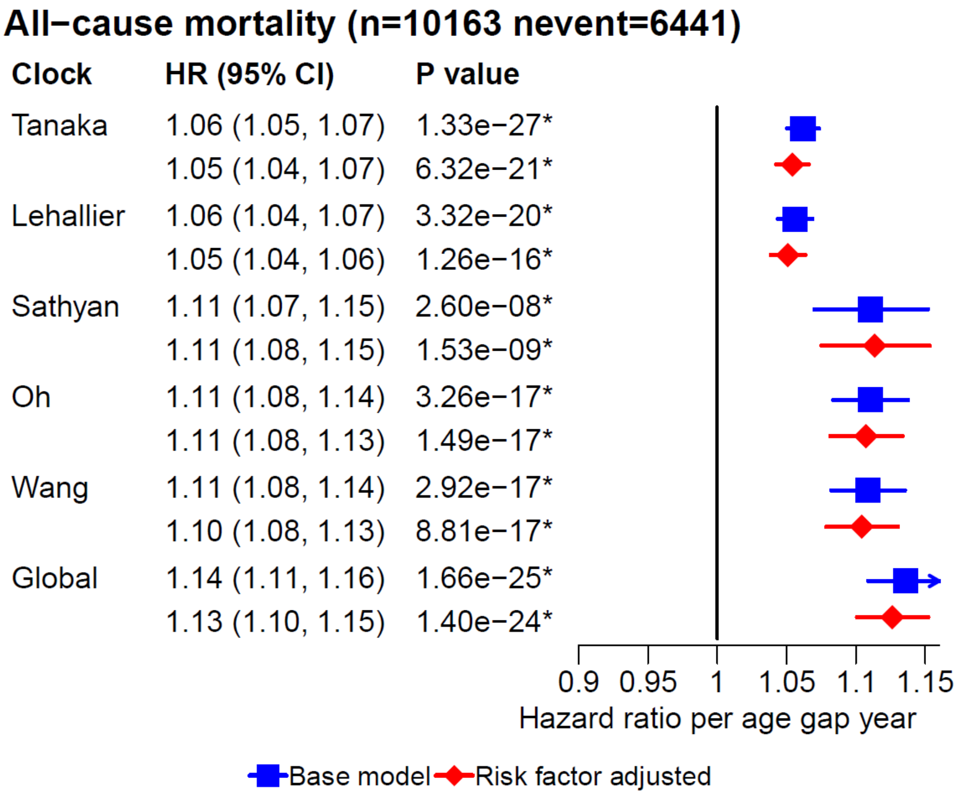

Figure S2: Hazard ratios per classical proteomic age gap year with all -cause mortality. All *Cox proportion hazards regression models were* stratified by study centre, sex, and five-year age group. Risk factor adjusted model additionally adjusted for education level, smoking status, alcohol consumption, BMI, healthy diet score and physical activity. The centre of each point represents the estimated hazard ratio per 1-SD increase in proteomic age gap z-score, and the error bars represent 95% confidence intervals.

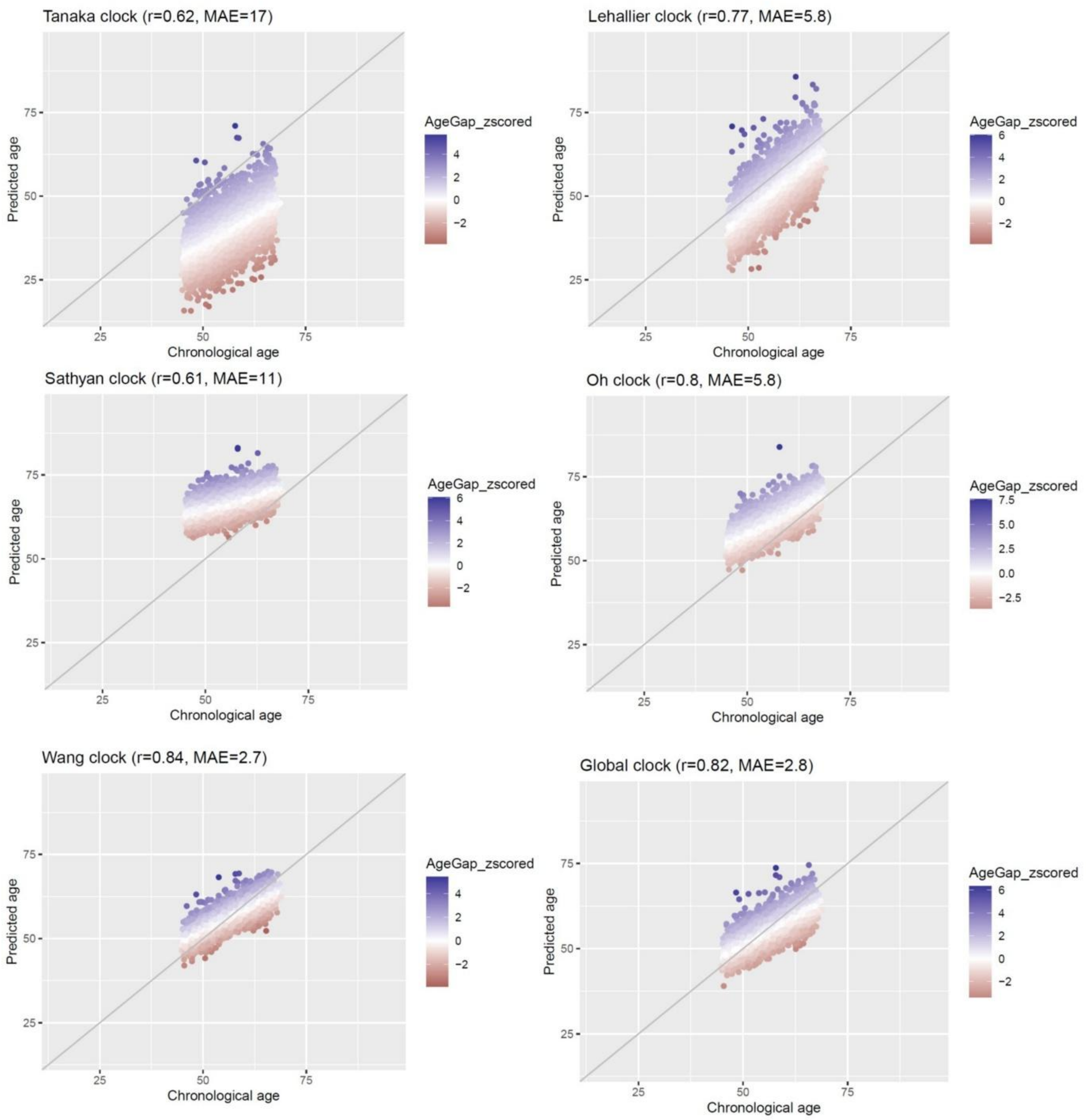

Figure S3: Chronological age prediction in the Whitehall II study. Scatterplots of predicted versus chronological age for all clocks used, showing Pearson's correlations and mean absolute error (MAE).

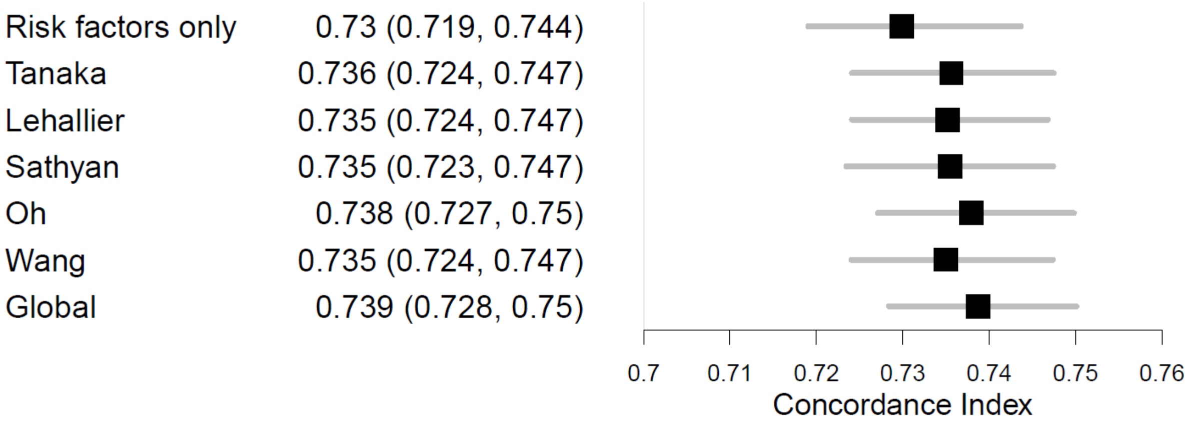

Figure S4: Comparison of discriminatory power showing concordance index for various models for prediction of mortality. All models include age, sex, smoking education level, smoking status, alcohol consumption, BMI, healthy diet score and physical activity and stratified by study centre. All error bars show 95% confidence intervals.

35 **Table S1: Characteristics of the EPIC Main Subcohort*.**

	Italy	Spain	United Kingdom	The Netherlands	Overall
	(N=982)	(N=2066)	(N=489)	(N=578)	(N=4115)
**Age (years)**	50.7 (7.79)	49.4 (7.93)	57.2 (9.61)	55.3 (8.21)	51.4 (8.64)
**sex**					
**Male**	377 (38.4%)	848 (41.0%)	246 (50.3%)	102 (17.6%)	1573 (38.2%)
**Female**	605 (61.6%)	1218 (59.0%)	243 (49.7%)	476 (82.4%)	2542 (61.8%)
**Level of Education**					
**None**	9 (0.9%)	689 (33.3%)	0 (0%)	0 (0%)	698 (17.0%)
**Primary school completed**	470 (47.9%)	811 (39.3%)	135 (27.6%)	121 (20.9%)	1537 (37.4%)
**Technical/professional school**	128 (13.0%)	182 (8.8%)	146 (29.9%)	165 (28.5%)	621 (15.1%)
**Secondary school**	246 (25.1%)	133 (6.4%)	47 (9.6%)	168 (29.1%)	594 (14.4%)
**Longer education (incl. University deg.)**	127 (12.9%)	234 (11.3%)	101 (20.7%)	124 (21.5%)	586 (14.2%)
**Missing**	2 (0.2%)	17 (0.8%)	60 (12.3%)	0 (0%)	79 (1.9%)
**BMI (kg/m2)**	25.7 (4.02)	28.3 (4.37)	25.4 (3.68)	25.5 (3.73)	26.9 (4.35)
**Alcohol consumption (grams/day)**	14.5 (19.4)	14.5 (22.7)	8.04 (10.8)	9.89 (14.2)	13.1 (19.9)
**Smoking Status**					
**Never**	461 (46.9%)	1136 (55.0%)	244 (49.9%)	256 (44.3%)	2097 (51.0%)
**Former**	271 (27.6%)	364 (17.6%)	169 (34.6%)	180 (31.1%)	984 (23.9%)
**Smoker**	250 (25.5%)	566 (27.4%)	76 (15.5%)	142 (24.6%)	1034 (25.1%)
**Physical activity (METS recreational and household activity)**	90.8 (59.4)	94.3 (56.0)	90.2 (51.0)	117 (51.6)	96.0 (56.3)
**Missing**	1 (0.1%)	0 (0%)	5 (1.0%)	32 (5.5%)	38 (0.9%)
**Cambridge physical activity index**					
**Inactive**	237 (24.1%)	757 (36.6%)	140 (28.6%)	42 (7.3%)	1176 (28.6%)
**Moderately inactive**	400 (40.7%)	670 (32.4%)	161 (32.9%)	137 (23.7%)	1368 (33.2%)
**Moderately active**	190 (19.3%)	375 (18.2%)	101 (20.7%)	127 (22.0%)	793 (19.3%)
**Active**	154 (15.7%)	264 (12.8%)	82 (16.8%)	240 (41.5%)	740 (18.0%)
**Missing**	1 (0.1%)	0 (0%)	5 (1.0%)	32 (5.5%)	38 (0.9%)

**Healthy score for diet (range 0-54)**	27.1 (6.61)	30.3 (5.94)	24.6 (6.73)	27.2 (6.41)	28.4 (6.59)
**Missing**	20 (2.0%)	42 (2.0%)	9 (1.8%)	11 (1.9%)	82 (2.0%)
**Healthy lifestyle index (range 0 - 20)**	11.2 (3.37)	11.9 (3.45)	12.2 (3.00)	12.7 (3.09)	11.9 (3.37)
**Missing**	21 (2.1%)	42 (2.0%)	14 (2.9%)	43 (7.4%)	120 (2.9%)
**InterAct prevalent diabetes reported**					
**No**	957 (97.5%)	1927 (93.3%)	476 (97.3%)	561 (97.1%)	3921 (95.3%)
**Yes**	15 (1.5%)	109 (5.3%)	13 (2.7%)	13 (2.2%)	150 (3.6%)
**Missing**	10 (1.0%)	30 (1.5%)	0 (0%)	4 (0.7%)	44 (1.1%)
**Prevalent MI/Stroke reported**					
**No**	982 (100%)	2066 (100%)	489 (100%)	578 (100%)	4115 (100%)
**Yes**	0 (0%)	0 (0%)	0 (0%)	0 (0%)	0 (0%)
**Mortality status**					
**Non-case**	938 (95.5%)	1897 (91.8%)	376 (76.9%)	511 (88.4%)	3722 (90.4%)
**Incident**	44 (4.5%)	169 (8.2%)	113 (23.1%)	67 (11.6%)	393 (9.6%)
**Mortality follow up time (years)**	15.8 (2.65)	18.5 (2.36)	16.7 (3.46)	16.7 (2.56)	17.4 (2.85)
**Any first CVD event (CHD/Stroke) (fatal/non-fatal)**					
**Non-case**	960 (97.8%)	1975 (95.6%)	443 (90.6%)	545 (94.3%)	3923 (95.3%)
**Incident**	22 (2.2%)	91 (4.4%)	46 (9.4%)	33 (5.7%)	192 (4.7%)
**Cardiovascular disease follow up time (years)**	10.5 (2.42)	14.0 (2.15)	10.7 (2.14)	11.9 (2.12)	12.5 (2.72)
**Type 2 diabetes status**					
**Non-case**	947 (96.4%)	1893 (91.6%)	483 (98.8%)	556 (96.2%)	3879 (94.3%)
**Incident**	25 (2.5%)	143 (6.9%)	6 (1.2%)	18 (3.1%)	192 (4.7%)
**Missing**	10 (1.0%)	30 (1.5%)	0 (0%)	4 (0.7%)	44 (1.1%)
**Type 2 diabetes follow up time (years)**	10.7 (1.93)	12.9 (2.06)	11.1 (1.81)	12.0 (2.00)	12.0 (2.21)
**Missing**	10 (1.0%)	30 (1.5%)	0 (0%)	4 (0.7%)	44 (1.1%)
**Any malignant cancer**					
**Non-case**	884 (90.0%)	1876 (90.8%)	424 (86.7%)	530 (91.7%)	3714 (90.3%)
**Incident**	98 (10.0%)	190 (9.2%)	65 (13.3%)	48 (8.3%)	401 (9.7%)
**Cancer follow up time (years)**	14.2 (3.00)	16.1 (2.72)	14.7 (3.74)	14.5 (2.82)	15.2 (3.06)
**Neurodegenerative disease event**					

**Non-case**	972 (99.0%)	1962 (95.0%)	484 (99.0%)	571 (98.8%)	3989 (96.9%)
**Incident**	10 (1.0%)	104 (5.0%)	5 (1.0%)	7 (1.2%)	126 (3.1%)

**Subcohort used as comparator in case-cohort analyses for mortality, cancers, type 2 diabetes and cardiovascular diseases*

Continuous variables presented as mean (standard deviation)

Table S2: Characteristics of the mortality case sample.

	Italy	Spain	United Kingdom	The Netherlands	Overall
	(N=1104)	(N=1521)	(N=2161)	(N=1262)	(N=6048)
**Age (years)**	55.8 (7.65)	55.5 (7.65)	64.8 (8.04)	59.3 (7.38)	59.7 (8.75)
**sex**					
**Male**	498 (45.1%)	911 (59.9%)	1251 (57.9%)	277 (21.9%)	2937 (48.6%)
**Female**	606 (54.9%)	610 (40.1%)	910 (42.1%)	985 (78.1%)	3111 (51.4%)
**Level of Education**					
**None**	40 (3.6%)	650 (42.7%)	0 (0%)	0 (0%)	690 (11.4%)
**Primary school completed**	620 (56.2%)	535 (35.2%)	733 (33.9%)	377 (29.9%)	2265 (37.5%)
**Technical/professional school**	114 (10.3%)	110 (7.2%)	602 (27.9%)	395 (31.3%)	1221 (20.2%)
**Secondary school**	218 (19.7%)	91 (6.0%)	165 (7.6%)	325 (25.8%)	799 (13.2%)
**Longer education (incl. University deg.)**	110 (10.0%)	126 (8.3%)	278 (12.9%)	162 (12.8%)	676 (11.2%)
**Missing**	2 (0.2%)	9 (0.6%)	383 (17.7%)	3 (0.2%)	397 (6.6%)
**BMI (kg/m2)**	27.0 (4.67)	29.4 (4.60)	26.2 (4.23)	26.1 (4.28)	27.1 (4.62)
**Alcohol consumption (grams/day)**	16.4 (20.8)	21.0 (30.6)	8.45 (13.0)	10.7 (18.0)	13.5 (21.6)
**Smoking Status**					
**Never**	418 (37.9%)	659 (43.3%)	814 (37.7%)	421 (33.4%)	2312 (38.2%)
**Former**	296 (26.8%)	275 (18.1%)	980 (45.3%)	370 (29.3%)	1921 (31.8%)
**Smoker**	390 (35.3%)	587 (38.6%)	367 (17.0%)	471 (37.3%)	1815 (30.0%)
**Physical activity (METS recreational and household activity)**	84.4 (59.1)	80.1 (54.0)	87.6 (48.6)	111 (54.6)	89.7 (54.4)
**Missing**	0 (0%)	0 (0%)	13 (0.6%)	81 (6.4%)	94 (1.6%)
**Cambridge physical activity index**					
**Inactive**	392 (35.5%)	554 (36.4%)	1057 (48.9%)	165 (13.1%)	2168 (35.8%)
**Moderately inactive**	409 (37.0%)	538 (35.4%)	586 (27.1%)	333 (26.4%)	1866 (30.9%)
**Moderately active**	164 (14.9%)	252 (16.6%)	312 (14.4%)	279 (22.1%)	1007 (16.7%)
**Active**	139 (12.6%)	177 (11.6%)	193 (8.9%)	404 (32.0%)	913 (15.1%)

Missing	0 (0%)	0 (0%)	13 (0.6%)	81 (6.4%)	94 (1.6%)
**Healthy score for diet (range 0-54)**	27.4 (7.05)	30.5 (6.16)	23.8 (6.72)	25.7 (6.35)	26.5 (7.08)
Missing	27 (2.4%)	48 (3.2%)	43 (2.0%)	29 (2.3%)	147 (2.4%)
**Healthy lifestyle index (range 0 - 20)**	10.5 (3.54)	10.7 (3.64)	11.7 (2.97)	11.7 (3.08)	11.2 (3.32)
Missing	27 (2.4%)	48 (3.2%)	55 (2.5%)	108 (8.6%)	238 (3.9%)
**InterAct prevalent diabetes reported**					
No	960 (87.0%)	1320 (86.8%)	1849 (85.6%)	1171 (92.8%)	5300 (87.6%)
Yes	68 (6.2%)	154 (10.1%)	117 (5.4%)	67 (5.3%)	406 (6.7%)
Missing	76 (6.9%)	47 (3.1%)	195 (9.0%)	24 (1.9%)	342 (5.7%)
**Prevalent MI/Stroke reported**					
No	1104 (100%)	1521 (100%)	2161 (100%)	1262 (100%)	6048 (100%)
Yes	0 (0%)	0 (0%)	0 (0%)	0 (0%)	0 (0%)
**Mortality status**					
Non-case	0 (0%)	0 (0%)	0 (0%)	0 (0%)	0 (0%)
Incident	0 (0%)	0 (0%)	0 (0%)	0 (0%)	0 (0%)
**Mortality follow up time (years)**	1104 (100%)	1521 (100%)	2161 (100%)	1262 (100%)	6048 (100%)
**Follow up time deaths (yrs)**	10.4 (4.43)	12.8 (5.03)	11.7 (4.91)	11.7 (4.59)	11.8 (4.86)

Continuous variables presented as mean (standard deviation)

Table S3: Characteristics of the cardiovascular disease case sample.

	Italy	Spain	United Kingdom	The Netherlands	Overall
	(N=333)	(N=591)	(N=788)	(N=496)	(N=2208)
**Age (years)**	55.6 (7.22)	55.4 (7.36)	64.1 (8.15)	58.2 (7.48)	59.2 (8.56)
**sex**					
**Male**	171 (51.4%)	396 (67.0%)	505 (64.1%)	154 (31.0%)	1226 (55.5%)
**Female**	162 (48.6%)	195 (33.0%)	283 (35.9%)	342 (69.0%)	982 (44.5%)
**Level of Education**					
**None**	11 (3.3%)	248 (42.0%)	0 (0%)	0 (0%)	259 (11.7%)
**Primary school completed**	190 (57.1%)	218 (36.9%)	265 (33.6%)	136 (27.4%)	809 (36.6%)
**Technical/professional school**	39 (11.7%)	46 (7.8%)	228 (28.9%)	175 (35.3%)	488 (22.1%)
**Secondary school**	54 (16.2%)	30 (5.1%)	75 (9.5%)	124 (25.0%)	283 (12.8%)
**Longer education (incl. University deg.)**	39 (11.7%)	44 (7.4%)	87 (11.0%)	59 (11.9%)	229 (10.4%)
**Missing**	0 (0%)	5 (0.8%)	133 (16.9%)	2 (0.4%)	140 (6.3%)
**BMI (kg/m2)**	27.1 (4.06)	29.3 (3.83)	26.6 (4.00)	26.2 (4.09)	27.3 (4.18)
**Alcohol consumption (grams/day)**	18.2 (22.0)	21.1 (29.0)	8.06 (13.7)	11.3 (18.5)	13.8 (21.7)
**Smoking Status**					
**Never**	123 (36.9%)	257 (43.5%)	287 (36.4%)	149 (30.0%)	816 (37.0%)
**Former**	81 (24.3%)	104 (17.6%)	369 (46.8%)	163 (32.9%)	717 (32.5%)
**Smoker**	129 (38.7%)	230 (38.9%)	132 (16.8%)	184 (37.1%)	675 (30.6%)
**Physical activity (METS recreational and household activity)**	87.2 (60.5)	75.0 (54.3)	85.8 (49.7)	109 (53.3)	88.1 (54.8)
**Missing**	0 (0%)	0 (0%)	5 (0.6%)	39 (7.9%)	44 (2.0%)
**Cambridge physical activity index**					
**Inactive**	98 (29.4%)	205 (34.7%)	404 (51.3%)	61 (12.3%)	768 (34.8%)
**Moderately inactive**	124 (37.2%)	203 (34.3%)	185 (23.5%)	139 (28.0%)	651 (29.5%)
**Moderately active**	63 (18.9%)	112 (19.0%)	118 (15.0%)	103 (20.8%)	396 (17.9%)
**Active**	48 (14.4%)	71 (12.0%)	76 (9.6%)	154 (31.0%)	349 (15.8%)
**Missing**	0 (0%)	0 (0%)	5 (0.6%)	39 (7.9%)	44 (2.0%)
**Healthy score for diet (range 0-54)**	27.9 (7.06)	30.8 (6.06)	23.3 (6.25)	25.9 (6.46)	26.6 (7.03)

Missing	14 (4.2%)	21 (3.6%)	19 (2.4%)	15 (3.0%)	69 (3.1%)
**Healthy lifestyle index (range 0 - 20)**	10.4 (3.53)	10.5 (3.47)	11.5 (2.89)	11.6 (3.04)	11.1 (3.23)
Missing	14 (4.2%)	21 (3.6%)	24 (3.0%)	51 (10.3%)	110 (5.0%)
**InterAct prevalent diabetes reported**					
No	282 (84.7%)	497 (84.1%)	655 (83.1%)	462 (93.1%)	1896 (85.9%)
Yes	31 (9.3%)	78 (13.2%)	68 (8.6%)	28 (5.6%)	205 (9.3%)
Missing	20 (6.0%)	16 (2.7%)	65 (8.2%)	6 (1.2%)	107 (4.8%)
**Prevalent MI/Stroke reported**					
No	333 (100%)	591 (100%)	788 (100%)	496 (100%)	2208 (100%)
Yes	0 (0%)	0 (0%)	0 (0%)	0 (0%)	0 (0%)
**Any first Coronary Heart Disease event (I20-I25) (fatal/non-fatal)**					
Non-case	124 (37.2%)	261 (44.2%)	145 (18.4%)	147 (29.6%)	677 (30.7%)
Incident	209 (62.8%)	330 (55.8%)	643 (81.6%)	349 (70.4%)	1531 (69.3%)
**Coronary Heart Disease follow up time (years)**	7.74 (3.67)	10.6 (4.36)	7.19 (3.18)	8.15 (3.91)	8.40 (4.01)
**Any first stroke event (I60,I61,I63,I64) (fatal/non-fatal)**					
Non-case	199 (59.8%)	305 (51.6%)	598 (75.9%)	317 (63.9%)	1419 (64.3%)
Incident	134 (40.2%)	286 (48.4%)	190 (24.1%)	179 (36.1%)	789 (35.7%)
**Stroke follow up time (years)**	9.24 (3.70)	11.2 (4.30)	9.06 (3.23)	9.99 (3.66)	9.88 (3.81)

Continuous variables presented as mean (standard deviation)

Table S4: Characteristics of the type 2 diabetes case sample.

	Italy	Spain	United.Kingdom	The.Netherlands	Overall
	(N=253)	(N=580)	(N=157)	(N=154)	(N=1144)
**Age (years)**	54.2 (7.30)	53.9 (7.23)	60.9 (8.31)	58.6 (6.65)	55.6 (7.77)
**sex**					
**Male**	120 (47.4%)	355 (61.2%)	100 (63.7%)	34 (22.1%)	609 (53.2%)
**Female**	133 (52.6%)	225 (38.8%)	57 (36.3%)	120 (77.9%)	535 (46.8%)
**Level of Education**					
**None**	9 (3.6%)	271 (46.7%)	0 (0%)	0 (0%)	280 (24.5%)
**Primary school completed**	154 (60.9%)	213 (36.7%)	56 (35.7%)	52 (33.8%)	475 (41.5%)
**Technical/professional school**	22 (8.7%)	32 (5.5%)	47 (29.9%)	58 (37.7%)	159 (13.9%)
**Secondary school**	46 (18.2%)	32 (5.5%)	10 (6.4%)	32 (20.8%)	120 (10.5%)
**Longer education (incl. University deg.)**	22 (8.7%)	26 (4.5%)	23 (14.6%)	11 (7.1%)	82 (7.2%)
**Missing**	0 (0%)	6 (1.0%)	21 (13.4%)	1 (0.6%)	28 (2.4%)
**BMI (kg/m2)**	29.9 (4.92)	31.2 (4.45)	29.0 (5.19)	29.6 (4.56)	30.4 (4.75)
**Alcohol consumption (grams/day)**	12.4 (17.2)	21.7 (32.7)	9.86 (16.2)	7.89 (12.9)	16.1 (26.4)
**Smoking Status**					
**Never**	102 (40.3%)	267 (46.0%)	58 (36.9%)	66 (42.9%)	493 (43.1%)
**Former**	68 (26.9%)	106 (18.3%)	78 (49.7%)	50 (32.5%)	302 (26.4%)
**Smoker**	83 (32.8%)	207 (35.7%)	21 (13.4%)	38 (24.7%)	349 (30.5%)
**Physical activity (METS recreational and household activity)**	84.4 (60.1)	81.1 (55.1)	80.3 (45.7)	113 (56.8)	85.7 (56.2)
**Missing**	0 (0%)	0 (0%)	1 (0.6%)	13 (8.4%)	14 (1.2%)
**Cambridge physical activity index**					
**Inactive**	99 (39.1%)	216 (37.2%)	77 (49.0%)	29 (18.8%)	421 (36.8%)
**Moderately inactive**	87 (34.4%)	187 (32.2%)	40 (25.5%)	37 (24.0%)	351 (30.7%)
**Moderately active**	33 (13.0%)	106 (18.3%)	18 (11.5%)	32 (20.8%)	189 (16.5%)
**Active**	34 (13.4%)	71 (12.2%)	21 (13.4%)	43 (27.9%)	169 (14.8%)
**Missing**	0 (0%)	0 (0%)	1 (0.6%)	13 (8.4%)	14 (1.2%)
**Healthy score for diet (range 0-54)**	28.1 (7.07)	31.2 (6.05)	23.2 (6.82)	25.4 (6.11)	28.7 (7.05)
**Missing**	4 (1.6%)	10 (1.7%)	4 (2.5%)	5 (3.2%)	23 (2.0%)

**Healthy lifestyle index (range 0 - 20)**	10.2 (3.18)	10.6 (3.64)	10.5 (2.79)	11.1 (3.15)	10.6 (3.38)
**Missing**	4 (1.6%)	10 (1.7%)	5 (3.2%)	17 (11.0%)	36 (3.1%)
**InterAct prevalent diabetes reported**					
**No**	253 (100%)	580 (100%)	157 (100%)	154 (100%)	1144 (100%)
**Yes**	0 (0%)	0 (0%)	0 (0%)	0 (0%)	0 (0%)
**Prevalent MI/Stroke reported**					
**No**	253 (100%)	580 (100%)	157 (100%)	154 (100%)	1144 (100%)
**Yes**	0 (0%)	0 (0%)	0 (0%)	0 (0%)	0 (0%)
**Type 2 diabetes follow up time (years)**	6.50 (2.86)	8.13 (3.09)	6.28 (2.67)	5.36 (2.83)	7.14 (3.13)

Continuous variables presented as mean (standard deviation)

Table S5: Characteristics of the cancer case sample.

	Italy	Spain	United Kingdom	The Netherlands	Overall
	(N=1935)	(N=1602)	(N=1483)	(N=1125)	(N=6145)
**Age (years)**	53.9 (7.51)	53.1 (7.78)	60.6 (8.99)	56.8 (7.07)	55.8 (8.42)
**sex**					
**Male**	760 (39.3%)	946 (59.1%)	864 (58.3%)	256 (22.8%)	2826 (46.0%)
**Female**	1175 (60.7%)	656 (40.9%)	619 (41.7%)	869 (77.2%)	3319 (54.0%)
**Level of Education**					
**None**	37 (1.9%)	597 (37.3%)	0 (0%)	0 (0%)	634 (10.3%)
**Primary school completed**	1043 (53.9%)	615 (38.4%)	352 (23.7%)	262 (23.3%)	2272 (37.0%)
**Technical/professional school**	213 (11.0%)	122 (7.6%)	446 (30.1%)	368 (32.7%)	1149 (18.7%)
**Secondary school**	425 (22.0%)	108 (6.7%)	136 (9.2%)	299 (26.6%)	968 (15.8%)
**Longer education (incl. University deg.)**	214 (11.1%)	147 (9.2%)	269 (18.1%)	195 (17.3%)	825 (13.4%)
**Missing**	3 (0.2%)	13 (0.8%)	280 (18.9%)	1 (0.1%)	297 (4.8%)
**BMI (kg/m2)**	26.4 (4.18)	28.8 (4.25)	26.1 (3.88)	26.0 (4.02)	26.9 (4.26)
**Alcohol consumption (grams/day)**	15.4 (19.1)	20.2 (28.6)	8.89 (12.3)	11.8 (15.8)	14.4 (20.7)
**Smoking Status**					
**Never**	852 (44.0%)	719 (44.9%)	614 (41.4%)	403 (35.8%)	2588 (42.1%)
**Former**	525 (27.1%)	313 (19.5%)	626 (42.2%)	374 (33.2%)	1838 (29.9%)
**Smoker**	558 (28.8%)	570 (35.6%)	243 (16.4%)	348 (30.9%)	1719 (28.0%)
**Physical activity (METS recreational and household activity)**	89.8 (59.2)	82.3 (54.3)	88.3 (47.8)	115 (55.8)	91.8 (55.8)
**Missing**	0 (0%)	0 (0%)	9 (0.6%)	83 (7.4%)	92 (1.5%)
**Cambridge physical activity index**					
**Inactive**	605 (31.3%)	566 (35.3%)	565 (38.1%)	106 (9.4%)	1842 (30.0%)
**Moderately inactive**	732 (37.8%)	528 (33.0%)	438 (29.5%)	281 (25.0%)	1979 (32.2%)
**Moderately active**	312 (16.1%)	325 (20.3%)	287 (19.4%)	260 (23.1%)	1184 (19.3%)
**Active**	286 (14.8%)	183 (11.4%)	184 (12.4%)	395 (35.1%)	1048 (17.1%)
**Missing**	0 (0%)	0 (0%)	9 (0.6%)	83 (7.4%)	92 (1.5%)

**Healthy score for diet (range 0-54)**	27.0 (6.92)	30.4 (6.02)	24.2 (6.68)	26.3 (6.35)	27.1 (6.90)
**Missing**	45 (2.3%)	37 (2.3%)	25 (1.7%)	15 (1.3%)	122 (2.0%)
**Healthy lifestyle index (range 0 - 20)**	10.8 (3.45)	10.9 (3.56)	11.8 (3.01)	11.9 (3.03)	11.3 (3.34)
**Missing**	45 (2.3%)	37 (2.3%)	33 (2.2%)	98 (8.7%)	213 (3.5%)
**InterAct prevalent diabetes reported**					
**No**	1820 (94.1%)	1473 (91.9%)	1291 (87.1%)	1078 (95.8%)	5662 (92.1%)
**Yes**	63 (3.3%)	103 (6.4%)	37 (2.5%)	35 (3.1%)	238 (3.9%)
**Missing**	52 (2.7%)	26 (1.6%)	155 (10.5%)	12 (1.1%)	245 (4.0%)
**Prevalent MI/Stroke reported**					
**No**	1935 (100%)	1602 (100%)	1483 (100%)	1125 (100%)	6145 (100%)
**Yes**	0 (0%)	0 (0%)	0 (0%)	0 (0%)	0 (0%)
**Any malignant cancer**					
**Non-case**	0 (0%)	0 (0%)	0 (0%)	0 (0%)	0 (0%)
**Incident**	1935 (100%)	1602 (100%)	1483 (100%)	1125 (100%)	6145 (100%)
**Bladder malignant cancer**					
**Non-case**	1874 (96.8%)	1506 (94.0%)	1429 (96.4%)	1102 (98.0%)	5911 (96.2%)
**Incident**	61 (3.2%)	96 (6.0%)	54 (3.6%)	23 (2.0%)	234 (3.8%)
**Breast malignant cancer**					
**Non-case**	1516 (78.3%)	1420 (88.6%)	1404 (94.7%)	887 (78.8%)	5227 (85.1%)
**Incident**	419 (21.7%)	182 (11.4%)	79 (5.3%)	238 (21.2%)	918 (14.9%)
**Peri-menopausal (at blood coll.) breast cancer**					
**Non-case**	1850 (95.6%)	1586 (99.0%)	1461 (98.5%)	1071 (95.2%)	5968 (97.1%)
**Incident**	85 (4.4%)	16 (1.0%)	22 (1.5%)	54 (4.8%)	177 (2.9%)
**Post-menopausal (at blood coll.) breast cancer**					
**Non-case**	1743 (90.1%)	1534 (95.8%)	1450 (97.8%)	986 (87.6%)	5713 (93.0%)
**Incident**	192 (9.9%)	68 (4.2%)	33 (2.2%)	139 (12.4%)	432 (7.0%)
**Pre-menopausal (at blood coll.) breast cancer**					
**Non-case**	1793 (92.7%)	1504 (93.9%)	1459 (98.4%)	1080 (96.0%)	5836 (95.0%)
**Incident**	142 (7.3%)	98 (6.1%)	24 (1.6%)	45 (4.0%)	309 (5.0%)
**Colon-rectum malignant cancer**					

Non-case	1672 (86.4%)	1354 (84.5%)	1242 (83.7%)	936 (83.2%)	5204 (84.7%)
Incident	263 (13.6%)	248 (15.5%)	241 (16.3%)	189 (16.8%)	941 (15.3%)
Colon cancer					
Non-case	1745 (90.2%)	1454 (90.8%)	1325 (89.3%)	995 (88.4%)	5519 (89.8%)
Incident	190 (9.8%)	148 (9.2%)	158 (10.7%)	130 (11.6%)	626 (10.2%)
Rectum cancer					
Non-case	1862 (96.2%)	1502 (93.8%)	1400 (94.4%)	1066 (94.8%)	5830 (94.9%)
Incident	73 (3.8%)	100 (6.2%)	83 (5.6%)	59 (5.2%)	315 (5.1%)
Endometrial malignant cancer					
Non-case	1839 (95.0%)	1545 (96.4%)	1434 (96.7%)	1067 (94.8%)	5885 (95.8%)
Incident	96 (5.0%)	57 (3.6%)	49 (3.3%)	58 (5.2%)	260 (4.2%)
Glioma malignant cancer					
Non-case	1910 (98.7%)	1572 (98.1%)	1458 (98.3%)	1110 (98.7%)	6050 (98.5%)
Incident	25 (1.3%)	30 (1.9%)	25 (1.7%)	15 (1.3%)	95 (1.5%)
Kidney malignant cancer					
Non-case	1874 (96.8%)	1553 (96.9%)	1445 (97.4%)	1106 (98.3%)	5978 (97.3%)
Incident	61 (3.2%)	49 (3.1%)	38 (2.6%)	19 (1.7%)	167 (2.7%)
Liver malignant cancer					
Non-case	1906 (98.5%)	1573 (98.2%)	1471 (99.2%)	1116 (99.2%)	6066 (98.7%)
Incident	29 (1.5%)	29 (1.8%)	12 (0.8%)	9 (0.8%)	79 (1.3%)
Lung malignant cancer					
Non-case	1775 (91.7%)	1475 (92.1%)	1363 (91.9%)	1014 (90.1%)	5627 (91.6%)
Incident	160 (8.3%)	127 (7.9%)	120 (8.1%)	111 (9.9%)	518 (8.4%)
Lymphoma malignant					
Non-case	1777 (91.8%)	1499 (93.6%)	1368 (92.2%)	1053 (93.6%)	5697 (92.7%)
Incident	158 (8.2%)	103 (6.4%)	115 (7.8%)	72 (6.4%)	448 (7.3%)
Melanoma malignant cancer					
Non-case	1865 (96.4%)	1567 (97.8%)	1387 (93.5%)	1056 (93.9%)	5875 (95.6%)
Incident	70 (3.6%)	35 (2.2%)	96 (6.5%)	69 (6.1%)	270 (4.4%)
Ovary malignant cancer					

Non-case	1879 (97.1%)	1559 (97.3%)	1433 (96.6%)	1081 (96.1%)	5952 (96.9%)
Incident	56 (2.9%)	43 (2.7%)	50 (3.4%)	44 (3.9%)	193 (3.1%)
**Pancreas malignant cancer**					
Non-case	1896 (98.0%)	1559 (97.3%)	1447 (97.6%)	1087 (96.6%)	5989 (97.5%)
Incident	39 (2.0%)	43 (2.7%)	36 (2.4%)	38 (3.4%)	156 (2.5%)
**Prostate malignant cancer**					
Non-case	1710 (88.4%)	1295 (80.8%)	1189 (80.2%)	1048 (93.2%)	5242 (85.3%)
Incident	225 (11.6%)	307 (19.2%)	294 (19.8%)	77 (6.8%)	903 (14.7%)
**Stomach malignant cancer**					
Non-case	1851 (95.7%)	1533 (95.7%)	1449 (97.7%)	1097 (97.5%)	5930 (96.5%)
Incident	84 (4.3%)	69 (4.3%)	34 (2.3%)	28 (2.5%)	215 (3.5%)
**Thyroid malignant cancer**					
Non-case	1870 (96.6%)	1572 (98.1%)	1475 (99.5%)	1121 (99.6%)	6038 (98.3%)
Incident	65 (3.4%)	30 (1.9%)	8 (0.5%)	4 (0.4%)	107 (1.7%)
**Upper aero-digestive tract malignant cancer**					
Non-case	1890 (97.7%)	1525 (95.2%)	1423 (96.0%)	1075 (95.6%)	5913 (96.2%)
Incident	45 (2.3%)	77 (4.8%)	60 (4.0%)	50 (4.4%)	232 (3.8%)
**Cancer follow-up time (years)**	8.63 (4.29)	10.4 (4.59)	9.27 (4.60)	9.39 (4.19)	9.38 (4.48)

Continuous variables presented as mean (standard deviation)

Table S6: Characteristics of the subcohort selected as comparator for the Dementia/ Alzheimer's disease case-cohort analysis.

	Spain	Overall
	(N=1894)	(N=1894)
Age (years)	49.8 (8.01)	49.8 (8.01)
sex		
Male	717 (37.9%)	717 (37.9%)
Female	1177 (62.1%)	1177 (62.1%)
Level of Education		
None	679 (35.9%)	679 (35.9%)
Primary school completed	738 (39.0%)	738 (39.0%)
Technical/professional school	141 (7.4%)	141 (7.4%)
Secondary school	109 (5.8%)	109 (5.8%)
Longer education (incl. University deg.)	214 (11.3%)	214 (11.3%)
Missing	13 (0.7%)	13 (0.7%)
BMI (kg/m2)	28.4 (4.45)	28.4 (4.45)
Alcohol consumption (grams/day)	13.6 (22.0)	13.6 (22.0)
Smoking Status		
Never	1068 (56.4%)	1068 (56.4%)
Former	326 (17.2%)	326 (17.2%)
Smoker	500 (26.4%)	500 (26.4%)
Physical activity (METS recreational and household activity)	96.2 (56.2)	96.2 (56.2)
Cambridge physical activity index		
Inactive	717 (37.9%)	717 (37.9%)
Moderately inactive	611 (32.3%)	611 (32.3%)
Moderately active	337 (17.8%)	337 (17.8%)
Active	229 (12.1%)	229 (12.1%)
Missing	0 (0%)	0 (0%)
Healthy score for diet (range 0-54)	30.9 (5.88)	30.9 (5.88)
Missing	40 (2.1%)	40 (2.1%)

**Healthy lifestyle index (range 0 - 20)**	12.1 (3.39)	12.1 (3.39)
**Missing**	42 (2.2%)	42 (2.2%)
**Prevalent MI/Stroke reported**		
**No**	1834 (96.8%)	1834 (96.8%)
**Yes**	60 (3.2%)	60 (3.2%)
**InterAct prevalent diabetes reported**		
**No**	1742 (92.0%)	1742 (92.0%)
**Yes**	118 (6.2%)	118 (6.2%)
**Missing**	34 (1.8%)	34 (1.8%)
**Alzheimer’s disease**		
**Non-case**	1811 (95.6%)	1811 (95.6%)
**Incident**	83 (4.4%)	83 (4.4%)
**Alzheimer’s disease Follow up time (yrs)**	21.8 (3.80)	21.8 (3.80)
**Dementia**		
**Non-case**	1779 (93.9%)	1779 (93.9%)
**Incident**	115 (6.1%)	115 (6.1%)
**Dementia Follow up time (yrs)**	21.9 (3.82)	21.9 (3.82)
**Death event**		
**Non-case**	1729 (91.3%)	1729 (91.3%)
**Incident**	165 (8.7%)	165 (8.7%)
**Any first CVD event (CHD/Stroke) (fatal/non-fatal)**		
**Non-case**	1807 (95.4%)	1807 (95.4%)
**Incident**	87 (4.6%)	87 (4.6%)
**Type 2 diabetes status**		
**Non-case**	1727 (91.2%)	1727 (91.2%)
**Incident**	133 (7.0%)	133 (7.0%)
**Missing**	34 (1.8%)	34 (1.8%)
**Any malignant cancer**		
**Non-case**	1720 (90.8%)	1720 (90.8%)
**Incident**	174 (9.2%)	174 (9.2%)

Table S7: Characteristics of the dementia case sample.

	Spain	Overall
	(N=1043)	(N=1043)
**Age (years)**	58.4 (5.56)	58.4 (5.56)
**sex**		
**Male**	351 (33.7%)	351 (33.7%)
**Female**	692 (66.3%)	692 (66.3%)
**Level of Education**		
**None**	628 (60.2%)	628 (60.2%)
**Primary school completed**	290 (27.8%)	290 (27.8%)
**Technical/professional school**	36 (3.5%)	36 (3.5%)
**Secondary school**	33 (3.2%)	33 (3.2%)
**Longer education (incl. University deg.)**	45 (4.3%)	45 (4.3%)
**Missing**	11 (1.1%)	11 (1.1%)
**BMI (kg/m2)**	29.8 (4.18)	29.8 (4.18)
**Alcohol consumption (grams/day)**	11.2 (24.8)	11.2 (24.8)
**Smoking Status**		
**Never**	759 (72.8%)	759 (72.8%)
**Former**	133 (12.8%)	133 (12.8%)
**Smoker**	150 (14.4%)	150 (14.4%)
**Missing**	1 (0.1%)	1 (0.1%)
**Physical activity (METS recreational and household activity)**	105 (56.7)	105 (56.7)
**Cambridge physical activity index**		
**Inactive**	478 (45.8%)	478 (45.8%)
**Moderately inactive**	330 (31.6%)	330 (31.6%)
**Moderately active**	149 (14.3%)	149 (14.3%)
**Active**	86 (8.2%)	86 (8.2%)
**Missing**	0 (0%)	0 (0%)

**Healthy score for diet (range 0-54)**	31.4 (5.89)	31.4 (5.89)
**Missing**	32 (3.1%)	32 (3.1%)
**Healthy lifestyle index (range 0 - 20)**	12.7 (3.06)	12.7 (3.06)
**Missing**	38 (3.6%)	38 (3.6%)
**Prevalent MI/Stroke reported**		
**No**	1016 (97.4%)	1016 (97.4%)
**Yes**	27 (2.6%)	27 (2.6%)
**InterAct prevalent diabetes reported**		
**No**	876 (84.0%)	876 (84.0%)
**Yes**	147 (14.1%)	147 (14.1%)
**Missing**	20 (1.9%)	20 (1.9%)
**Dementia**		
**Non-case**	0 (0%)	0 (0%)
**Incident**	1043 (100%)	1043 (100%)
**Dementia Follow up time (yrs)**	18.4 (4.56)	18.4 (4.56)
**Alzheimer's disease**		
**Non-case**	334 (32%)	334 (32%)
**Incident**	709 (68%)	709 (100%)
**Alzheimer's disease Follow up time (yrs)**	18.6 (4.51)	18.6 (4.51)
**Death event**		
**Non-case**	889 (85.2%)	889 (85.2%)
**Incident**	154 (14.8%)	154 (14.8%)
**Any first CVD event (CHD/Stroke) (fatal/non-fatal)**		
**Non-case**	955 (91.6%)	955 (91.6%)
**Incident**	88 (8.4%)	88 (8.4%)
**Type 2 diabetes status**		
**Non-case**	926 (88.8%)	926 (88.8%)
**Incident**	97 (9.3%)	97 (9.3%)
**Missing**	20 (1.9%)	20 (1.9%)
**Any malignant cancer**		

**Non-case**	932 (89.4%)	932 (89.4%)
**Incident**	111 (10.6%)	111 (10.6%)

Continuous variables presented as mean (standard deviation)

Table S8: Characteristics of the subcohort selected as comparator for the Parkinsons's disease case-cohort analysis.

	Germany	Italy	Spain	The Netherlands	United Kingdom	Overall
	(N=99)	(N=948)	(N=1527)	(N=813)	(N=613)	(N=4000)
**Age (years)**	52.7 (8.22)	51.4 (7.69)	49.5 (7.84)	55.0 (7.97)	59.3 (9.32)	52.6 (8.79)
**sex**						
**Male**	52 (52.5%)	329 (34.7%)	634 (41.5%)	119 (14.6%)	261 (42.6%)	1395 (34.9%)
**Female**	47 (47.5%)	619 (65.3%)	893 (58.5%)	694 (85.4%)	352 (57.4%)	2605 (65.1%)
**Level of Education**						
**None**	1 (1.0%)	6 (0.6%)	489 (32.0%)	0 (0%)	0 (0%)	496 (12.4%)
**Primary school completed**	24 (24.2%)	458 (48.3%)	639 (41.8%)	164 (20.2%)	228 (37.2%)	1513 (37.8%)
**Technical/professional school**	33 (33.3%)	134 (14.1%)	125 (8.2%)	233 (28.7%)	196 (32.0%)	721 (18.0%)
**Secondary school**	4 (4.0%)	229 (24.2%)	90 (5.9%)	236 (29.0%)	51 (8.3%)	610 (15.3%)
**Longer education (incl. University deg.)**	37 (37.4%)	108 (11.4%)	175 (11.5%)	177 (21.8%)	63 (10.3%)	560 (14.0%)
**Missing**	0 (0%)	13 (1.4%)	9 (0.6%)	3 (0.4%)	75 (12.2%)	100 (2.5%)
**BMI (kg/m2)**	25.5 (4.22)	25.4 (3.97)	28.2 (4.40)	25.5 (3.92)	25.6 (3.61)	26.5 (4.28)
**Alcohol consumption (grams/day)**	16.9 (17.7)	15.6 (20.0)	15.6 (23.3)	10.1 (14.4)	7.17 (9.58)	13.2 (19.4)
**Missing**	0 (0%)	12 (1.3%)	0 (0%)	1 (0.1%)	2 (0.3%)	15 (0.4%)
**Smoking Status**						
**Never**	53 (53.5%)	448 (47.3%)	821 (53.8%)	322 (39.6%)	256 (41.8%)	1900 (47.5%)
**Former**	29 (29.3%)	251 (26.5%)	270 (17.7%)	267 (32.8%)	216 (35.2%)	1033 (25.8%)

Smoker	16 (16.2%)	232 (24.5%)	436 (28.6%)	220 (27.1%)	91 (14.8%)	995 (24.9%)
Missing	1 (1.0%)	17 (1.8%)	0 (0%)	4 (0.5%)	50 (8.2%)	72 (1.8%)
**Physical activity (METS recreational and household activity)**	86.4 (46.6)	93.6 (58.3)	92.2 (56.3)	114 (50.9)	91.1 (53.2)	96.6 (55.8)
Missing	0 (0%)	13 (1.4%)	0 (0%)	45 (5.5%)	52 (8.5%)	110 (2.8%)
**Cambridge physical activity index**						
Inactive	15 (15.2%)	199 (21.0%)	522 (34.2%)	68 (8.4%)	201 (32.8%)	1005 (25.1%)
Moderately inactive	34 (34.3%)	403 (42.5%)	490 (32.1%)	201 (24.7%)	176 (28.7%)	1304 (32.6%)
Moderately active	27 (27.3%)	185 (19.5%)	310 (20.3%)	191 (23.5%)	94 (15.3%)	807 (20.2%)
Active	23 (23.2%)	148 (15.6%)	205 (13.4%)	308 (37.9%)	90 (14.7%)	774 (19.4%)
Missing	0 (0%)	13 (1.4%)	0 (0%)	45 (5.5%)	52 (8.5%)	110 (2.8%)
**Healthy score for diet (range 0-54)**	26.6 (6.11)	26.1 (6.35)	31.1 (5.75)	27.2 (6.48)	23.4 (6.13)	27.9 (6.74)
Missing	0 (0%)	31 (3.3%)	34 (2.2%)	24 (3.0%)	15 (2.4%)	104 (2.6%)
**Healthy lifestyle index (range 0 - 20)**	11.9 (2.94)	11.2 (3.39)	11.9 (3.49)	12.6 (3.05)	11.8 (2.69)	11.9 (3.30)
Missing	1 (1.0%)	37 (3.9%)	36 (2.4%)	86 (10.6%)	87 (14.2%)	247 (6.2%)
**Prevalent MI/Stroke reported**						
No	93 (93.9%)	939 (99.1%)	1480 (96.9%)	786 (96.7%)	585 (95.4%)	3883 (97.1%)
Yes	6 (6.1%)	9 (0.9%)	47 (3.1%)	27 (3.3%)	28 (4.6%)	117 (2.9%)
**InterAct prevalent diabetes reported**						
No	95 (96.0%)	927 (97.8%)	1408 (92.2%)	792 (97.4%)	593 (96.7%)	3815 (95.4%)
Yes	2 (2.0%)	14 (1.5%)	88 (5.8%)	17 (2.1%)	20 (3.3%)	141 (3.5%)
Missing	2 (2.0%)	7 (0.7%)	31 (2.0%)	4 (0.5%)	0 (0%)	44 (1.1%)

**Parkinson's disease**						
**Non-case**	95 (96.0%)	941 (99.3%)	1522 (99.7%)	810 (99.6%)	610 (99.5%)	3978 (99.5%)
**Incident**	4 (4.0%)	7 (0.7%)	5 (0.3%)	3 (0.4%)	3 (0.5%)	22 (0.6%)
**Parkinson's disease Follow up time (yrs)**	15.8 (2.67)	19.0 (6.51)	15.9 (1.87)	16.2 (2.78)	13.9 (3.06)	16.4 (4.15)
**Death event**						
**Non-case**	92 (92.9%)	894 (94.3%)	1392 (91.2%)	713 (87.7%)	436 (71.1%)	3527 (88.2%)
**Incident**	7 (7.1%)	54 (5.7%)	135 (8.8%)	100 (12.3%)	177 (28.9%)	473 (11.8%)
**Any first CVD event (CHD/Stroke) (fatal/non-fatal)**						
**Non-case**	97 (98.0%)	929 (98.0%)	1451 (95.0%)	758 (93.2%)	530 (86.5%)	3765 (94.1%)
**Incident**	2 (2.0%)	19 (2.0%)	76 (5.0%)	55 (6.8%)	83 (13.5%)	235 (5.9%)
**Type 2 diabetes status**						
**Non-case**	92 (92.9%)	914 (96.4%)	1385 (90.7%)	783 (96.3%)	597 (97.4%)	3771 (94.3%)
**Incident**	5 (5.1%)	27 (2.8%)	111 (7.3%)	26 (3.2%)	16 (2.6%)	185 (4.6%)
**Missing**	2 (2.0%)	7 (0.7%)	31 (2.0%)	4 (0.5%)	0 (0%)	44 (1.1%)
**Any malignant cancer**						
**Non-case**	93 (93.9%)	838 (88.4%)	1387 (90.8%)	720 (88.6%)	520 (84.8%)	3558 (89.0%)
**Incident**	6 (6.1%)	110 (11.6%)	140 (9.2%)	93 (11.4%)	93 (15.2%)	442 (11.1%)

Continuous variables presented as mean (standard deviation)

Table S9: Characteristics of the Parkinson's disease case sample.

	Germany	Italy	Spain	The Netherlands	United Kingdom	Overall
	(N=89)	(N=124)	(N=92)	(N=47)	(N=151)	(N=503)
**Age (years)**	56.9 (5.45)	56.1 (6.43)	56.5 (6.02)	58.9 (7.67)	65.9 (7.30)	59.5 (7.83)
**sex**						
**Male**	57 (64.0%)	55 (44.4%)	52 (56.5%)	5 (10.6%)	94 (62.3%)	263 (52.3%)
**Female**	32 (36.0%)	69 (55.6%)	40 (43.5%)	42 (89.4%)	57 (37.7%)	240 (47.7%)
**Level of Education**						
**None**	0 (0%)	4 (3.2%)	44 (47.8%)	0 (0%)	0 (0%)	48 (9.5%)
**Primary school completed**	27 (30.3%)	51 (41.1%)	37 (40.2%)	7 (14.9%)	53 (35.1%)	175 (34.8%)
**Technical/professional school**	25 (28.1%)	24 (19.4%)	5 (5.4%)	11 (23.4%)	45 (29.8%)	110 (21.9%)
**Secondary school**	5 (5.6%)	24 (19.4%)	2 (2.2%)	16 (34.0%)	17 (11.3%)	64 (12.7%)
**Longer education (incl. University deg.)**	32 (36.0%)	17 (13.7%)	4 (4.3%)	12 (25.5%)	14 (9.3%)	79 (15.7%)
**Missing**	0 (0%)	4 (3.2%)	0 (0%)	1 (2.1%)	22 (14.6%)	27 (5.4%)
**BMI (kg/m2)**	26.4 (3.36)	25.3 (3.60)	29.0 (3.36)	25.9 (3.76)	26.0 (3.81)	26.4 (3.80)
**Alcohol consumption (grams/day)**	19.4 (21.1)	19.3 (20.5)	16.6 (21.4)	11.3 (14.2)	7.26 (10.3)	14.4 (18.5)
**Missing**	0 (0%)	4 (3.2%)	0 (0%)	1 (2.1%)	0 (0%)	5 (1.0%)
**Smoking Status**						
**Never**	43 (48.3%)	59 (47.6%)	59 (64.1%)	29 (61.7%)	60 (39.7%)	250 (49.7%)
**Former**	34 (38.2%)	40 (32.3%)	14 (15.2%)	15 (31.9%)	67 (44.4%)	170 (33.8%)
**Smoker**	12 (13.5%)	21 (16.9%)	19 (20.7%)	3 (6.4%)	17 (11.3%)	72 (14.3%)
**Missing**	0 (0%)	4 (3.2%)	0 (0%)	0 (0%)	7 (4.6%)	11 (2.2%)
**Physical activity (METS recreational and household activity)**	95.7 (53.6)	90.1 (55.6)	81.5 (53.3)	108 (59.7)	88.9 (47.1)	90.8 (53.1)
**Missing**	0 (0%)	4 (3.2%)	0 (0%)	4 (8.5%)	9 (6.0%)	17 (3.4%)
**Cambridge physical activity index**						
**Inactive**	18 (20.2%)	26 (21.0%)	34 (37.0%)	2 (4.3%)	64 (42.4%)	144 (28.6%)
**Moderately inactive**	26 (29.2%)	58 (46.8%)	35 (38.0%)	11 (23.4%)	42 (27.8%)	172 (34.2%)
**Moderately active**	26 (29.2%)	24 (19.4%)	12 (13.0%)	12 (25.5%)	21 (13.9%)	95 (18.9%)
**Active**	19 (21.3%)	12 (9.7%)	11 (12.0%)	18 (38.3%)	15 (9.9%)	75 (14.9%)

Missing	0 (0%)	4 (3.2%)	0 (0%)	4 (8.5%)	9 (6.0%)	17 (3.4%)
**Healthy score for diet (range 0-54)**	28.2 (5.85)	26.7 (7.04)	31.8 (6.19)	28.4 (6.25)	23.0 (6.56)	26.9 (7.13)
Missing	2 (2.2%)	4 (3.2%)	4 (4.3%)	1 (2.1%)	3 (2.0%)	14 (2.8%)
**Healthy lifestyle index (range 0 - 20)**	11.9 (3.29)	11.1 (3.25)	11.8 (3.03)	12.9 (2.61)	12.0 (2.89)	11.8 (3.10)
Missing	3 (3.4%)	4 (3.2%)	5 (5.4%)	6 (12.8%)	21 (13.9%)	39 (7.8%)
**Prevalent MI/Stroke reported**						
No	86 (96.6%)	121 (97.6%)	91 (98.9%)	45 (95.7%)	138 (91.4%)	481 (95.6%)
Yes	3 (3.4%)	3 (2.4%)	1 (1.1%)	2 (4.3%)	13 (8.6%)	22 (4.4%)
**InterAct prevalent diabetes reported**						
No	77 (86.5%)	120 (96.8%)	84 (91.3%)	46 (97.9%)	137 (90.7%)	464 (92.2%)
Yes	8 (9.0%)	1 (0.8%)	8 (8.7%)	1 (2.1%)	12 (7.9%)	30 (6.0%)
Missing	4 (4.5%)	3 (2.4%)	0 (0%)	0 (0%)	2 (1.3%)	9 (1.8%)
**Parkinson's disease**						
Non-case	0 (%)	0 (%)	0 (%)	0 (%)	0 (%)	0 (%)
Incident	89 (100%)	124 (100%)	92 (100%)	47 (100%)	151 (100%)	503 (100%)
**Parkinson's disease Follow up time (yrs)**	11.3 (4.74)	11.9 (5.57)	10.8 (4.17)	9.70 (5.10)	6.48 (3.22)	9.77 (5.02)
**Death event**						
Non-case	75 (84.3%)	118 (95.2%)	75 (81.5%)	40 (85.1%)	53 (35.1%)	361 (71.8%)
Incident	14 (15.7%)	6 (4.8%)	17 (18.5%)	7 (14.9%)	98 (64.9%)	142 (28.2%)
**Any first CVD event (CHD/Stroke) (fatal/non-fatal)**						
Non-case	88 (98.9%)	121 (97.6%)	83 (90.2%)	43 (91.5%)	106 (70.2%)	441 (87.7%)
Incident	1 (1.1%)	3 (2.4%)	9 (9.8%)	4 (8.5%)	45 (29.8%)	62 (12.3%)
**Type 2 diabetes status**						
Non-case	81 (91.0%)	119 (96.0%)	81 (88.0%)	47 (100%)	141 (93.4%)	469 (93.2%)
Incident	4 (4.5%)	2 (1.6%)	11 (12.0%)	0 (0%)	8 (5.3%)	25 (5.0%)
Missing	4 (4.5%)	3 (2.4%)	0 (0%)	0 (0%)	2 (1.3%)	9 (1.8%)
**Any malignant cancer**						
Non-case	84 (94.4%)	113 (91.1%)	81 (88.0%)	42 (89.4%)	133 (88.1%)	453 (90.1%)
Incident	5 (5.6%)	11 (8.9%)	11 (12.0%)	5 (10.6%)	18 (11.9%)	50 (9.9%)
