# Supplementary material for: Associations of proteomic age clocks with lifestyle risk factors, incident chronic diseases and mortality in two European cohorts
Source: Nat Aging. 2026 Jun 29;6(7):1437–51. doi: 10.1038/s43587-026-01163-6 (PMC13375651; doi:10.1038/s43587-026-01163-6)
Supplement: Supplementary file 2 — Reporting Summary [file 43587_2026_1163_MOESM2_ESM.pdf]

nature portfolio

Corresponding author(s): Oliver Robinson

Last updated by author(s): May 14, 2026

# Reporting Summary

Nature Portfolio wishes to improve the reproducibility of the work that we publish. This form provides structure for consistency and transparency in reporting. For further information on Nature Portfolio policies, see our Editorial Policies and the Editorial Policy Checklist.

## Statistics

For all statistical analyses, confirm that the following items are present in the figure legend, table legend, main text, or Methods section.

| n/a | Confirmed | |
|---|---|---|
| ☐ | ☒ | The exact sample size (*n*) for each experimental group/condition, given as a discrete number and unit of measurement |
| ☐ | ☒ | A statement on whether measurements were taken from distinct samples or whether the same sample was measured repeatedly |
| ☐ | ☒ | The statistical test(s) used AND whether they are one- or two-sided<br>*Only common tests should be described solely by name; describe more complex techniques in the Methods section.* |
| ☐ | ☒ | A description of all covariates tested |
| ☐ | ☒ | A description of any assumptions or corrections, such as tests of normality and adjustment for multiple comparisons |
| ☐ | ☒ | A full description of the statistical parameters including central tendency (e.g. means) or other basic estimates (e.g. regression coefficient) AND variation (e.g. standard deviation) or associated estimates of uncertainty (e.g. confidence intervals) |
| ☐ | ☒ | For null hypothesis testing, the test statistic (e.g. *F*, *t*, *r*) with confidence intervals, effect sizes, degrees of freedom and *P* value noted<br>*Give P values as exact values whenever suitable.* |
| ☒ | ☐ | For Bayesian analysis, information on the choice of priors and Markov chain Monte Carlo settings |
| ☒ | ☐ | For hierarchical and complex designs, identification of the appropriate level for tests and full reporting of outcomes |
| ☐ | ☒ | Estimates of effect sizes (e.g. Cohen's *d*, Pearson's *r*), indicating how they were calculated |

*Our web collection on statistics for biologists contains articles on many of the points above.*

## Software and code

Policy information about availability of computer code

| | |
|---|---|
| Data collection | No software used |
| Data analysis | R version 4.3.1. The following R packages were used: Haven; tidyverse; grid; pheatmap; mice; forestplot;forestplotter; VennDiagram; glmnet; survival; labelled; table1; SomaDataIO; bigutilr. Additionally; clocks from the publication of Oh et al were calculated using the python organage package (https://github.com/hamiltonoh/organage). All R scripts for analysis are provided at https://github.com/oliverrobinson2025/Global-proteomic-age |

For manuscripts utilizing custom algorithms or software that are central to the research but not yet described in published literature, software must be made available to editors and reviewers. We strongly encourage code deposition in a community repository (e.g. GitHub). See the Nature Portfolio guidelines for submitting code & software for further information.

## Data

Policy information about availability of data

All manuscripts must include a data availability statement. This statement should provide the following information, where applicable:

- Accession codes, unique identifiers, or web links for publicly available datasets
- A description of any restrictions on data availability
- For clinical datasets or third party data, please ensure that the statement adheres to our policy

EPIC Data can be accessed by external researchers after submission of a research proposal and approval by the relevant EPIC working group and the EPIC steering

committee. Full details of the access policy and procedures for data access are provided here: https://epic.iarc.fr/access/

## Research involving human participants, their data, or biological material

Policy information about studies with human participants or human data. See also policy information about sex, gender (identity/presentation), and sexual orientation and race, ethnicity and racism.

| | |
|---|---|
| Reporting on sex and gender | We report using the term "sex " to describe demographic information of sample.<br><br>We have not presented analysis disaggregated by sex (apart from sex-specific cancers), as we have presented many other analyses already in the paper |
| Reporting on race, ethnicity, or other socially relevant groupings | We have not reported on race or ethnicity in the paper. As reported in the limitations the sample is almost exclusively White European ethnicity and therefore it was not meaningful to include race or ethnicity as a covariate. We have included level of schooling as an indicator of socio-economic position. |
| Population characteristics | The random subcohort, representative of the broader EPIC study and the general European population at the time of recruitment, included 4,115 subjects from Italy, Spain, the Netherlands and the UK. Mean age in the subcohort was 51.4 (standard deviation (SD): 8.6) years and 62% were female. Mean BMI was 26.9 (4.3), 25% were smokers and 14% had a university level education. During a mean follow-up time of 17.4 (2.9) years, 393 (9.6%) deaths occurred in the subcohort. |
| Recruitment | Recruitment of subjects was based on invitations from the general adult population residing in a given town or geographical area, defined by each study centre participating in the EPIC study. The actual study populations are samples of convenience of volunteers agreeing to participate, but not required to be random samples of defined populations; moreover, only some of the centres have maintained records of all the individuals invited to participate. Therefore, the population sample may be overrepresented by characteristics associated with willingness to participate in a research study. Full details are provided in DOI: 10.1079/PHN2002394 |
| Ethics oversight | EPIC was approved by the Ethics Committee of the International Agency for Research on Cancer (IARC), Lyon, France, and local ethics committees of the study centres. All participants provided written informed consent for the collection, storage, and individual follow-up of their data. |

Note that full information on the approval of the study protocol must also be provided in the manuscript.

# Field-specific reporting

Please select the one below that is the best fit for your research. If you are not sure, read the appropriate sections before making your selection.

☒ Life sciences ☐ Behavioural & social sciences ☐ Ecological, evolutionary & environmental sciences

For a reference copy of the document with all sections, see nature.com/documents/nr-reporting-summary-flat.pdf

# Life sciences study design

All studies must disclose on these points even when the disclosure is negative.

| | |
|---|---|
| Sample size | Sample size was selected to analyse proteins for a range of outcomes. Post-hoc power calculations indicate that we are powered (80%) to detect at an alpha of 0.001 (corrected for multiple outcomes) for a 1-point change in Proteomic age gap z-score, a hazard ratio of 1.05 for our most common outcome, all cause mortality (n = 10163, n events = 6441), and a hazard ratio of 1.58 for liver cancer (n = 4194, n events = 83), our rarest outcome. |
| Data exclusions | Participants from EPIC centres in Italy, Spain, UK, Netherland sand Germany were eligible. Participants were excluded from the EPIC somalogic study for the following reasons: No lifestyle baseline data ; No dietary baseline data; Prevalent cancer tumor; Date incident cancer tumor missing; No follow-up for vital status; Date of death missing; No blood sample collected; Not included in the Interact subcohort and without event (death, cancer, T2D, CVD, ND); History of MI/Stroke; Age at blood collection <35 or >75; Users of pill/hrt-ert at blood collection (or unknown use) ; Age at first menstrual period missing ; Full term pregnancy status unknown; Breast cancer tumor with unknown ER/PR status. |
| Replication | Replication was successfully performed on the mortality analysis in WHII study. |
| Randomization | The somalogic study was a case cohort design where a random subcohort was selected, based on prior work in the Interact EPIC study (doi: 10.1007/s00125-011-2182-9), to be representative of the of the wider eligible population in participating EPIC centres. The random subcohort was used as a comparator to cases samples where available eligible incident death, CVD, T2D, cancer and ND cases were randomly selected into each case sample. Covariates including age, sex, study centre and other risk factors were included in analysis. |
| Blinding | Blinding was not relevant to our study as it was a cohort design without group allocation. |

# Reporting for specific materials, systems and methods

We require information from authors about some types of materials, experimental systems and methods used in many studies. Here, indicate whether each material, system or method listed is relevant to your study. If you are not sure if a list item applies to your research, read the appropriate section before selecting a response.

## Materials & experimental systems

| n/a | Involved in the study |
|---|---|
| ☒ | ☐ Antibodies |
| ☒ | ☐ Eukaryotic cell lines |
| ☒ | ☐ Palaeontology and archaeology |
| ☒ | ☐ Animals and other organisms |
| ☒ | ☐ Clinical data |
| ☒ | ☐ Dual use research of concern |
| ☒ | ☐ Plants |

## Methods

| n/a | Involved in the study |
|---|---|
| ☒ | ☐ ChIP-seq |
| ☒ | ☐ Flow cytometry |
| ☒ | ☐ MRI-based neuroimaging |

## Plants

| | |
|---|---|
| Seed stocks | N/A |
| Novel plant genotypes | N/A |
| Authentication | N/A |
