# Supplementary material for: Associations of proteomic age clocks with lifestyle risk factors, incident chronic diseases and mortality in two European cohorts
Source: Nat Aging. 2026 Jun 29;6(7):1437–51. doi: 10.1038/s43587-026-01163-6 (PMC13375651; doi:10.1038/s43587-026-01163-6)
Supplement: Supplementary file 3 — Peer Review File [file 43587_2026_1163_MOESM3_ESM.pdf]

nature portfolio

Peer Review File

Associations of proteomic age clocks with lifestyle risk factors, incident chronic diseases, and mortality in in two European cohorts

Corresponding Author: Dr Oliver Robinson

This manuscript has been previously reviewed at another journal that is not operating a transparent peer review scheme.
The manuscript was considered suitable for publication without further review at Nature Aging.

Version 0:

Reviewer comments:

Reviewer #1

(Remarks to the Author)
Thank you for the revised manuscript. The authors have made substantial improvements, and I have no further comments at this time.

(Remarks on code availability)

Reviewer #2

(Remarks to the Author)
Robinson et al. comprehensively evaluated the associations between existing SomaScan-based proteomic clocks and both mortality and incident chronic diseases in a large, independent cohort. The findings of the present study offer robust external validation of established proteomic clocks, reinforcing their utility as biomarkers of biological aging in independent cohorts. I have only a major concern regarding the proposed global age gap and its implications.

The authors introduce a global age gap metric, defined as the mean of predicted age gaps derived from five previously developed clocks. They report that this global age gap demonstrates stronger associations with mortality and multiple incident diseases compared to individual clock estimates. However, the observed improvement in predictive performance appears marginal (per age-gap z-score) in the Fig 3&4, with minimal absolute differences between the global age clock and individual clocks (particularly the Tanaka and Oh clocks) and the significance of improvement lacks statistical justification. Therefore, the demonstration of these differences should be improved; otherwise, it may undermine the validity of downstream analyses focused on the global age gap and limit its clinical applicability, especially given its reliance on a larger number of protein measurements than individual clocks.

Additionally, this study found that proteomic age provided similar performance in predicting mortality to classical lifestyle risk factors, and that predictive power was further improved when risk factors and proteomic age gaps were combined, indicating that proteomic age provides additional information on mortality risk. Nevertheless, the incremental power of the global age gap over individual clocks, such as the Oh clock, remains negligible (Figure 6J). These results may again challenge the rationale for developing a global age gap metric and diminish its practical utility.

Minor:
Typo appears on line 386.
References 58–60, cited in lines 661–665, are absent from the reference list.

(Remarks on code availability)
The scripts in the codebook are accessible and numbered to reflect the order they should be run.

Reviewer #3

(Remarks to the Author)
Thank you for the possibility to review this interesting manuscript. The manuscript “Associations of proteomic age clocks with lifestyle risk factors, incident chronic diseases, and mortality in two European cohorts” investigates the relationship between proteomic aging clocks and lifestyle factors, disease incidence, and mortality using large-scale SomaScan-based proteomics data from the EPIC cohort, with replication of the mortality results in the Whitehall II study. The authors compare multiple previously published aging clocks and propose a global proteomic age metric that combines them by taking an unweighted mean. The topic is timely and relevant, given the growing interest in proteomics and its potential in aging biomarkers. However, some conceptual, analytical, and reporting issues limit the interpretability of the work.

We recommend that major revisions be made to the following:

1) Please provide a clearer statement of the study’s rationale and novelty relative to previous work on proteomic aging clocks. For example, the novelty of comparing the existing proteomic aging clocks and the rationale behind aggregating the clocks’ predictions into a Global aging clock should be further elaborated. The authors speculate that the advantage is in reducing bias, and this should be explicitly demonstrated.
2) Please introduce and contextualize the proteomic clocks included in the study, including the selection of the clocks in the study, broad differences in the clocks’ training populations (as shown in Figure 2A and noted in the Introduction: “the influence of different training populations on clock performance is unclear”), and training targets (e.g., age vs. frailty).
3) Please address the miscalibration and age-dependent performance of the aging clocks. Figure 2B indicates that e.g. predictions from the Tanaka clock appear systematically lower than chronological age, and the Sathyan clock shows smaller age gaps for younger compared to older individuals. While the use of a local regression curve mitigates this for the age gaps, including the Global age gap, the Global predicted age is likely still influenced by such miscalibration.

We recommend that minor revisions be made to the following:

1) Figure 1: Please ensure that the reported number of individuals after applying the exclusion criteria is consistent with the numbers before exclusion and those excluded. Clarify the meaning of the dashed arrow (pointing to the PD comparison subcohort) and the yellow dashed boxes.
2) Please specify which background gene set was used in the gene enrichment analyses, presumably all SomaScan-measured proteins.
3) Please include a brief description of the age clusters in the Results section to make the manuscript easier to follow, and expand the explanation in the Methods section by describing the difference between clusters 1 and 3.
4) Please state whether the Global predicted age and age gap are aggregating unstandardized or z-standardized values, and what the implications of this are.
5) Please specify whether “age” refers to age at the time of proteomic measurement
6) Please clarify how menopausal status was handled in the analyses, or, if it was not used, consider rephrasing the relevant section in the Methods.
7) Please specify how individuals who died during the follow-up are considered in the disease incidence analyses.
8) Please revise the code for readability and reproducibility. Please additionally provide code for generating data for the subcohorts, in particular for how the follow-up time was defined.
9) Please check the manuscript carefully for spelling and typographical errors.

(Remarks on code availability)

Version 1:

Reviewer comments:

Reviewer #2

(Remarks to the Author)
Thanks for the response. The authors have substantially improved the manuscript, and I have no additional comments.

(Remarks on code availability)

Reviewer #3

(Remarks to the Author)
Thank you for the revisions and detailed responses to the comments. Several points have been clarified, in particular regarding motivating the study, contextualizing the proteomic clocks included in the study, and improving the code. However, some important conceptual, analytical, and reporting issues would benefit from further clarification.

Revisions are recommended for the following points:

Precision and strength of association in mortality prediction for the Global age gap. In the revised manuscript, the authors state: “We show that a Global age gap, …, provides slightly stronger and more precise associations with mortality” (Discussion) and “the global proteomic age gap … showed the strongest positive association of all tested clocks with all-cause mortality” (Abstract). While the point estimate for the Global age gap is larger than for individual clocks, this has not been formally tested, and the largely overlapping confidence intervals suggest that the associations are broadly comparable. In addition, the confidence intervals for the association between mortality and the Global age gap (in Figure 3A) appear to be slightly wider compared to some of the published clocks. Please consider revising the wording (including in the abstract) to avoid implying improved precision or a clearly stronger association than the other clocks.

Interpretation of systematic age underprediction. In response to the comment about misclassification of the aging clocks in EPIC, the authors state: “The Tanaka and Lehallier clocks both provided an underprediction of age in EPIC, which can be attributed to use of earlier Somascan versions that could not be scaled to the intensity values of the protein data in EPIC.” The use of a different SomaScan platform appears to only apply to the Tanaka clock, based on Figure 1A. Please clarify this point in the text. Moreover, even in the presence of platform differences, it would be helpful to further clarify why this would be expected to result in systematic underprediction of chronological age.

Construction and interpretation of the Global age gap and the Global predicted age. The authors have clarified that the Global age gap is computed as the mean of the five unstandardized age gaps. A presumed consequence of this is that clocks with larger variance in their age gaps have a larger influence on the Global age gap. To aid interpretation, the authors should consider 1) including correlations between each age gap from the published clocks and the Global age gap in Figure 2C, and 2) clarifying how the Global predicted age, as shown in Figure 2B, was computed in the Methods.

Reporting and interpreting C-indices.
The authors state: “...the concordance index improved to 0.730 (95% CI: 0.718-0.742), similar to the improvement on addition of only the Global age gap (0.726, 95% CI: 0.718 -0.742).” This may be a reporting error in the latter, as the confidence interval is asymmetrical and differs from the one reported in Fig 6J. Please confirm that the correct confidence intervals are reported for both C-indices.
The manuscript reports: “The concordance index significantly improved (log-likelihood ratio test: p < 10-16) to 0.739 (95% CI: 0.727-0.750), when risk factors and the Global age gap were combined (Figure 6J).” and “We found that proteomic age provided similar performance in predicting mortality to classical lifestyle risk factors, and that predictive power was further improved when risk factors and proteomic age gaps were combined, indicating that proteomic age provides additional information on mortality risk.” Please clarify whether the log-likelihood ratio test was performed against the sex-and-age-only model or the risk-factor-based model, and adjust the statement accordingly. In addition, please rephrase the statement to refer to a significant improvement in model fit instead of discrimination, given that the log-likelihood test does not test differences in C-indices themselves. Moreover, in response to Reviewer 2, the authors state that formal comparison of C-indices across models is not appropriate given the study design. This seems difficult to reconcile with the use of a log-likelihood test to support claims of improved predictive performance, as the log-likelihood test would be expected to be subject to similar considerations regarding weighting and stratification. The authors could consider estimating uncertainty around the C-indices using a resampling approach (e.g., bootstrap), which would allow formal comparison of C-indices across models and would directly address the distinction between improvement in model fit and improvement in discrimination.

Choice of background set in the gene enrichment analysis. The authors have updated the Methods to state that enrichment was performed against all genes in KEGG, GO, and Reactome. Because SomaScan is a targeted proteomics platform, this choice of background may primarily reflect platform composition rather than biological signal. The authors are encouraged to repeat the enrichment analysis using the set of all SomaScan-measured proteins as the reference background to assess whether the reported pathway enrichments persist after accounting for platform selection bias.

Measures of uncertainty. Please provide measures of uncertainty (e.g., p-values or confidence intervals) where correlations between age gaps across clocks are presented

(Remarks on code availability)

Version 2:

Reviewer comments:

Reviewer #3

(Remarks to the Author)

(Remarks on code availability)
I would like to thank the authors for their careful revisions. I have no further comments.

Open Access This Peer Review File is licensed under a Creative Commons Attribution 4.0 International License, which permits use, sharing, adaptation, distribution and reproduction in any medium or format, as long as you give appropriate credit to the original author(s) and the source, provide a link to the Creative Commons license, and indicate if changes were made.
In cases where reviewers are anonymous, credit should be given to 'Anonymous Referee' and the source.
The images or other third party material in this Peer Review File are included in the article's Creative Commons license, unless indicated otherwise in a credit line to the material. If material is not included in the article's Creative Commons license and your intended use is not permitted by statutory regulation or exceeds the permitted use, you will need to obtain permission directly from the copyright holder.
To view a copy of this license, visit https://creativecommons.org/licenses/by/4.0/

12 December 2025

Dear Dr Aman,

Thank you for considering our manuscript "*Associations of proteomic age clocks with lifestyle risk factors, mortality and incident chronic diseases, and mortality in in two European cohorts*" (NATAGING-A10903-T) for publication in Nature Aging.

Below you will find point-by-point responses to the reviewers, written in blue. We are grateful for the further insights provided by the reviewers and yourself, and we hope you will find that this revision manuscript is further improved.

We look forward to hearing from you in due course.

Yours sincerely,

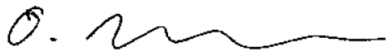

Oliver Robinson

Associate Professor in Molecular Epidemiology,

School of Public Health, Imperial College London

~~~~~~

Reviewers' Comments:

Reviewer #1 (Remarks to the Author):

Thank you for the revised manuscript. The authors have made substantial improvements, and I have no further comments at this time.

Thank you for the positive evaluation of our revised manuscript

Reviewer #2 (Remarks to the Author):

Robinson et al. comprehensively evaluated the associations between existing SomaScan-based proteomic clocks and both mortality and incident chronic diseases in a large, independent cohort. The findings of the present study offer robust external validation of established proteomic clocks, reinforcing their utility as biomarkers of biological aging in independent cohorts. I have only a major concern regarding the proposed global age gap and its implications.

Thank you for your critical review and positive comments.
~~~~~~

The authors introduce a global age gap metric, defined as the mean of predicted age gaps derived from five previously developed clocks. They report that this global age gap demonstrates stronger associations with mortality and multiple incident diseases compared to individual clock estimates. However, the observed improvement in predictive performance appears marginal (per age-gap z-score) in the Fig 3&4, with minimal absolute differences between the global age clock and individual clocks (particularly the Tanaka and Oh clocks) and the significance of improvement lacks statistical justification. Therefore, the demonstration of these differences should be improved; otherwise, it may undermine the validity of downstream analyses focused on the global age gap and limit its clinical applicability, especially given its reliance on a larger number of protein measurements than individual clocks.

Additionally, this study found that proteomic age provided similar performance in predicting mortality to classical lifestyle risk factors, and that predictive power was further improved when risk factors and proteomic age gaps were combined, indicating that proteomic age provides additional information on mortality risk. Nevertheless, the incremental power of the global age gap over individual clocks, such as the Oh clock, remains negligible (Figure 6J). These results may again challenge the rationale for developing a global age gap metric and diminish its practical utility.

We thank the reviewer for giving us the opportunity to clarify these points relating to the predictive ability of the Global clock compared to individual clocks. Differences in performance across models is typically judged by model discrimination, which in the Cox PH framework is generally done using the C-index. We recognise that formal statistical testing of differences in C-index across different (non-nested) model formulations would be preferable. However, unfortunately current methods are not yet appropriate for the study design of our analysis, which requires stratification by study centre, to ensure valid inferences by accounting for technical differences between study centres, and implementation of Prentice-weighting required for the case-cohort design. We searched for methods that would allow us to do this including the approach described here: doi: 10.1002/sim.6370, and a related approach based on time-dependent ROC curves (https://doi.org/10.1002/sim.5958), however no method is yet designed to implement these requirements. Despite being unable to conclusively demonstrate improved prediction of the Global clock compared to other clocks, we reiterate that our study is unique in testing and qualitatively comparing multiple clocks against risk of multiple diseases in a single longitudinal setting.

To display clearly the C-indexes across all the Conventional and Global age gap predictive models we have introduced a new Supplementary figure S14, and we have been careful not to state that the C-indexes are markedly different from the Global age gap. We have added:

“Similar concordance indexes were observed with the conventional age gaps, ranging from 0.735 for the Lehallier age gap and risk factor model to 0.738 for the Oh age gap and risk factor model (Supplementary figure S14).”

Regarding the differences in the associations we agree that the differences are not large (and we have mentioned this is the discussion) but we still believe it noteworthy that associations with mortality (as an indirect assessment of “biological age”) in risk-factor adjusted analyses are qualitatively greater than in each of the tested clocks (4% greater risk estimate than the Oh clock in EPIC and 2% greater than in Whitehall II) despite being derived from a simple arithmetic mean. Furthermore, we have clearly commented that all the included clocks perform well and similarly in associations with mortality and age-related diseases. We also believe it noteworthy to highlight that despite the consistency in associations with endpoints, associations with risk factors perform quite differently between clocks. For example, while the Oh clock performs excellently in associations with disease risk, it is not associated with smoking in EPIC, a key mortality risk factor, which would be problematic for practical use in interventional trials. The Global clock combines good disease/mortality prediction with sensitivity to some expected risk factors such as heavy alcohol use, smoking and physical inactivity. We have altered the following sentence in the first paragraph of the discussion:

“We found that the Global age gap, an unweighted average of age gaps derived from five previously developed clocks, was marginally more strongly associated with risk of mortality and several incident diseases than the individual clock estimates.”

And the final paragraph has been altered as follows:

“While we find that all conventional clocks trained in different populations showed similar patterns of association with mortality and disease risk, ~~W~~we ~~propose~~ show that a Global age clock, ~~constructed~~ combining proteomic age estimates from these clocks ~~trained in different populations, that may reduce biases related to the reference population and better capture the underlying biological ageing signal~~, provides slightly stronger and more precise associations with mortality.”

Despite using many proteins, the Global clock provides a solution for those who already have access to recent Somascan assays in their studies and need to decide how to apply an independently trained assessment of systematic ageing to their data. We agree that it would be more practical for future ageing biomarkers to move towards the incorporation of fewer proteins, but even in this case, the ensemble approach of the Global clock provides a useful framework for developing these future, more parsimonious ageing clocks.

We do not believe that the relatively small gain in predictive performance undermines the validity of the downstream analyses of the Global clock as these analyses are useful for all included clocks. The decision to focus on the Global clock in these analyses was also guided by practical aspects in terms of judicious

presentation of results, but they are relevant to all clocks in the study. Considering your comments, we have added the analysis of concordance with disease clusters for all conventional clocks to Supplementary figures S4-S8 and summarised the results of these additional analyses in a new panel in figure 4C:

"Similar patterns of associations were observed for the published conventional proteomic age gaps (Figure 4C, Supplementary figures S4-S8)."

Minor:

Typo appears on line 386.

Thank you -this has been corrected.

References 58–60, cited in lines 661–665, are absent from the reference list.

Thank you -this has been corrected.

Reviewer #2 (Remarks on code availability):

The scripts in the codebook are accessible and numbered to reflect the order they should be run.

Reviewer #3 (Remarks to the Author):

Thank you for the possibility to review this interesting manuscript. The manuscript "Associations of proteomic age clocks with lifestyle risk factors, incident chronic diseases, and mortality in two European cohorts" investigates the relationship between proteomic aging clocks and lifestyle factors, disease incidence, and mortality using large-scale SomaScan-based proteomics data from the EPIC cohort, with replication of the mortality results in the Whitehall II study. The authors compare multiple previously published aging clocks and propose a global proteomic age metric that combines them by taking an unweighted mean. The topic is timely and relevant, given the growing interest in proteomics and its potential in aging biomarkers. However, some conceptual, analytical, and reporting issues limit the interpretability of the work.

Thank you for your interest in our manuscript. We address your specific comments below

We recommend that major revisions be made to the following:

1) Please provide a clearer statement of the study's rationale and novelty relative to previous work on proteomic aging clocks. For example, the novelty of comparing the existing proteomic aging clocks and the rationale behind aggregating the clocks' predictions into a Global aging clock should be further elaborated. The authors speculate that the advantage is in reducing bias, and this should be explicitly demonstrated.

The final paragraph of the introduction has been re-written to better express the study rationale and novelty as follows:

"The Somascan-based clocks appear promising for the assessment of biological age and have been shown to predict mortality risk [12-15] and/or age-related phenotypes [14-16]. However, only the organ-specific clocks developed by Oh et al. [13] have been independently tested for risk of disease onset across multiple disease classes in a longitudinal setting [15]. In line with the Geroscience hypothesis, proteomic age gaps should have stronger associations with diseases that are observed to be more closely related to age itself. Furthermore, clocks trained in different populations may show differences in associations with both risk factors and disease risk due to the age-associated characteristics of those populations. The analysis of Somascan data within the European Prospective Investigation into Cancer and Nutrition (EPIC) study employs a powerful case-cohort design to assess risk of mortality and incidence of cardiometabolic diseases, neurodegenerative diseases and cancers in over 17,000 participants. Within EPIC, we sought to comprehensively assess associations of five previously published proteomic clocks and organ specific clocks with risk of mortality and 24 disease endpoints over up to 28 years of follow-up. We compared the strengths of associations across different clocks and all endpoints and further explored whether combining age-predictions from established clocks into a "Global" ensemble clock may improve risk associations. The findings on mortality were replicated in the British Whitehall II study. Finally, we tested associations of clocks with lifestyle risk factors, including a healthy lifestyle index."

We agree that we have speculated that the Global clock provides an advantage in reducing bias compared to individual clocks. However, we believe to conclusively demonstrate this bias in age gaps would require comparison to a "Gold standard" measure of systematic biological aging, which is unavailable as biological age is a latent, and in some respects conceptual, construct. Based on the available data within this study, the best approach we are aware of for inferring the role of bias in biological age assessment, is by judging qualitative differences in associations with remaining lifespan (i.e. time to mortality) and sensitivity to factors known to "accelerate ageing" in terms of shortening lifespan. We have shown qualitatively

stronger associations with mortality (albeit the difference is small – as acknowledged in our manuscript) in two separate cohorts and more coherent associations with risk factors in terms of expected directions of effect for the Global clock.

We have updated the discussion to adjust our tone in this regard and provide a more detailed discussion of one of the aims of the project, which was to assess the influence of training population:

"Other differences observed between the clocks may relate to how they were trained. The Wang clock provided the best absolute prediction of chronological age among published clocks in the EPIC sample, which has a similar age distribution to the training population used by Wang. The Sathyan clock was trained in only older participants, some with comorbidities, and had the strongest associations with dementia and Alzheimer's disease. The age-related changes captured by the Sathyan clock may be more relevant to these diseases, which have the oldest average age of disease onset. The Oh clock showed the strongest mortality prediction among published clocks, which may be due to the distinct machine learning algorithm employed, that combined models developed from bootstrapped resampling within the same population to improve generalizability. The Global clock takes a similar ensemble approach through averaging age predictions produced in different populations. We speculate that this may ~~appears to~~ ameliorate the influence of training population characteristics, and potentially also statistical error~~.~~, ~~through averaging age predictions produced in different populations.~~ This is evidenced by slightly larger and more precise associations with mortality in both the EPIC and Whitehall II studies, and more coherent associations with risk factors. As standardized proteomic data becomes more widely available around the world, future studies should train clocks in diverse populations, which may be further combined to provide ~~more precise~~ biological age estimates less influenced by cohort effects particular to a single training population."

And in the concluding paragraph:

"While we find that all conventional clocks trained in different populations showed similar patterns of association with mortality and disease risk, ~~W~~we ~~propose~~ show that a Global age clock, ~~constructed~~ combining proteomic age estimates from these clocks ~~trained in different populations, that may reduce biases related to the reference population and better capture the underlying biological ageing signal~~, provides slightly larger and more precise association with mortality."

2) Please introduce and contextualize the proteomic clocks included in the study, including the selection of the clocks in the study, broad differences in the clocks' training populations (as shown in Figure 2A and noted in the Introduction: "the influence of different training populations on clock performance is unclear"), and training targets (e.g., age vs. frailty).

We have added the following paragraph to the section on proteomic age calculation in the results:

"The characteristics of five "conventional" clocks included in the study are shown in Figure 2A. We included all published Somascan-based clocks, considered conventional as they were developed using all available proteins, as opposed to preselecting proteins as for organ-specific clocks, and trained directly on chronological age. The Tanaka clock was trained from a set of around 1,300 proteins among healthy participants free from chronic disease and cognitive or functional impairment aged 22-93 years from two US Baltimore based studies. The Lehallier clock was trained from a set of around 3,000 proteins among participants aged 18-76 years in the UK INTERVAL study, combined with participants aged 61-95 years in the US Longevity study of Ashkenazi Jews, 50% of whom were offspring of long-lived parents. The Sathyan clock was trained using around 5,000 proteins among the same older participants of the Longevity study, some of whom had comorbid conditions present as may be expected for their age range. The Oh clock was also trained using around 5,000 proteins in cognitively healthy controls, aged 27-104 years in the US Knight-ADRC study. Finally, the Wang clock was trained using around 5,000 proteins in the US multi-ethnic ARIC study in healthy participants aged 46-70 years free from major age-associated diseases."

3) Please address the miscalibration and age-dependent performance of the aging clocks. Figure 2B indicates that e.g. predictions from the Tanaka clock appear systematically lower than chronological age, and the Sathyan clock shows smaller age gaps for younger compared to older individuals. While the use of a local regression curve mitigates this for the age gaps, including the Global age gap, the Global predicted age is likely still influenced by such miscalibration.

We have added the following to the section on proteomic age calculation in the results:

"The Tanaka and Lehallier clocks both provided an underprediction of age in EPIC, which can be attributed to use of earlier Somascan versions that could not be scaled to the intensity values of the protein data in EPIC. Furthermore, all clocks to varying degrees exhibited age dependency in their predictive accuracy (Figure 2B), an issue observed generally in age prediction models [17]. These technical biases were corrected for by fitting a local regression line to derive an age gap representing the difference between proteomic and chronological age."

And we also added the following to the limitation section:

"We note that all included clocks, including the Global clock, showed age dependency in prediction performance, and while this was corrected for when deriving the age gap metric, further work is required to improve absolute age prediction for these clocks. "

We recommend that minor revisions be made to the following:

1) Figure 1: Please ensure that the reported number of individuals after applying the exclusion criteria is consistent with the numbers before exclusion and those excluded. Clarify the meaning of the dashed arrow (pointing to the PD comparison subcohort) and the yellow dashed boxes.

Thank you -we have corrected this numbering discrepancy in Figure 1and have clarified the meaning of the boxes and arrows in the figure caption:

"Yellow dotted boxes represent the two arms of the EPIC Somascan study, the neurodegenerative arm and the main arm that assesses risk of mortality, cancers, cardiovascular diseases and Type 2 diabetes. The comparison subcohort in each arm are different with subcohort samples in the neurodegenerative arm including both samples from the main arm and additional non-cases samples from Germany (blue dotted arrow)"

2) Please specify which background gene set was used in the gene enrichment analyses, presumably all SomaScan-measured proteins.

We have added the following to the methods:

"*We tested the over-representation of pathways in KEGG [58], GO (molecular function, cellular component, and biological process) [59], and Reactome [60] databases using hypergeometric tests against the whole gene sets available within each database."*

3) Please include a brief description of the age clusters in the Results section to make the manuscript easier to follow, and expand the explanation in the Methods section by describing the difference between clusters 1 and 3.

In results we have added:

"Positive directions of association with the Global proteomic age gap z-score were observed for almost all diseases in age clusters 1 and 3 (as defined by Kuan at al.17 as those with an exponential rate of disease onset increase with age, Figure 4C and 4D), while generally weaker or even negative directions of effect were observed for diseases in age clusters 4 (diseases with a more gradual relationship with age) and 5 (not age-related)."

In methods, we have added:

"Clusters 1 and 3 are distinguished by the median age of disease onset of 82 years and 69 years respectively."

4) Please state whether the Global predicted age and age gap are aggregating unstandardized or z-standardized values, and what the implications of this are.

We have clarified as follows:

"The Global age gap was similarly calculated as the mean of these five conventional clock unstandardized age gaps. The Age gaps were then z-scored for each published clock and the Global clock to account for differing variability, which were used as the primary exposure in all association analyses allowing direct comparison between the clocks"

5) Please specify whether "age" refers to age at the time of proteomic measurement

We have added the following to the methods:

"… the "Age gap" was calculated as described by Oh et al. [13] by fitting a local regression, that allows for non-linearity between predicted and chronological age at blood sampling using the lowess function…"

6) Please clarify how menopausal status was handled in the analyses, or, if it was not used, consider rephrasing the relevant section in the Methods.

Thank you for spotting this error. Menopause status was not used in the analysis and we have removed reference to this variable to avoid confusion

7) Please specify how individuals who died during the follow-up are considered in the disease incidence analyses.

We have added the following to the methods:

"Participants who died during follow-up were treated as censored in the incident disease analyses."

8) Please revise the code for readability and reproducibility. Please additionally provide code for generating data for the subcohorts, in particular for how the follow-up time was defined.

We have revised the code and added additional notation regarding lines for generating data for the subcohorts and how the follow-up time was defined.

9) Please check the manuscript carefully for spelling and typographical errors.

We have thoroughly checked the manuscript for typos

7 March 2026

Dear Dr Aman,

Thank you for considering our manuscript “*Associations of proteomic age clocks with lifestyle risk factors, incident chronic diseases, and mortality in in two European cohorts*” (NATAGING-A10903-A) for publication in Nature Aging.

Below you will find point-by-point responses to the reviewers, written in blue. We are grateful for the further insights provided by reviewer 3, and we hope you will find that the revised manuscript is further improved.

We look forward to hearing from you in due course.

Yours sincerely,

Oliver Robinson

Associate Professor in Molecular Epidemiology,

School of Public Health, Imperial College London

~~~~~~
~~~~~~

Reviewers' Comments:

Reviewer #2 (Remarks to the Author):

Thanks for the response. The authors have substantially improved the manuscript, and I have no additional comments.

Thank you very much for your helpful comments throughout the revision process, which we agree has improved the manuscript greatly.

Reviewer #3 (Remarks to the Author):

Thank you for the revisions and detailed responses to the comments. Several points have been clarified, in particular regarding motivating the study, contextualizing the proteomic clocks included in the study, and improving the code. However, some important conceptual, analytical, and reporting issues would benefit from further clarification.

Thank you so much for your thorough and perceptive review of our manuscript. Your comments have truly helped us sharpen and clarify our work. We respond to your remaining queries below.

Revisions are recommended for the following points:

Precision and strength of association in mortality prediction for the Global age gap. In the revised manuscript, the authors state: “We show that a Global age gap, …, provides slightly stronger and more precise associations with mortality” (Discussion) and “the global proteomic age gap … showed the strongest positive association of all tested clocks with all-cause mortality” (Abstract). While the point estimate for the Global age gap is larger than for individual clocks, this has not been formally tested, and the largely overlapping confidence intervals suggest that the associations are broadly comparable.

In addition, the confidence intervals for the association between mortality and the Global age gap (in Figure 3A) appear to be slightly wider compared to some of the published clocks. Please consider revising the wording (including in the abstract) to avoid implying improved precision or a clearly stronger association than the other clocks.

We agree that we were incorrect to claim greater precision of the Global clock associations, because as you say the confidence intervals are not narrower. This claim was based on an incorrect interpretation of the smaller p-values. We have revised the following sections:

Abstract:

“Assessment of biological ageing using proteomic clocks may enhance risk prediction and elucidate the molecular links between ageing and chronic diseases. We examined associations of plasma SomaScan-based proteomic clocks, including organ-specific clocks, with lifestyle risk factors, 24 incident chronic diseases and all-cause mortality among 17,473 participants of the European Prospective Investigation into Cancer and Nutrition (EPIC) study, with replication in the Whitehall II study (N = 6,240). During up to 28 years of follow-up, the Global proteomic age gap (a composite biological age acceleration score combining previously published clocks) ~~showed the strongest positive association of all tested clocks with all-cause mortality. Accelerated proteomic ageing~~ was associated with smoking, alcohol consumption, physical inactivity, and higher risk of cardiovascular diseases, dementia, and liver, upper aero-digestive tract, lung, and kidney cancers. Similar associations with mortality and disease were observed across published clocks trained in different populations. Lung, kidney, and stomach cancers were more strongly associated with related organ-specific age gaps. The predictive performance of proteomic clocks for mortality was comparable to that of classical lifestyle risk factors. In summary, proteomic clocks appear promising biomarkers of generalized age-related disease risk.”

Discussion:

"We speculate that this may ameliorate the influence of training population characteristics, and potentially also statistical error. This is evidenced by slightly larger ~~and more precise~~ associations with mortality in both the EPIC and Whitehall II studies, and more coherent associations with risk factors."

And

"While we find that all conventional clocks trained in different populations showed similar patterns of association with mortality and disease risk, we show that a Global age clock, combining proteomic age estimates from these clocks, provides a slightly larger ~~and more precise~~ association with mortality."

Interpretation of systematic age underprediction. In response to the comment about misclassification of the aging clocks in EPIC, the authors state: "The Tanaka and Lehallier clocks both provided an underprediction of age in EPIC, which can be attributed to use of earlier Somascan versions that could not be scaled to the intensity values of the protein data in EPIC." The use of a different SomaScan platform appears to only apply to the Tanaka clock, based on Figure 1A. Please clarify this point in the text. Moreover, even in the presence of platform differences, it would be helpful to further clarify why this would be expected to result in systematic underprediction of chronological age.

We have revised the text as follows to provide further explanation:

"The Tanaka and Lehallier clocks both provided an underprediction of age in EPIC. These clocks were trained on Somascan data~~, which can be attributed to use of earlier Somascan versions that could not be scaled to~~ for which the intensity values of the protein data in EPIC could not be appropriately scaled, due to use of an earlier Somascan version (Tanaka) or within population mean-scaling (Lehallier)."

Since it is not clear why lack of appropriate scaling should lead to systematic underprediction for both clocks, we have removed this statement. There does not appear to be a predominance of negative or positive model weights for either clock (Figure R1), while the sum of model weights is negative in the Tanaka model (-1.55) and positive in the Lehallier model (3.30). We speculate that the intensity values in EPIC may be systematically higher than or similar to the original Tanaka training set and systematically lower than the original Lehallier training set. However, this cannot be tested without access to the original datasets for comparison.

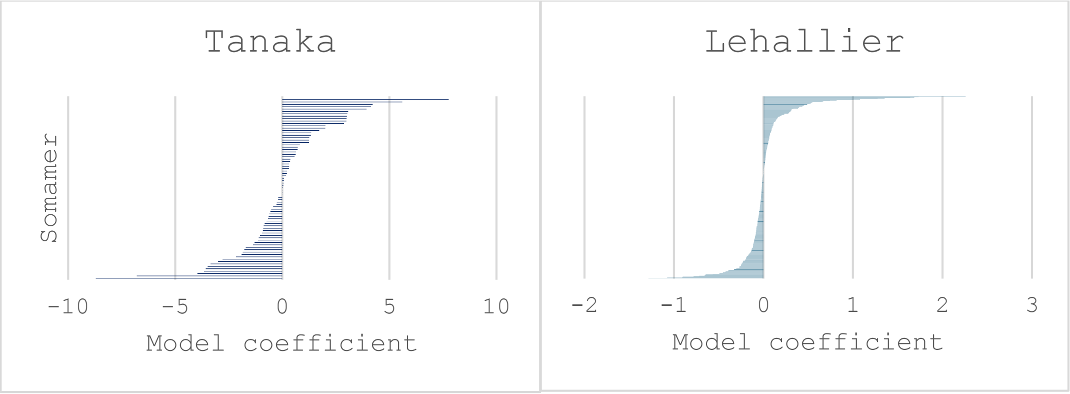

Figure R1: Overview of model weights in the Tanaka and Lehallier models.

Construction and interpretation of the Global age gap and the Global predicted age. The authors have clarified that the Global age gap is computed as the mean of the five unstandardized age gaps. A presumed consequence of this is that clocks with larger variance in their age gaps have a larger influence on the Global age gap. To aid interpretation, the authors should consider 1) including correlations between each age gap from the published clocks and the Global age gap in Figure 2C, and 2) clarifying how the Global predicted age, as shown in Figure 2B, was computed in the Methods.

We have updated Figure 2C to include the Global Age gap and added the following to the results section:

"To provide a single proteomic age ~~gap~~ estimation, we averaged both the proteomic ages and age gaps across the clocks to develop a "Global proteomic ~~age~~ clock". The correlation of Global proteomic age with chronological age was 0.89. Correlations of the Global age gap with the individual age gaps ranged from 0.68 (95% CI: 0.67, 0.69) for the Sathyan clock to 0.85 (95% CI: 0.85, 0.86) for the Lehallier clock (Figure 2C)."

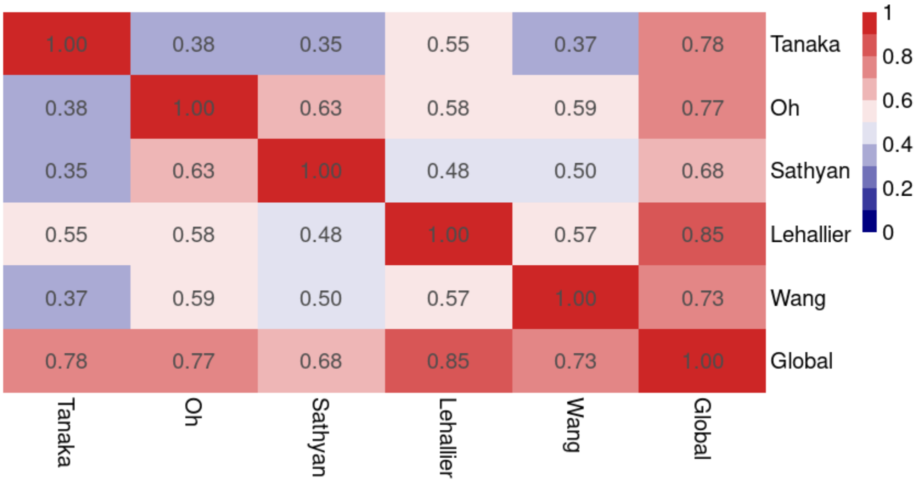

Figure 2C (Updated)

We updated the methods as follows:

"Additionally, we calculated a "Global" clock age as the mean of the predicted ages from the Tanaka, Lehallier, Sathyan, Oh, and Wang clocks. <u>This Global age metric was used to assess absolute chronological age prediction. Similarly,</u> a Global "age gap" was calculated as the mean of these five conventional clock unstandardized age gaps. The Age gaps were then z-scored for each published clock and the Global clock to account for differing variability, which were used as the primary exposure in all association analyses allowing direct comparison between the clocks"

For comparison Figure R2, shows correlations with the Global age gap, when calculated instead using standardized age gaps, and shows a similar range of correlations across the clock age gaps.

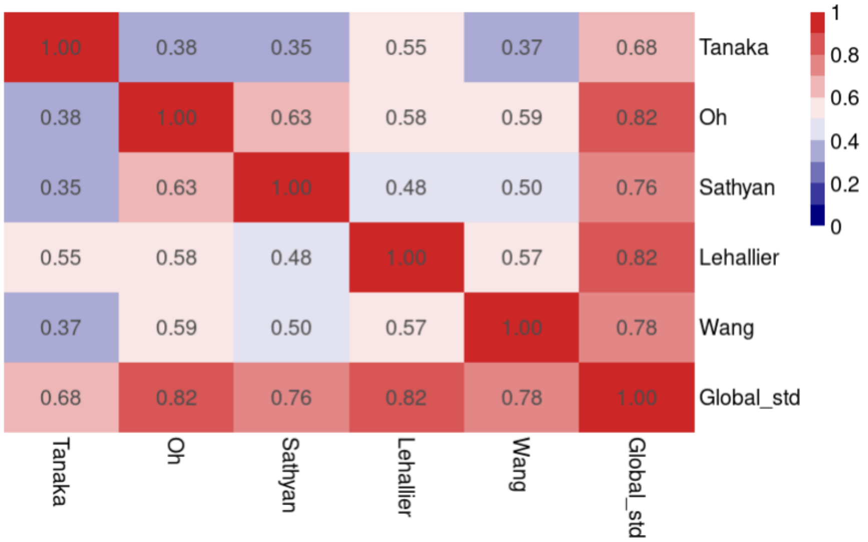

Figure R2: Correlation heatmap of proteomic age gaps. In this plot, "Global_std" is calculated as the mean of the standardised age gaps of the five published clocks

Reporting and interpreting C-indices.

The authors state: "...the concordance index improved to 0.730 (95% CI: 0.718-0.742), similar to the improvement on addition of only the Global age gap (0.726, 95% CI: 0.718 -0.742)." This may be a reporting error in the latter, as the confidence interval is asymmetrical and differs from the one reported in Fig 6J. Please confirm that the correct confidence intervals are reported for both C-indices.

The confidence intervals have now been corrected in the text. The confidence intervals have now changed very slightly, following the use of bootstrapping as suggested.

Following thorough internal review of the manuscript we have also identified and corrected the following reporting errors:

Figure 4C: The count of significant associations with endpoints shown in this table panel (summarising the FDR significant positive associations shown in Figure 3B) for the Oh clock has been corrected from “8” previously to “9”. The text referring to this table *(“Similar patterns of associations were observed for the published conventional proteomic age gaps (Figure 4C, Supplementary figures S4-S8).”)* remains unchanged.

The range of reported correlations of age gaps (referring to Figures 2C and s12, for which displayed correlations remains unchanged) was incorrectly reported. This has been corrected as follows (with CIs for specific correlations added at your request):

- *“Age gaps were moderately correlated across the conventional clocks, ranging from 0.~~37~~35 (95% confidence interval (CI): 0.34, 0.36) for Tanaka and ~~Wang~~ Sathyan age gaps to 0.63 (95% CI: 0.62, 0.64) for Sathyan and Oh age gaps (Figure 2C).”*
- *“… while other correlations between the organ-specific age gaps were weak to moderate, ranging from ~~0.08~~ -0.05 (kidney-heart, 95% CI: -0.07, -0.04) to ~~0.46~~ 0.40 (artery-adipose, 95% CI: 0.39, 0.41) (Figure S12).”*

We sincerely apologise for these errors. We have performed an additional review throughout the manuscript and found no further errors.

The manuscript reports: “The concordance index significantly improved (log-likelihood ratio test: p < 10-16) to 0.739 (95% CI: 0.727-0.750), when risk factors and the Global age gap were combined (Figure 6J).” and “We found that proteomic age provided similar performance in predicting mortality to classical lifestyle risk factors, and that predictive power was further improved when risk factors and proteomic age gaps were combined, indicating that proteomic age provides additional information on mortality risk.” Please clarify whether the log-likelihood ratio test was performed against the sex-and-age-only model or the risk-factor-based model, and adjust the statement accordingly. In addition, please rephrase the statement to refer to a significant improvement in model fit instead of discrimination, given that the log-likelihood test does not test differences in C-indices themselves.

Thank you for highlighting this distinction and the lack of clarity in the text. It has been updated as follows:

“The concordance index ~~significantly~~ improved to ~~(log-likelihood ratio test: p < 10-16)~~ 0.739 (95% CI: 0.727-0.750), when risk factors and the Global age gap were combined (Figure 6J). The Global age gap and risk factor model demonstrated a significant improvement in model fit compared to the risk factor only model (log-likelihood ratio test $p < 2.2 \times 10^{-16}$). “

Moreover, in response to Reviewer 2, the authors state that formal comparison of C-indices across models is not appropriate given the study design. This seems difficult to reconcile with the use of a log-likelihood test to support claims of improved predictive performance, as the log-likelihood test would be expected to be subject to similar considerations regarding weighting and stratification. The authors could

consider estimating uncertainty around the C-indices using a resampling approach (e.g., bootstrap), which would allow formal comparison of C-indices across models and would directly address the distinction between improvement in model fit and improvement in discrimination.

Thank you for the very helpful suggestion of applying bootstrapping. We would like to clarify that we were able to apply a log-likelihood ratio test for comparison to a risk factor only model because we were comparing nested models. This test is not applicable to comparison of non-nested models (i.e., comparing the Global clock-based model to the Oh clock-based model).

We have now calculated the Concordance indexes and confidence intervals (CIs) throughout using bootstrapping (500 bootstraps) and updated the figures figure 6J and S14 accordingly (Updated figure included below). The new CIs differ only slightly from the previous CIs and do not change the general interpretation.

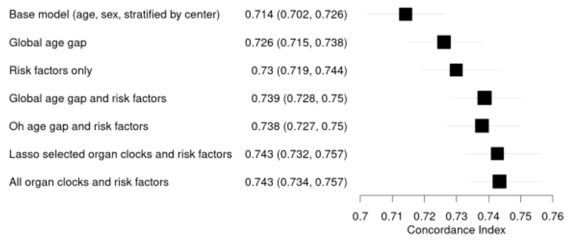

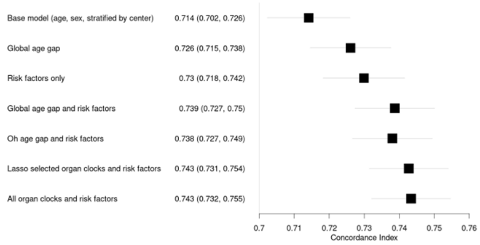

Updated figure 6J (Left, 500 Bootstrapped CIs). Previous figure 6J (Right, CIs derived from empirical standard error)

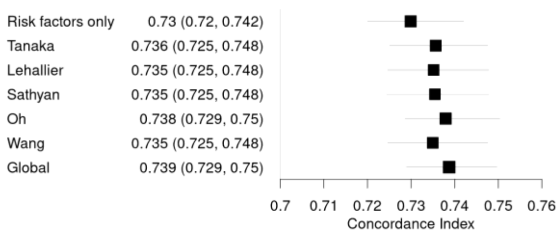

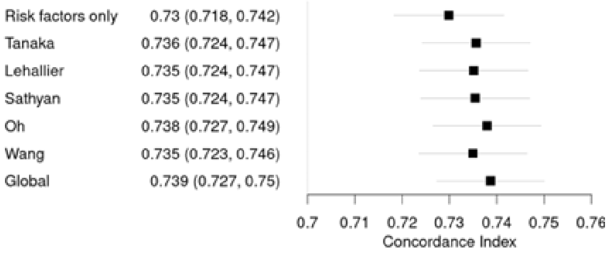

Updated figure S14 (Left, 500 Bootstrapped 95 CIs). Previous figure s14 (right, CIs derived from empirical standard error)

The following has been added to the methods:

“95% confidence intervals were calculated for all concordance indexes using 500 bootstraps.”

Choice of background set in the gene enrichment analysis. The authors have updated the Methods to state that enrichment was performed against all genes in KEGG, GO, and Reactome. Because SomaScan is a targeted proteomics platform, this choice of background may primarily reflect platform composition rather than biological signal. The authors are encouraged to repeat the enrichment analysis

using the set of all SomaScan-measured proteins as the reference background to assess whether the reported pathway enrichments persist after accounting for platform selection bias.

We introduced the enrichment analysis at the request of original Reviewer 1, who asked for biological characterisation of the processes captured by the clocks. We have chosen to include the whole gene sets as background, which is consistent with the methodology used in previous proteomic clock studies (e.g., Oh, H. S.-H. *et al.* Organ aging signatures in the plasma proteome track health and disease. *Nature* **624**, 164-172 (2023). https://doi.org:10.1038/s41586-023-06802-1). We believe this provides a more accurate assessment of the specific processes, out of all processes within the respective databases, that are targeted by the Somascan proteomic clock assessment. We acknowledge that the enrichments are to a large extent determined by the coverage of the Somascan platforms in addition to relevance to proteomic ageing. Indeed, re-running the analysis using only the Somascan measured proteins as background (Figure R3), showed much weaker enrichment as may be expected given the smaller background set. We note however that some pathways such as those related to the extracellular matrix, hormone signalling or processes related to PI3K are still highlighted. To highlight the role of platform specificity in the ORA we have updated the discussion as follows:

"ORA, which is partially driven by the proteomic coverage of the Somascan platforms, also indicated shared pathways related to the extracellular matrix, inflammation and hormonal activity across the tested clocks."

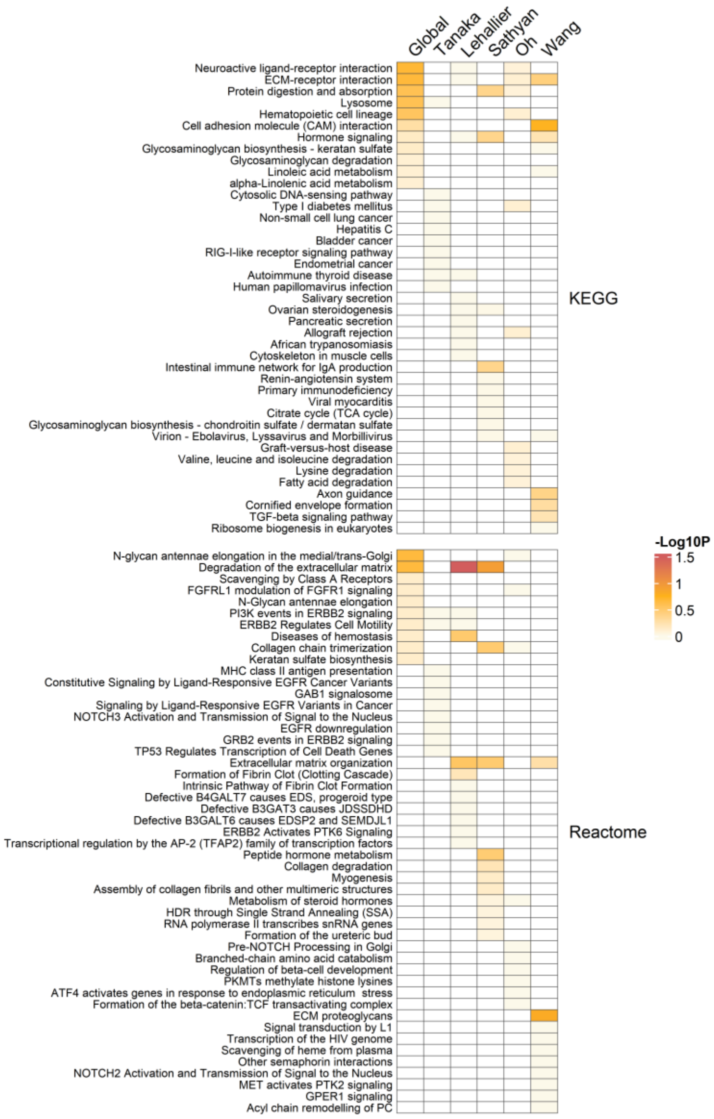

Figure R3: ***Enrichment of KEGG and Reactome pathways among proteins contained in the conventional clocks and the Global age clock, using only genes targeted by the respective Somascan platform as background. The top 10 enriched pathways for each clock are shown.***

Measures of uncertainty. Please provide measures of uncertainty (e.g., p-values or confidence intervals) where correlations between age gaps across clocks are presented

95% confidence intervals have now been added when correlations between age gaps are reported in the text.
